# s-Block metal complexes of superbulky (^*t*^Bu_3_Si)_2_N^−^: a new weakly coordinating anion?†

**DOI:** 10.1039/d3sc06896j

**Published:** 2024-02-20

**Authors:** Christian Knüpfer, Lukas Klerner, Jonathan Mai, Jens Langer, Sjoerd Harder

**Affiliations:** a Inorganic and Organometallic Chemistry, Friedrich-Alexander-Universität Erlangen-Nürnberg Egerlandstraße 1 91058 Erlangen Germany sjoerd.harder@fau.de

## Abstract

Sterically hindered amide anions have found widespread application as deprotonation agents or as ligands to stabilize metals in unusual coordination geometries or oxidation states. The use of bulky amides has also been advantageous in catalyst design. Herein we present s-block metal chemistry with one of the bulkiest known amide ligands: (*t*Bu_3_Si)_2_N^−^ (abbreviated: ^*t*Bu^N^−^). The parent amine (^*t*Bu^NH), introduced earlier by Wiberg, is extremely resistant to deprotonation (even with *n*BuLi/KO*t*Bu superbases) but can be deprotonated slowly with a blue Cs^+^/e^−^ electride formed by addition of Cs^0^ to THF. (^*t*Bu^N)Cs crystallized as a separated ion-pair, even without cocrystallized solvent. As salt-metathesis reactions with (^*t*Bu^N)Cs are sluggish and incomplete, it has only limited use as an amide transfer reagent. However, ball-milling with LiI led to quantitative formation of (^*t*Bu^N)Li and CsI. Structural characterization shows that (^*t*Bu^N)Li is a monomeric contact ion-pair with a relatively short N–Li bond, an unusual T-shaped coordination geometry around N and extremely short Li⋯Me anagostic interactions. Crystal structures are compared with Li and Cs complexes of less bulky amide ligands (iPr_3_Si)_2_N^−^ (^iPr^N^−^) and (Me_3_Si)_2_N^−^ (^Me^N^−^). DFT calculations show trends in the geometries and electron distributions of amide ligands of increasing steric bulk (^Me^N^−^ < ^iPr^N^−^ < ^*t*Bu^N^−^) and confirm that ^*t*Bu^N^−^ is a rare example of a halogen-free weakly coordinating anion.

## Abstract 2

The extremely challenging deprotonation of the amine (*t*Bu_3_Si)_2_NH led to a range of s-block metal complexes with superbulky (*t*Bu_3_Si)_2_N^−^. Depending on metal and solvent, (*t*Bu_3_Si)_2_N^−^ can be classified as a halogen-free weakly coordinating anion.

## Graphical abstract

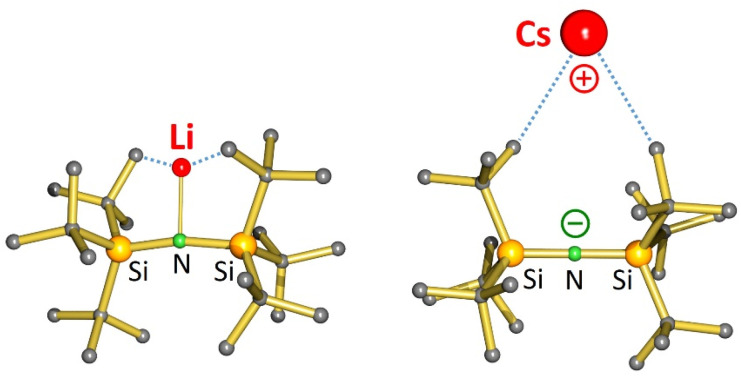

## Introduction

**Scheme 1:**
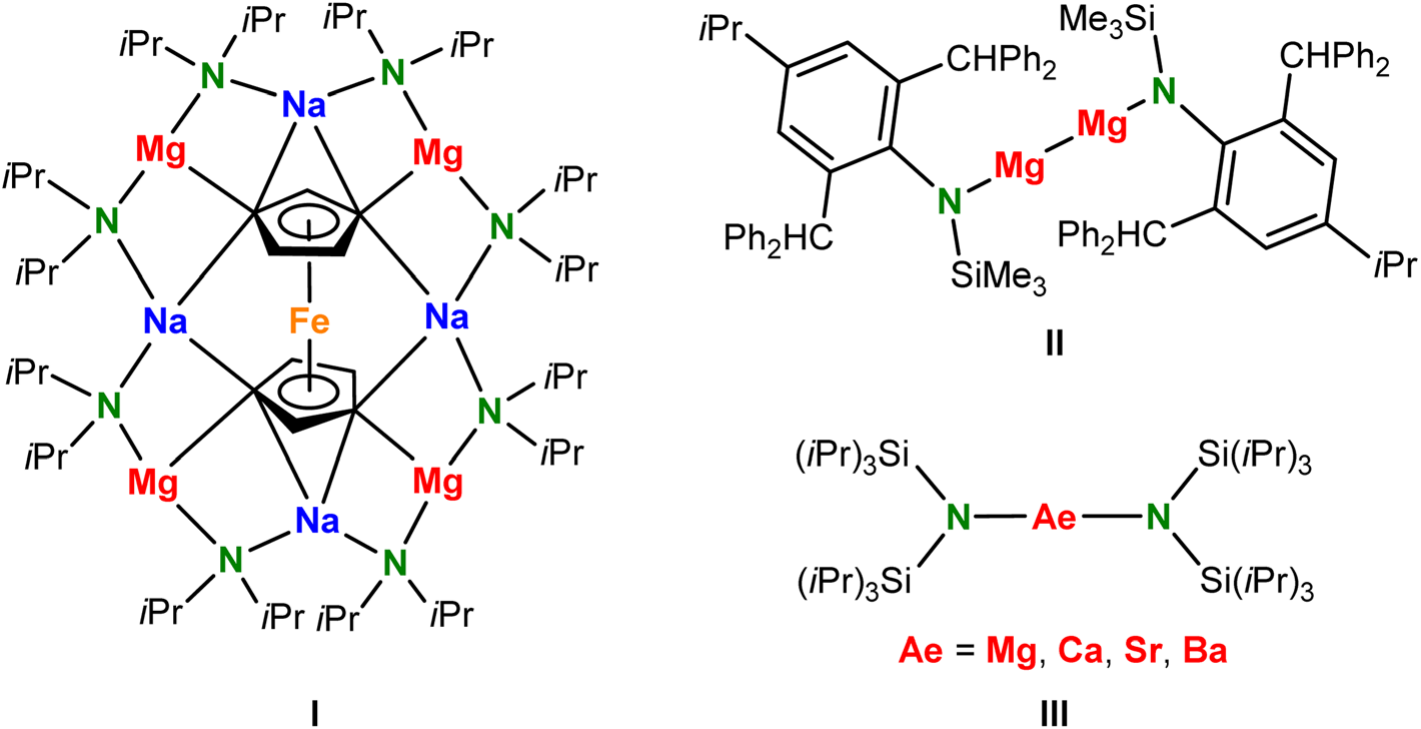
Formulae I–III.

According to the formal IUPAC definition,^1^ metal amide complexes should not be described as organometallic compounds. However, due to their crucial role and close relationship to organometallic chemistry, they are often considered as such.^2^ Especially the group 1 metal amides have found widespread applications as amide ligand transfer reagents to access metal amide complexes across the periodic table. Given the considerably higher electronegativity of N compared to C, amide anions are somewhat less Brønsted basic than corresponding carbanions. Despite this fundamental difference, carefully chosen, sterically hindered amide bases like lithium diisopropylamide (LDA) or lithium 2,2,6,6-tetramethylpiperidide (LiTMP) found fame as powerful non-nucleophilic deprotonation reagents.^3,4^ Amide anions are also markedly different from carbanions by the presence of two lone-pairs of electrons at N, which sets them apart as great bridging ligands. Their rich coordination chemistry has led to stunning examples of their unique deprotonation power^5,6^ and self-assembled aggregates in which multiply deprotonated substrates are embedded in a ring of metal cations that act as an inverse crown ether^7^ (*e.g.*I in Scheme 1).

Within the large range of amines, the silyl-substituted amine HN(SiMe_3_)_2_ (1,1,1,3,3,3-hexamethyldisilazane) is arguably the most common source for synthesis of metal complexes.^8,9^ The corresponding (Me_3_Si)_2_N^−^ ligand, often abbreviated as HMDS or N′′, is attractive while its Me_3_Si-substituents offer steric protection of the metal center and stabilize the neighbouring negative charge on N by polarization and negative hyperconjugation; *cf.* the p*K*_a_ values for HN(SiMe_3_)_2_ (25.8) and HNiPr_2_ (35.7).^10,11^ There are, however, also drawbacks of this ligand which are exemplified by N–Si bond cleavage^12,13^ or Me–Si deprotonation.^14^

In order to achieve greater stability and improve steric protection, a large range of bulkier silyl-substituted amides have been designed.^15^ Such bulky monodentate ligands achieved stabilization of low-oxidation-state Zn^I^ and Mg^I^ centers (*e.g.* in II).^16,17^ They also found application in the synthesis of nearly linear lanthanide metal complexes which have been studied extensively for their magnetic properties.^18^ Our interest in bulky amide ligands is related to their ability to lower the aggregation state of alkaline-earth (Ae) metal complexes^19,20^ and reduce the nuclearity of Ae metal hydride clusters.^21^ For this reason, bulky Ae metal amide complexes like Ae[N(SiiPr_3_)_2_] (III) are much more reactive in hydrogenation catalysis^21^ than Ae[N(SiMe_3_)_2_]_2_ catalysts^22,23^ and under controlled reaction conditions even found application as catalysts for Hydrogen-Isotope-Exchange (HIE) in aromatic substrates.^24^ As the activities of these catalysts increase with the size of the amide ligand, we are interested in s-block metal complexes with even bulkier amide ligands. It is, however, questionable what the limitations to the bulk of the substituents are. Herein, we report on the unusual coordination chemistry of the extremely bulky (*t*Bu_3_Si)_2_N^−^ anion (abbreviated: ^*t*Bu^N^−^), for which there is hitherto a complete lack of knowledge, and show comparisons with the smaller (iPr_3_Si)_2_N^−^ (^iPr^N^−^) and (Me_3_Si)_2_N^−^ (^Me^N^−^) anions.

## Results and discussion

### Syntheses

**Scheme 2:**
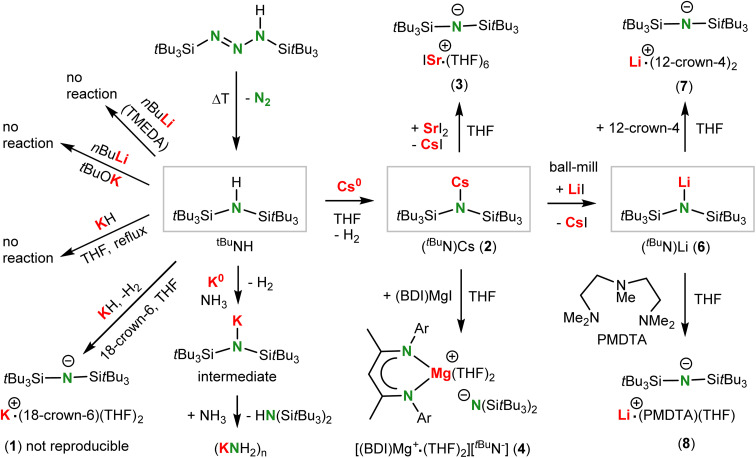
Synthesis and deprotonation of ^*t*Bu^NH and complexes with the bulky ^*t*Bu^N^−^ anion.

**Fig. 1:**
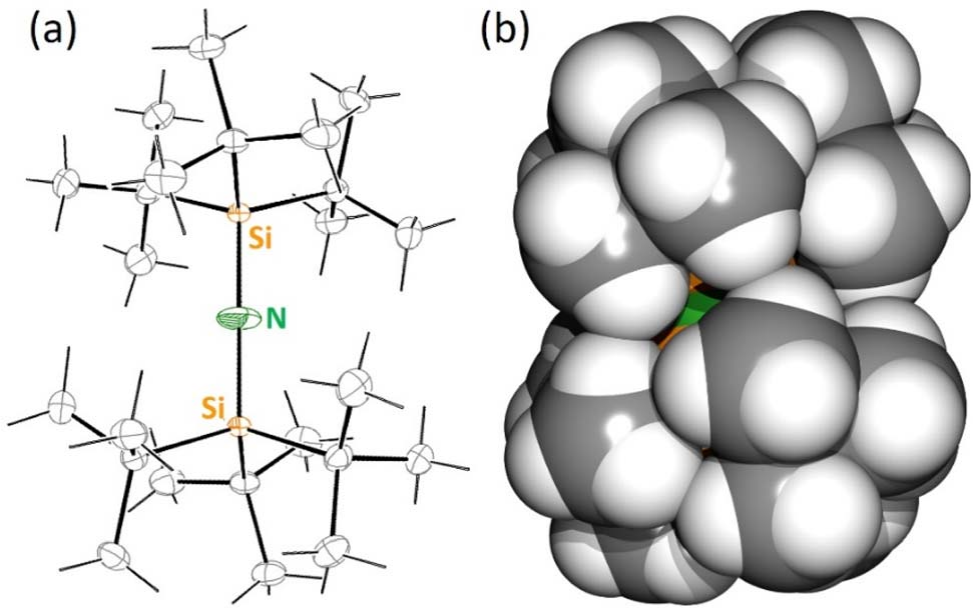
(a) ORTEP representation for the crystal structure of (*t*Bu_3_Si)_2_NH (^*t*Bu^NH). The H atom at N is disordered and was not located. (b) Space-filling model for the crystal structure of (*t*Bu_3_Si)_2_NH (^*t*Bu^NH).

The parent amine (*t*Bu_3_Si)_2_NH (^*t*Bu^NH) was obtained by thermal decomposition of (*t*Bu_3_Si)N=N–NH(Si*t*Bu_3_) following a method reported by Wiberg (Scheme 2).^25^ A slightly modified procedure gave the ligand precursor in crystalline purity (yield: 71%). Although the Si–N–Si angle in ^*t*Bu^NH is reported to be 167(2)°,^25^ a later refinement^26^ and our structure determination show a linear structure with N on an inversion center. There are, however, large N displacement factors in two dimensions (*U*_1_ 0.072, *U*_2_ 0.018, *U*_3_ 0.054) which is the plane perpendicular on the Si–N–Si axis (Fig. 1a). This shows that, similar to [(Ar)_5_Cp]_2_Ae sandwich complexes,^27^ the molecule slightly deviates from linearity and the central N atom is disordered over a ring of positions. Due to disorder the exact location of the N–H hydrogen atom could not be determined. As the N–H functionality is fully embedded in the bulk of two very large *t*Bu_3_Si-substituents (Fig. 1b), the deprotonation of ^*t*Bu^NH turned out to be extremely challenging.

Lithiation with *n*BuLi in boiling hexane, or with *n*BuLi/TMEDA at somewhat lower temperatures to avoid TMEDA deprotonation,^28^ did not give any conversion. Treating ^*t*Bu^NH with KH in boiling THF or under microwave conditions at 180 °C did not show reaction. In contrast, the somewhat smaller amine ^iPr^NH could be smoothly deprotonated even in toluene.^18^ Addition of 18-crown-6 did give deprotonation and a small batch of crystals with composition [K^+^·(18-crown-6)(THF)_2_][^*t*Bu^N^−^] (1) was isolated in very low yields (crystal structure: Fig. S37 and S38†). Repeated attempts to improve this synthesis led to the conclusion that this procedure is irreproducible. As ^*t*Bu^NH did not even react with the superbase mixture *n*BuLi/KO*t*Bu, this amine is particularly resistant towards deprotonation.

**Scheme 3:**
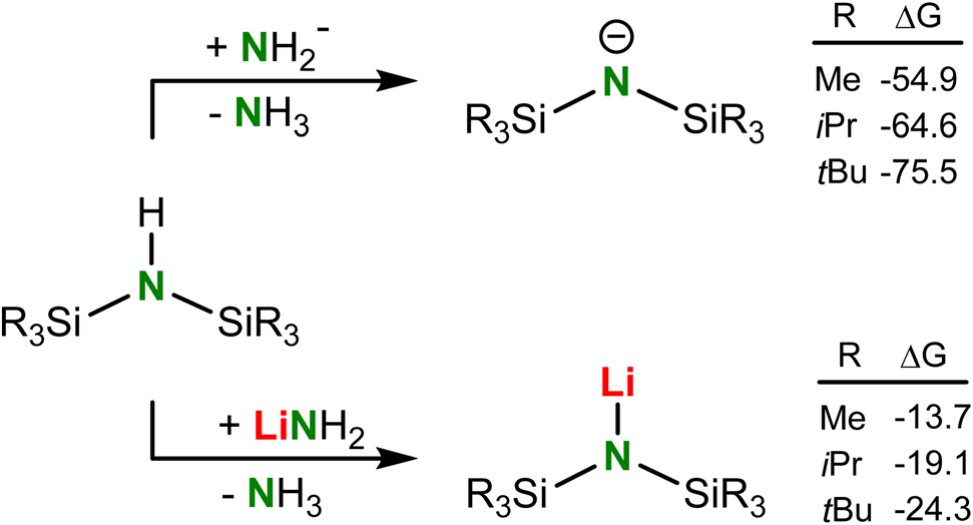
Calculated free energies (298 K, kcal mol^−1^) for the deprotonation of HN(SiR_3_)_2_ with NH_2_^−^ or LiNH_2_ (B3PW91/def2tzvp including GD3BJ dispersion correction).

Analysis of the thermodynamics of the reaction by DFT calculation (B3PW91/def2tzvp including GD3BJ dispersion corrections) showed that silyl-substituents have a strong stabilizing effect on the amide anion and that deprotonation of silyl-substituted amines becomes more exergonic with increasing bulk (Scheme 3). The reluctance of ^*t*Bu^NH to be deprotonated is therefore exclusively due to kinetic problems related to the very poor accessibility of the N–H proton. We reasoned that application of a metal electride^29^ may solve this problem. Addition of ^*t*Bu^NH to a dark-blue solution of K^0^ in NH_3_ led to rapid decolorization, however, we were only able to isolate highly insoluble KNH_2_ in the form of a grey powder. The latter is likely formed by reaction of intermediate (^*t*Bu^N)K with NH_3_ which, although contrathermodynamic (see Scheme 3), can be explained by the insolubility and precipitation of KNH_2_. Reaction of ^*t*Bu^NH with a blue K^0^/18-crown-6 electride solution^29*a*^ led to crown ether decomposition, which is a known side-reaction for such reagents.^30^ However, the reaction with a blue Cs^+^/e^−^ electride formed by addition of Cs^0^ to THF gave full conversion of the amine. The reaction is extremely slow and needs slight heating at 40 °C for four days. Under these conditions not only (^*t*Bu^N)Cs (2) but also several solvent decomposition side-products are formed. As THF is prone to C–H activation in the OCH_2_ group and subsequent ring opening,^31^ we changed the solvent to the more robust tetrahydropyran (THP). Reaction of a Cs^0^/THP electride solution with ^*t*Bu^NH resulted in considerably less side-product formation and gave solvent-free (^*t*Bu^N)Cs in 84% yield. Although formally a deprotonation, this procedure should be described as a redox reaction in which one equivalent of Cs^0^ reduces ^*t*Bu^NH to ^*t*Bu^N^−^ and 0.5 equivalent of H_2_ (the latter could be detected by ^1^H NMR monitoring). We used excess of Cs^0^ to accelerate conversion and reduce the amount of side-products. This synthetic method shows that electrides, which nowadays can also be obtained simply by ball-milling,^29*c*^ may have strong potential as a reagent for the formal deprotonation of challenging substrates.

The amide complex (^*t*Bu^N)Cs (1) with a large heavy alkali metal cation could potentially be used in syntheses of Ae amide complexes by salt-metathesis. However, reactions between (^*t*Bu^N)Cs and AeI_2_ to give (^*t*Bu^N)_2_Ae and CsI were found to be problematic. These ligand exchange reactions in either THF or toluene are very slow, irreproducible and incomplete. This led to complex reaction mixtures from which in one case we were able to isolate some crystals of the ion-pair [ISr^+^·(THF)_6_][^*t*Bu^N^−^] (3) for which we could determine the structure (Fig. S43 and S44†). The reaction of (^*t*Bu^N)Cs with (BDI)MgI in THF was clean and reproducible and the complex [(BDI)Mg^+^·(THF)_2_][^*t*Bu^N^−^] (4) was isolated in 55% yield (BDI = β-diketiminate ligand HC[C(Me)N-DIPP]_2_, DIPP = 2,6-diisopropylphenyl). Product crystallization was problematic but recrystallization from Et_2_O gave good quality crystals of the ion-pair [(BDI)Mg^+^·(THF)(Et_2_O)][^*t*Bu^N^−^] (5) with Et_2_O/THF disorder (Fig. S45 and S46†).

Interestingly, ball-milling (^*t*Bu^N)Cs and LiI and subsequent extraction with hexane cleanly led to formation of (^*t*Bu^N)Li (6) which was isolated in the form of colorless crystals in 55% yield. The driving force for this exchange reaction is the formation of CsI. Note that (^*t*Bu^N)Li could not be obtained directly from the amine and a Li base. Addition of either 12-crown-4 or PMDTA to a THF solution of (^*t*Bu^N)Li led to crystallization of the free ^*t*Bu^N^−^ anion with non-coordinating Li^+^·(12-crown-4)_2_ (7, 71% yield) or Li^+^·(PMDTA)(THF) (8) cations (crystal structures: Fig. S49–S52†); PMDTA = *N*,*N*′,*N*′,*N*′′,*N*′′-pentamethyl-diethylenetriamine.

### Crystal structures of Li and Cs amide complexes

**Fig. 2:**
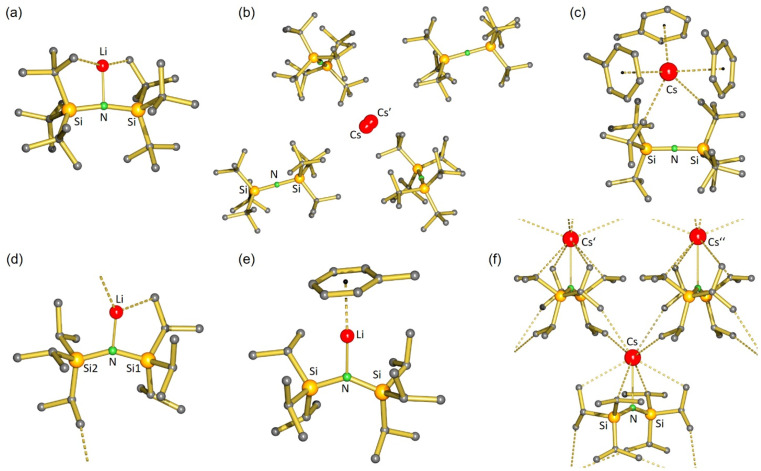
Crystal structures of (a) (^*t*Bu^N)Li (6), (b) (^*t*Bu^N)Cs (2), (c) (^*t*Bu^N)Cs·(toluene)_3_ (2·toluene_3_), (d) (^iPr^N)Li (9), (e) (^iPr^N)Li·(toluene) (9·toluene), and (f) (^iPr^N)Cs (10). In all cases, H atoms have been omitted for clarity.

As it is questionable whether a bulky amide anion like ^*t*Bu^N^−^ can coordinate at all to metal cations, we aimed to reveal the crystal structures of (^*t*Bu^N)Li and (^*t*Bu^N)Cs, preferably without strongly coordinating solvents. Solvent-free (^*t*Bu^N)Li crystallized from hot hexane in space group *P*1 with four independent, partially disordered, structurally similar molecules in the unit cell (Fig. 2a). To date, it represents the only example of a monomeric LiNR_2_ complex without stabilizing interactions between Li^+^ and electron-rich ligands or substituents (*e.g.* O, N, P, aryl). Although the Si–N–Si units with an average angle of 167.4° (range: 165.7(2)°–168.8(2)°) are close to linear, there are distinct N–Li contacts which are surprisingly small (average: 1.913 Å, range: 1.905(7)–1.920(6) Å). For comparison, the N–Li distance of 1.965(4) Å in monomeric (^Me^N)Li·(12-crown-4) is considerably larger.^32^ The tricoordinate N atom has an unusual nearly T-shaped coordination geometry. The Li^+^ cation in (^*t*Bu^N)Li is embedded in a surrounding of *t*Bu groups with extremely short anagostic interactions. The shortest Li⋯CH_3_ distances in the non-disordered molecules range from 2.303(8) to 2.338(7) Å (average: 2.325 Å). This is considerably shorter than the shortest reported anagostic Li⋯CH_3_ interactions for low-coordinate Li complexes by Snaith (2.415(7)–2.823(7) Å; average: 2.607 Å) or Scherer (2.658(5) Å).^33,34^ The shortest Li⋯H distances in a structure determination of (^*t*Bu^N)Li with freely refined H atoms vary from 1.68–2.00 Å but without neutron diffraction data, these values are not accurate.

The very short Li⋯CH_3_ distances in (^*t*Bu^N)Li indicate strong secondary bonding interactions. Since metal⋯H_3_C–X bonds become stronger with decreasing electronegativity of X, *i.e.* with increasing H_3_C^*δ*−^–X^*δ*+^ bond polarity,^35^ it is surprising that the Li⋯H_3_C–C bonds in (^*t*Bu^N)Li (average: 2.325 Å) are so much shorter than the average Li⋯H_3_C^*δ*−^–Si^*δ*+^ anagostic interactions in trimeric^36,37^ or tetrameric^38^ (^Me^N)Li of 2.888 Å and 2.955 Å, respectively. The N–Li bond in (^*t*Bu^N)Li can be easily cleaved by addition of 12-crown-4 which resulted in crystallization of the ion-pair [Li^+^·(12-crown4)_2_][^*t*Bu^N^−^] (7) (see Fig. S49 and S50†).

The amide complex with the larger Cs^+^ cation, (^*t*Bu^N)Cs, is hardly soluble in hexane. Despite numerous attempts, it was impossible to obtain crystals from strictly nonpolar solvents. However, it does dissolve in polar but weakly coordinating chlorobenzene^39^ from which it crystallized solvent-free. The crystal structure (Fig. 2b) shows a nearly linear ^*t*Bu^N^−^ anion (Si–N–Si 177.6(1)°) and very short Si–N bonds (1.652(2) Å). In contrast to (^*t*Bu^N)Li, there is no N–metal bonding (N⋯Cs 5.520(2) Å). The Cs^+^ cation resides in a cavity spanned by four ^*t*Bu^N^−^ anions in which there are at most weak anagostic Cs⋯CH_3_ interactions (3.497(3)–3.562(3) Å). The Cs^+^ atom is disordered over two positions separated by circa 0.45 Å. This is likely due to the extremely weak electrostatic bonding interaction between ^*t*Bu^N^−^, one of the largest amide anions, and Cs^+^, the largest stable metal cation.

Addition of toluene led to strong Cs^+^⋯toluene interactions and crystallization of monomeric (^*t*Bu^N)Cs·(toluene)_3_ in the monoclinic space group *P*2_1_/*c* with one molecule in the asymmetric unit (Fig. 2c). The Cs^+^ cation is bound to the three toluene solvent molecules in a η^6^-fashion with Cs-ring centroid distances ranging from 3.251 to 3.321 Å. The Cs coordination sphere is completed by two anagostic Cs⋯CH_3_ interactions of 3.423(2) and 3.605(2) Å. There is hardly structural information on organometallic Cs compounds. The few reported Cs⋯H_3_C^*δ*−^–Si^*δ*+^ anagostic interactions in (^Me^N)Cs complexes, which should be stronger than Cs⋯H_3_C–C bonds,^35^ are much longer (range: 3.623(4)–3.879(5) Å).^39,40^ This again shows the importance for secondary bonding in metal complexes with the ^*t*Bu^N ligand. The Si–N–Si backbone in the anion ^*t*Bu^N^−^ is close to being linear (177.6(1)°).

Although there are many structures of alkali metal complexes with bulky bis(silyl) amide ligands,^42,43^ a comparison is often difficult due to use of different coordinating solvents or different silyl substituents. In order to compare Li and Cs amide structures with ligands of increasing steric bulk, ^Me^N < ^iPr^N < ^*t*Bu^N, we therefore also synthesized (^iPr^N)Li (9, 85% yield) and (^iPr^N)Cs (10, 68% yield). Both could be crystallized either from apolar hexanes or slightly polar aromatic solvents like toluene.

Solvent-free (^iPr^N)Li (9) crystallizes monomeric with a bent Si–N–Si framework (142.73(7)°) and a very short anagostic Li⋯CH_3_ interaction of 2.292(3) Å to a neighbouring molecule, resulting in chain-like polymer (Fig. 2d). An intramolecular anagostic Li⋯CH_3_ interaction of 2.531(3) Å results in slight asymmetry (Li–N–Si1 = 104.7(1)°; Li–N–Si2 = 111.7(1)°), typically also found in complexes with the ^Me^N-ligand.^44^ The Li–N bond (1.872(3) Å) is significantly shorter than in solvent-free (^*t*Bu^N)Li (average: 1.913 Å). This is due to bending of the amide ligand resulting in compacter orbitals on N with more s-character. When crystallized from toluene, the complex (^iPr^N)Li·(toluene) (9·toluene) was isolated (Fig. 2e). The crystal structure shows capping of the Li^+^ cation with a η^6^-coordinating toluene ligand (Li-centroid: 2.391 Å) leading to disappearance of anagostic Li⋯CH_3_ interactions (Li⋯C > 3.0 Å) and significant elongation of the N–Li bond from 1.872(3) Å in (^iPr^N)Li to 1.916(3) Å in (^iPr^N)Li·(toluene).

Complex (^iPr^N)Cs (10) crystallizes from hexane or hexane/toluene mixtures as a solvent-free *C*_2_-symmetric monomer (Fig. 2f). The structure is bent (Si–N–Si 146.6(1)°) allowing for a Cs–N interaction of 3.017(2) Å which is considerably shorter than those in dimeric [(^Me^N)Cs]_2_ structures (3.016(2)–3.149(2) Å).^45^ Although crystallized in the presence of toluene, there is no aromatic capping ligand, resulting in a two-dimensional network of four intramolecular and four intermolecular anagostic Cs⋯C interactions in the range 3.573(2)–3.710(2) Å.

**Scheme 4:**
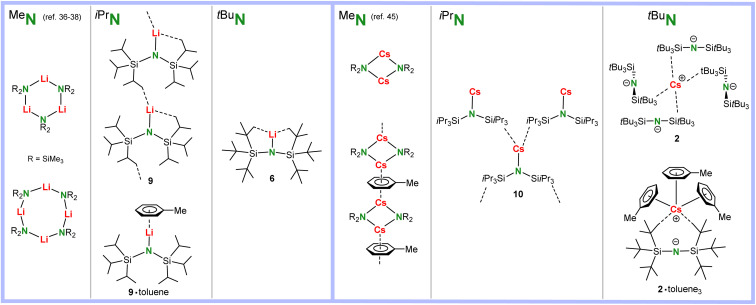
Comparison of polar solvent-free Li and Cs amide structures with ligands of increasing bulk: ^Me^N < ^iPr^N < ^*t*Bu^N.

There is a clear trend in the crystal structures of solvent-free (^Me^N)Li, (^iPr^N)Li and (^*t*Bu^N)Li (Scheme 4). The smallest lithium amide reagent crystallizes either as cyclic trimer or tetramer.^36–38^ The bulkier (^iPr^N)Li crystallizes as a monomer with strong anagostic interactions to neighbours resulting in a one-dimensional polymer. Addition of toluene results in formation of a solvated monomer. The bulkiest (^*t*Bu^N)Li forms a discrete monomer in which Li is saturated only by intramolecular anagostic interactions. Although the N–Li bond in (^*t*Bu^N)Li is easily broken by addition of 12-crown-4, crystallization of (^Me^N)Li with this Li^+^ specific crown ether gave monomeric (^Me^N)Li·(12-crown-4) with a short N–Li contact of 1.965(4) Å.^32^

Although alkali metal complexes with large cations usually tend to form extensive coordination polymers, the structure of solvent-free (^Me^N)Cs is only dimeric, while addition of toluene results in a linear array of dimers bridged by Cs⋯η^6^-toluene interactions (Scheme 4).^45^ In contrast, (^iPr^N)Cs crystallizes from hexanes as a monomer with a short Cs–N distance and an extended network of intra- and intermolecular anagostic interactions. The complex with the bulkiest amide ligand, (^*t*Bu^N)Cs, crystallized as an ion-pair in which the amide ligand functions as a weakly coordinating anion (WCA) through longer anagostic Cs⋯C interactions. From toluene the complex crystallized as (^*t*Bu^N)Cs·(toluene)_3_ in which there is also no Cs–N contact.

The superbulky amide anion ^*t*Bu^N^−^ is therefore an odd example of a halogen-free WCA. Nearly all WCAs are heavily fluorinated or halogenated^46,47^ and there are only few examples of halogen-free WCAs.^48,49^ The latter are especially desirable for their great stability as electrolytes in metal batteries.^50,51^

### Theory

**Scheme 5:**
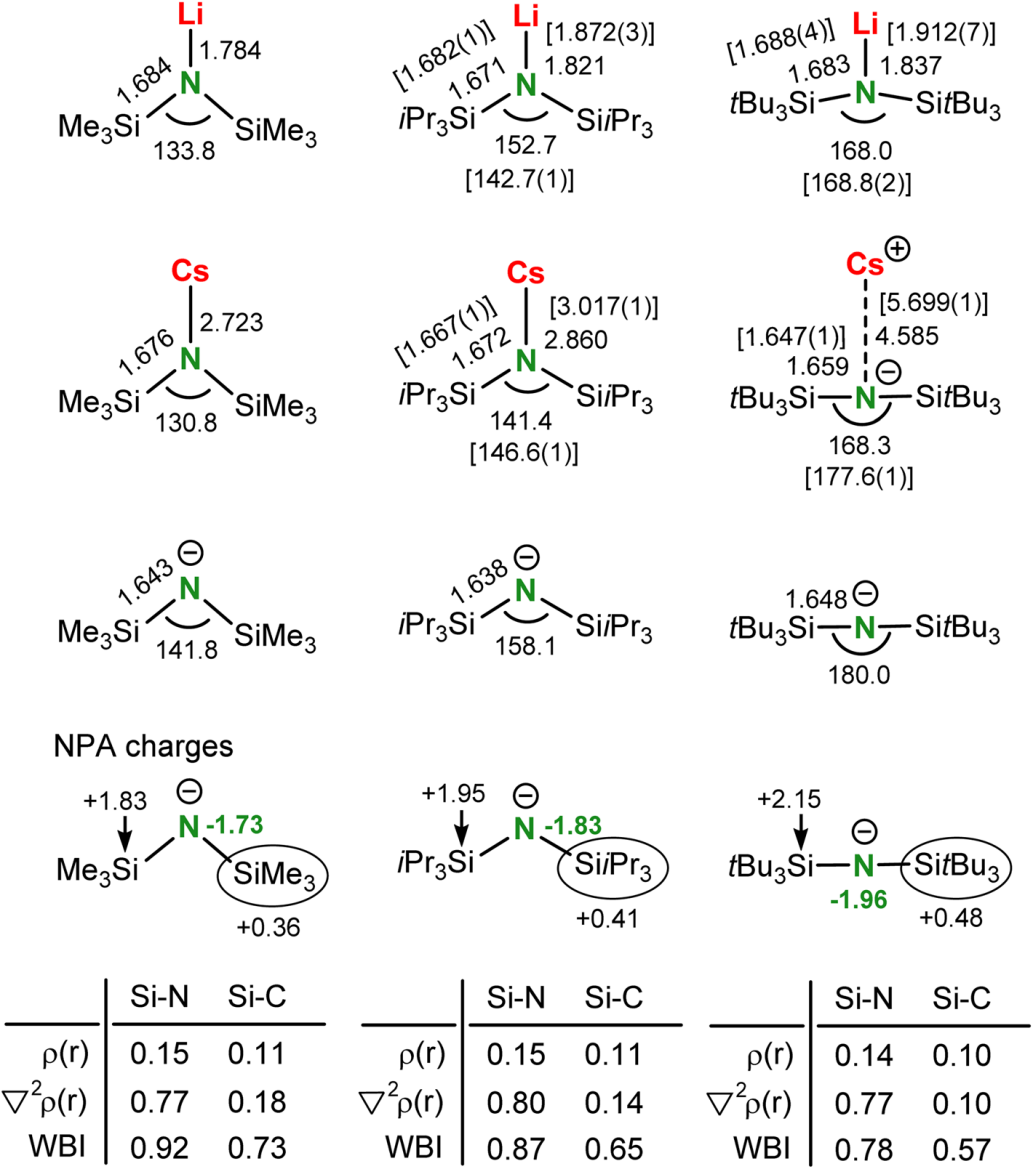
Comparison of DFT-optimized Li and Cs amide complexes and the free anions ^Me^N^−^, ^iPr^N^−^ and ^*t*Bu^N^−^ (B3PW91/def2tzvp including GD3BJ dispersion corrections) showing distances (Å), angles (°), Wiberg bond indices (WBIs) and values for *ρ*(***r***) and the Laplacian ∇^2^*ρ*(***r***) in the bond-critical point (a.u.). Values for crystal structures are given between square brackets.

There is a strong analogy between the metal coordination of the bis(silyl) amide anion ^*t*Bu^N^−^ and that of the isolobal bis(silyl) ethers R_3_SiOSiR_3_ which are notorious for their extremely poor donor ability.^52–55^ This is underscored by the fact that hydrogen bonds to silyl ethers are very rare.^56–58^ A first example of unsupported metal⋯O(SiMe_3_)_2_ bonding was only recently structurally characterized.^59^ It has been previously discussed that the main reason for the poor electron pair donating abilities of R_3_SiOSiR_3_ is negative hyperconjugation, *i.e.* delocalization of free electron pairs at the central O into the σ*(Si–R) bond, leading to wide Si–O–Si angles, short Si–O bonds and long Si–R bonds.^54^ However, this theory has been abandoned and its unusual geometry can also be explained with the ionic character of the Si–O bond which increases upon widening the Si–O–Si angle.^52,55^ Despite the strong similarities between isolobal (R_3_Si)_2_O and (R_3_Si)_2_N^−^ species, the bonding and electronic structure of bis(silyl) amide ligands has not been described in detail. Herein we provide DFT calculations on monomeric Li and Cs complexes and free anions of increasing bulk: ^Me^N^−^ < ^iPr^N^−^ < ^*t*Bu^N^−^ (Scheme 5).

The structures have been optimized at the B3PW91/def2tzvp level of theory. As it was found that using Grimme's third dispersion correction with Becke–Johnson damping (GD3BJ) gave a better match with the experimental data, we only show results with dispersion correction. The coordination geometry of Li in (^*t*Bu^N)Li deserves some special attention. Also in the calculated structure the Li^+^ cation is fully embedded in the ligand and bound by a short N–Li bond and anagostic interactions. This results in an unusually large value for the buried volume (*V*_bur_).^60^ For monomeric structures the following values have been calculated: (^Me^N)Li 45.4%, (^iPr^N)Li 72.7% and (^*t*Bu^N)Li 86.6% (H atoms have been included, Table S2 and Fig. S32–S34†).

Comparison of the optimized structures for (^Me^N)Li, (^iPr^N)Li and (^*t*Bu^N)Li shows that the N–Li bonds slightly elongate and the Si–N–Si angles considerably widen when the ligand bulk is increased. A similar trend can be recognized for the corresponding Cs amide complexes with the difference that the Cs–N bond in (^*t*Bu^N)Cs is completely cleaved, even in a calculated gas phase structure. The difference between The Si–N bond distances remain surprisingly constant when increasing the bulk of the silyl substituents.

As Li and Cs are extremes in the alkali metal series, we also calculated the structures of (^*t*Bu^N)M (M = Na, K, Rb; Fig. S24†). The gradual increase in calculated N–M distances is larger than the increase in ionic radii (Table 1). The difference between these values, (N–M) – (ionic radius), steadily increases from Li to Rb and at Cs becomes extremely large. The calculated N–metal distances in (^*t*Bu^N)M compare well with those in crystal structures of monomeric (^Me^N)M complexes in which metals have been solvated with multi-dentate ligands (Table 1). These data show that although the ^*t*Bu^N^−^ anion becomes gradually less coordinating from Li^+^ to Rb^+^, it is truly weakly coordinating only for Cs^+^. However, it should be considered that these are gas phase calculations in which charge separation is notoriously difficult. Even weak donor ligands like aromatic solvents may induce N–M bond dissociation already for smaller metal cations.

**Table 1:** Comparison of the calculated N–M distances (Å) in (^*t*Bu^N)M complexes (M = Li–Cs) with ionic radii for six-coordinate metal cations and N–M distances in monomeric (^Me^N)M complexes with multidentate ligands^a^

M	Li	Na	K	Rb	Cs
N–M (calcd)	1.837	2.246	2.690	3.001	4.585
Ionic radii (CN = 6)	0.76	1.02	1.38	1.52	1.67
(N–M) – (ionic radius)	1.077	1.226	1.310	1.481	2.915
N–M in (^Me^N)M^a^	1.965	2.306	2.760	3.038	3.086

a Experimental values in monomeric (^Me^N)M complexes with multidentate ligands; Li: 12-crown-4, Na: (dimethoxyethane)_2_, K and Rb: 18-crown-6, Cs: N(CH_2_CH_2_OCH_2_CH_2_OMe)_3_.^68^

Comparison of the free amide anions show a similar widening of Si–N–Si angles. Optimization of the ^Me^N^−^ anion without considering dispersion gave a linear minimum with a Si–N–Si angle of 179.8°. However, with correction for dispersion it optimized to a bent structure with a Si–N–Si angle of 141.8° which fits better to experimental values for free ^Me^N^−^ anions (128.6(1)–143.2(1)°).^61–63^ The value for the ^iPr^N^−^ anion (158.1°) also corresponds with experiment (152.8(1)°).^64^ The calculated Si–N–Si angle in ^*t*Bu^N^−^ is truly linear (180.0) and fits the angle in the crystal structure of (^*t*Bu^N)Cs (177.6(1)°) and those in structures with free amide anions [Li^+^·(PMDTA)(THF)][^*t*Bu^N^−^], [Li^+^·(12-crown-4)_2_][^*t*Bu^N^−^], [ISr^+^·(THF)_6_][^*t*Bu^N^−^] and [(BDI)Mg^+^·(THF)_*x*_][^*t*Bu^N^−^] in which the Si–N–Si angles range from 173.2(1)° to 179.3(1)°. Interestingly, also in the free anions the calculated Si–N distances hardly vary (range: 1.638–1.648 Å). This stands in stark contrast with the observed trend that the Si–O bonds in H_3_Si–O–SiH_3_ become shorter and more ionic when widening the Si–O–Si angle.^52^ It also contradicts with a strong decrease of Si–O bond lengths in R_3_Si–O–CR′_3_ upon becoming more linear.^65^ The invariance in Si–N bond lengths in ^Me^N^−^, ^iPr^N^−^ and ^*t*Bu^N^−^ is likely due to two opposing effects that counterbalance each other: (a) increasing bulk results in Si–N–Si widening and therefore Si–N bond shortening, (b) increasing bulk results in Si–N bond lengthening due to increased repulsion of the silyl substituents. In contrast to the invariance of the Si–N bond lengths, the Si–C bonds become longer with widening the Si–N–Si angle. This effect is amplified by increased repulsion of the silyl substituents. Lengthening of the Si–C bonds is in agreement with decreasing Wiberg bond indices (WBIs) (Scheme 5).

Although the Si–N bonds in the series are of similar length, the WBIs are reduced going from ^Me^N^−^ (0.92) to ^*t*Bu^N^−^ (0.78) due to an increase of the ionic character in the Si–N bond. The increase of Si–N bond ionicity in the row ^Me^N^−^ < ^iPr^N^−^ < ^*t*Bu^N^−^ is evident from the charges calculated by Natural-Population-Analysis (Scheme 5).^66^ Bulky substituents result in Si–N–Si widening and an increase of negative charge on N from −1.73 (^Me^N^−^) to −1.96 (^*t*Bu^N^−^) with a concomitant increase of positive charge from +1.83 to +2.15 on Si. The ionicity of the Si–N bonds is also evident from atoms-in-molecules analysis.^67^ Covalent bonds typically have large electron densities *ρ*(***r***) and negative values for the Laplacian ∇^2^*ρ*(***r***) in the bond-critical-point (bcp). The Si–N bonds in the series have low electron densities (0.14–0.15 a.u.) and positive Laplacians (0.77–0.80 a.u.), typically observed for ionic bonding (Scheme 5).

It seems counterintuitive that the amide anion with the highest charge on N (^*t*Bu^N^−^) shows the poorest donor ability. Although this partially may be explained by steric hindrance, poor coordination properties have also been described for linear H_3_Si–O–SiH_3_ in which sterics do not play any role.^55^ A simple electronic explanation can be found in differences in the spatial arrangement of electrons at the donor site. Whereas the electron density at the central O in bent ether ligands is directional and has the form of a cashew nut, the electron pairs in a linear ether are in a circular donut shape.^55^ Despite the high electron density at O in the latter, there is an unfavorable non-directional distribution of the charge density. Similar arguments explain the weakly coordinating behavior of the nearly linear ^*t*Bu^N^−^ anion.

**Fig. 3:**
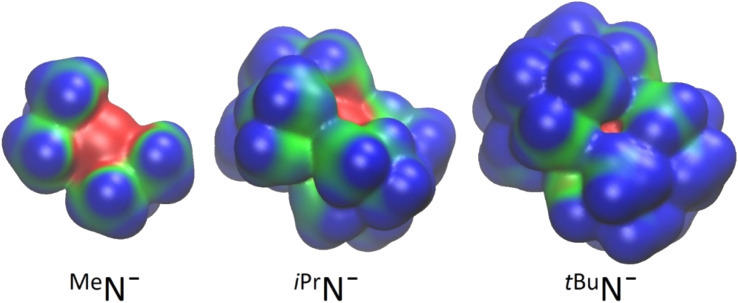
Electrostatic potential isosurfaces for bis(silyl) amide anions. Red is negatively charged, blue is positively charged.

The weakly coordinating behavior of ^*t*Bu^N^−^ is also nicely demonstrated by comparison of its electrostatic potential isosurface with those of ^iPr^N^−^ and ^Me^N^−^ (Fig. 3). The negatively charged N (in red) in ^*t*Bu^N^−^ is completely buried by ligand bulk and the positively charged surface (in blue) is highly unfavorable for interactions with cations.

## Conclusion

The superbulky amine (*t*Bu_3_Si)_2_NH (^*t*Bu^NH) can be easily obtained by a synthetic method reported by Wiberg and coworkers. However, deprotonation of the relatively acidic N–H functionality turned out to be extremely difficult and could not even be achieved with *n*BuLi/KO*t*Bu superbase mixtures. This stands in complete contrast to the facile deprotonation of the somewhat smaller amine ligand (iPr_3_Si)_2_NH (^iPr^NH) which reacts smoothly with *n*BuLi or KH.^18,42^ The origin for its reluctance to be deprotonated lies in steric congestion and poor accessibility. However, using a blue electride solution of Cs^+^/e^−^ in THF resulted in slow deprotonation and formation of (^*t*Bu^N)Cs. This reagent is also the key to (^*t*Bu^N)Li which could be obtained by reaction of (^*t*Bu^N)Cs with LiI. However, in contrast to the facile salt metathesis reactions with (^iPr^N)K,^20,43^ the application of (^*t*Bu^N)Cs in the synthesis of other metal complexes is limited.

These solvent-free superbulky amide complexes could be obtained in crystalline form by recrystallization from either apolar hexanes or weakly coordinating polar solvents like chlorobenzene. Although the N atom in the anion ^*t*Bu^N^−^ is completely shielded by large bulky *t*Bu_3_Si-substituents, small cations like Li^+^ can be embedded between substituents and form relatively short N–Li bonds (1.913 Å). This only results in very slight bending of the Si–N–Si backbone (167.4°) and therefore an unusual T-shaped coordination geometry around N. Replacing Li^+^ for the much larger Cs^+^ cation led to cleavage of the metal–N bond and formation of an ion-pair, even in the absence of stabilizing solvent molecules.

Structural comparison of a range of Li and Cs amide complexes with ligands of increasing bulk (^Me^N < ^iPr^N < ^*t*Bu^N) shows that the ^*t*Bu^N^−^ anion can be considered a WCA, at least for large cations like Cs^+^ but not for Li^+^. Reduction of the ligand bulk to ^iPr^N already results in N–Cs bonding. The presence of polar solvents like ethers or amines leads to cleavage of the ^*t*Bu^N-metal bond, also for metals like Li^+^, Mg^2+^ or Sr^2+^.

DFT analysis of a series of Li and Cs amide complexes with ligands of increasing bulk support these experimental observations: (^*t*Bu^N)Li optimizes as a contact ion-pair with a short N–Li bond whereas (^*t*Bu^N)Cs optimizes as a separated ion-pair with a long N⋯Cs distance. Calculations also show that increasing the Si–N–Si angle results in more ionic and shorter Si–N bonding. Although the negative charge on N is largest in the bulkiest linear amide anion, ^*t*Bu^N^−^, this is the anion showing the poorest ability to coordinate to metals. This can be explained partially by steric arguments but also finds it origin in the non-directional distribution of electron density along the N atom.

The very poor donor ability of the ^*t*Bu^N^−^ anion can be exploited in the search for new non- or weakly coordinating anions. It is a rare example of a WCA that is free of halogens. We are currently investigating potential applications of ^*t*Bu^N^−^ and similar bis(silyl)amide anions as WCAs.

## Data availability

Crystallographic data has been deposited with the Cambridge structural database.

## Author contributions

C. Knüpfer: conceptualization, investigation, validation, formal analysis, writing – original draft, visualization. L. Klerner: investigation, validation, formal analysis. J. Mai: investigation, validation, formal analysis. J. Langer: formal analysis, validation. Sjoerd Harder: conceptualization, writing – original draft – review and editing, visualization, validation, supervision, project administration.

## Conflicts of interest

There are no conflicts to declare.

## Supplementary Material

SC-015-D3SC06896J-s001

SC-015-D3SC06896J-s002
