# Supplementary material for: s-Block metal complexes of superbulky (tBu3Si)2N−: a new weakly coordinating anion?
Source: Chem Sci. 2024 Feb 20;15(12):4386–95. doi: 10.1039/d3sc06896j (PMC10952107; doi:10.1039/d3sc06896j)
Supplement: SC-015-D3SC06896J-s001 [file SC-015-D3SC06896J-s001.pdf]

Electronic Supplementary Material (ESI) for Chemical Science.
This journal is © The Royal Society of Chemistry 2024

General Experimental Procedures ____ 2

Syntheses ____ 3

^{tBu}NH ____ 3

[K^+ (18-crown-6) $(THF)_2$] [$^{tBu}N^-$] ____ 3

(^{tBu}N)Cs ____ 5

[(BDI)Mg^+ $(THF)_2$] [$^{tBu}N^-$] ____ 7

(^{tBu}N)Li ____ 10

[Li^+ $(12\text{-crown-}4)_2$] [$^{tBu}N^-$] ____ 13

(^{iPr}N)Li ____ 16

(^{iPr}N)Cs ____ 19

Reaction of ^{tBu}NH with K in liquid NH_3 ____ 21

Reaction of ^{tBu}NH with K and 18-crown-6 ____ 21

DFT Calculations ____ 22

Buried Volume Determination & Steric Maps ____ 30

XRD Data ____ 33

XYZ Coordinates ____ 51

References ____ 64

General Experimental Procedures

All experiments were conducted in dry glassware under an inert nitrogen atmosphere by applying standard Schlenk techniques or gloveboxes (MBraun). Deuterated benzene (C_6D_6, 99.6% D, Sigma-Aldrich), toluene (tol-d_8, 99.6% D, Sigma-Aldrich) and THF (THF-d_8, 99.5% D, Sigma-Aldrich) were degassed, dried over activated molecular sieves (3 Å) and stored under an inert atmosphere.

N-pentane, *n*-hexane, methylcyclohexane, THF, diethyl ether, benzene and toluene were degassed with nitrogen and dried over a column with activated aluminium oxide in a Solvent Purification System (Innovative Technology, Pure Solv 400-4-MD) and then stored under inert atmosphere over molecular sieves (3 Å). THP was dried over calcium hydride, vacuum distilled and stored under inert atmosphere over molecular sieves (3 Å).

[(tBu$_3$Si)$_2$N$_3$]H,[1,2] ^{DIPP}BDIMgI[3] and (iPr$_3$Si)$_2$NH[4] were prepared according to literature procedures. The crown ethers 12-crown-4 (98%, abcr) and 18-crown-6 (98%, TCI Chemicals) and the reagent *n*-butyllithium (2.5 M in hexanes, Sigma-Aldrich) were used as received. LiI (98%, abcr) was dried by heating to 150 °C under reduced pressure. KH (30 wt% in mineral oil, Sigma-Aldrich) was filtered, washed with hexane and dried under vacuum. K (98% in mineral oil, abcr) was washed several times with hexanes and dried under vacuum.

Mechanochemical experiments were performed using a Retsch MM400 multipurpose mill in 5 mL stainless steel vessel equipped with 20 stainless steel balls (3 mm).

NMR spectra were measured on Bruker Avance III HD 400 MHz and Bruker Avance III HD 600 MHz NMR spectrometers. Chemical shifts (δ) are denoted in ppm (parts per million) and coupling constants in Hz (Hertz). ^{1}H and ^{13}C NMR spectra were referenced to the solvent residual signal ($SiMe_4$ = 0 ppm). Signal multiplicities are described using common abbreviations: s (singlet), d (doublet), t (triplet), q (quartet), quint (quintet), m (multiplet) and br (broad). CHN elemental analysis was performed with a Hekatech Eurovector EA3000 analyzer. Note: Products with superbulky (tBu$_3$Si)$_2$N$^-$ amide anions are generally very well soluble and also hard to crystallize. Therefore, purification by crystallization is often difficult or impossible. For this reason, it was extremely challenging to obtain fitting CHN analyses.

Crystal structures have been measured on a SuperNova (Agilent) diffractometer with dual Cu and Mo microfocus sources and an Atlas S2 detector. The crystal structure data have been deposited with the Cambridge Crystallographic Data Centre. CCDC-numbers 2321294-2321305 contain the supplementary crystallographic data for this compound. This data can be obtained free of charge from The Cambridge Crystallographic Data Centre via www.ccdc.cam.ac.uk/data_request/cif.

Syntheses

^{tBu}NH

(tBu$_3$Si)$_2$NH was prepared according to a modified literature procedure.[5]

Sublimated ([tBu$_3$Si)$_2$N$_3$]H (589 mg, 1.33 mmol) was dissolved in toluene (5 mL), sealed in an ampoule or microwave vial (volume: 20 mL) and heated to 180 °C for 16 h. The initially yellow solution turns colourless during the reaction. The reaction mixture was concentrated until incipient crystallization and heated to redissolve the precipitate. Upon cooling, (tBu$_3$Si)$_2$NH crystallized as colourless blocks. Multiple crops can be obtained from the mother liquor (391 mg, 0.94 mmol, 71%). Crystals suitable for X-ray diffraction analysis were obtained by the same method.

Analytical data is in agreement with the literature.[5]

[K^+ (18-crown-6) (THF)$_2$] [$^{tBu}N^-$] (1)

A J. Young NMR tube was charged with (tBu$_3$Si)$_2$NH (104 mg, 0.251 mmol), KH (11 mg, 284 mmol), 18-crown-6 (67 mg, 252 mmol) and THF-d_8 (700 µL). The reaction mixture was heated to 75 °C for 48 h, resulting in *ca.* 50% conversion according to the proton NMR spectrum of the reaction mixture. Adding more KH did not lead to further conversion (Figure S2). After filtration, washing with pentane (2 x 1 mL) and redissolving in THF-d_8 (500 µL), a small crop of diffraction quality crystals was obtained by cooling the solution to –30 °C. This purification and crystallization procedure is not reproducible and numerous attemps did not provide us with a pure sample.

^{1}H NMR (600 MHz, 25 °C, THF-d_8): δ_H = 3.62 (s, 12 H, 18-crown-6), 1.22 (s, residual amine), 1.10 (s, 54 H, –Si(C(CH_3)$_3$) ppm.

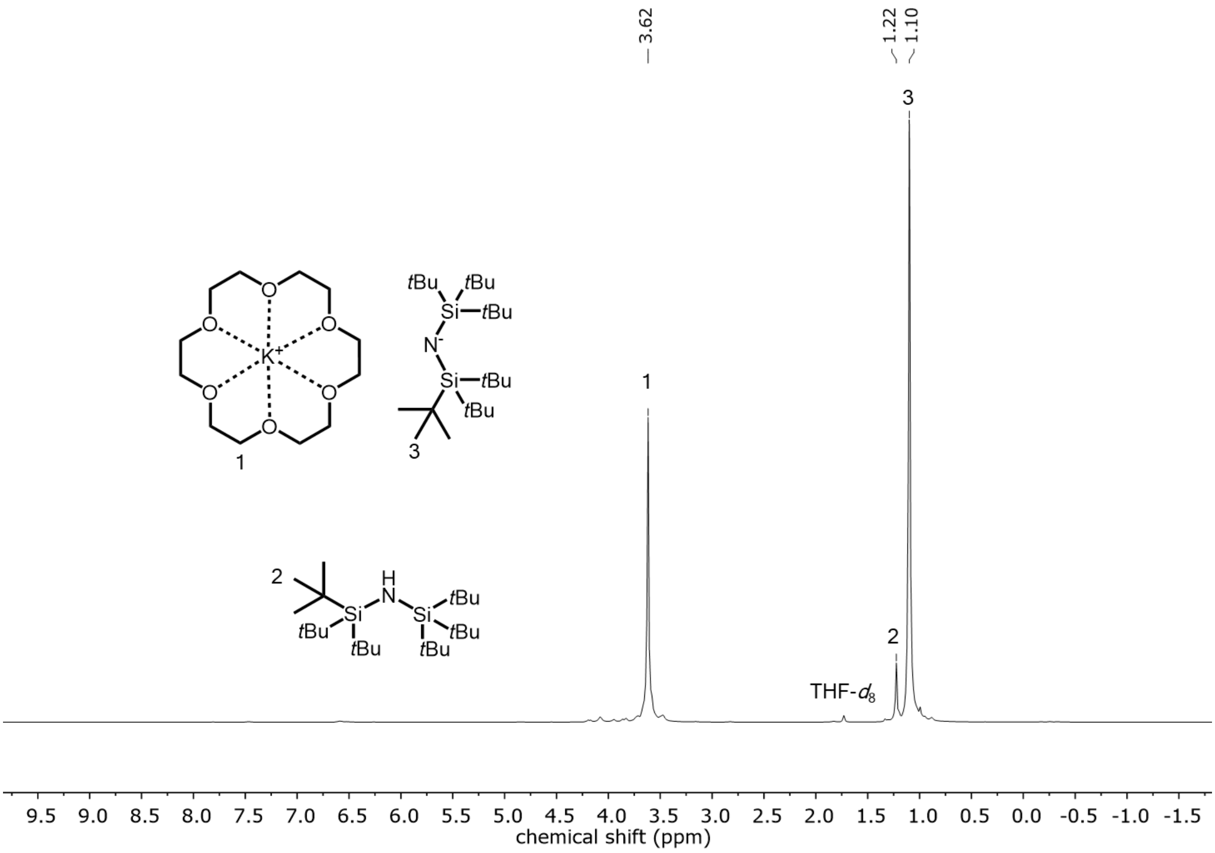

Figure S1: ^{1}H NMR spectrum of [K^{+} (18-crown-6) (THF)$_2$] [^{tBu}N^{-}] in THF-d_8 (600 MHz, 25 °C).

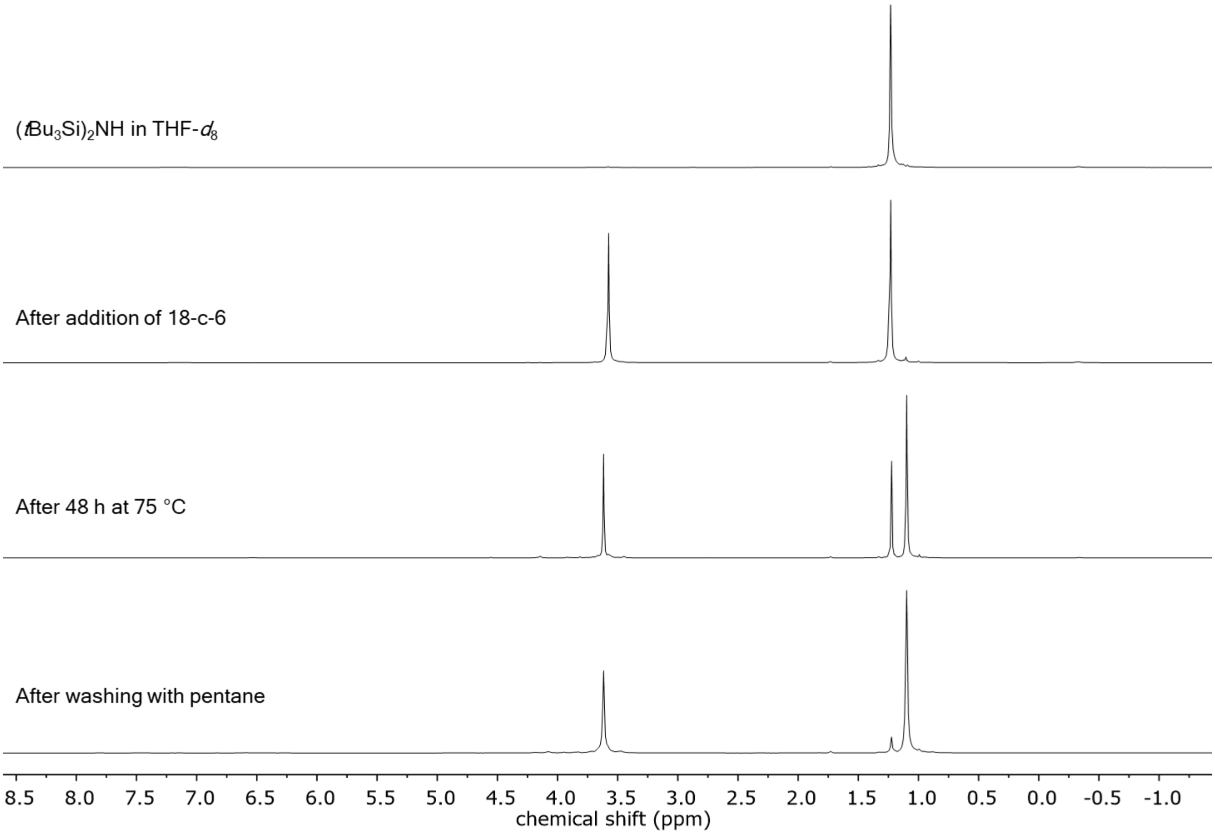

Figure S2: stacked ^{1}H NMR spectra of the reaction between (tBu$_3$Si)$_2$NH and KH in THF-d_8 (600 MHz, 25 °C).

(^{tBu}N)Cs (2)

A Schlenk flask equipped with a glass coated stirring bar was charged with cesium (1.79 g, 13.5 mmol) and THP (40 mL). (tBu$_3$Si)$_2$NH (1.90 g, 4.59 mmol) was added and the mixture heated to 40 °C for 4 d. The brown supernatant solution was decanted and the residue extracted with THP (30 mL). The combined extracts were dried and toluene (20 mL) was added to displace coordinated THP. After drying under reduced pressure, the resulting colourless solid was washed with pentane (4 x 15 mL) to yield (tBu$_3$Si)$_2$NCs (2.11 g, 3.87 mmol, 84%). Crystals suitable for X-ray diffraction analysis were obtained by slowly cooling a saturated solution of the amide in toluene from approximately 70 °C to room temperature.

^{1}H NMR (600 MHz, 25 °C, THF-d_8): δ_H = 1.10 (s, –Si(C(CH_3)$_3$)$_3$ ppm.

^{13}C{^{1}H} NMR (151 MHz, 25 °C, THF-d_8): δ_C = 33.8 (s, CMe_3), 26.9 (s, Si(CMe$_3$)$_3$ ppm.

^{29}Si{^{1}H} NMR (119 MHz, 25 °C, THF-d_8): δ_{Si} = –25.8 ppm.

Elemental analysis: calculated (%) for C24H54CsNSi2 (545.78 g*mol^{-1}): C 52.82, H 9.97, N 2.57; found: C 51.44, H 9.52, N 2.29. Although these results are outside the range viewed as establishing analytical purity, they are provided to illustrate the best values obtained to date.

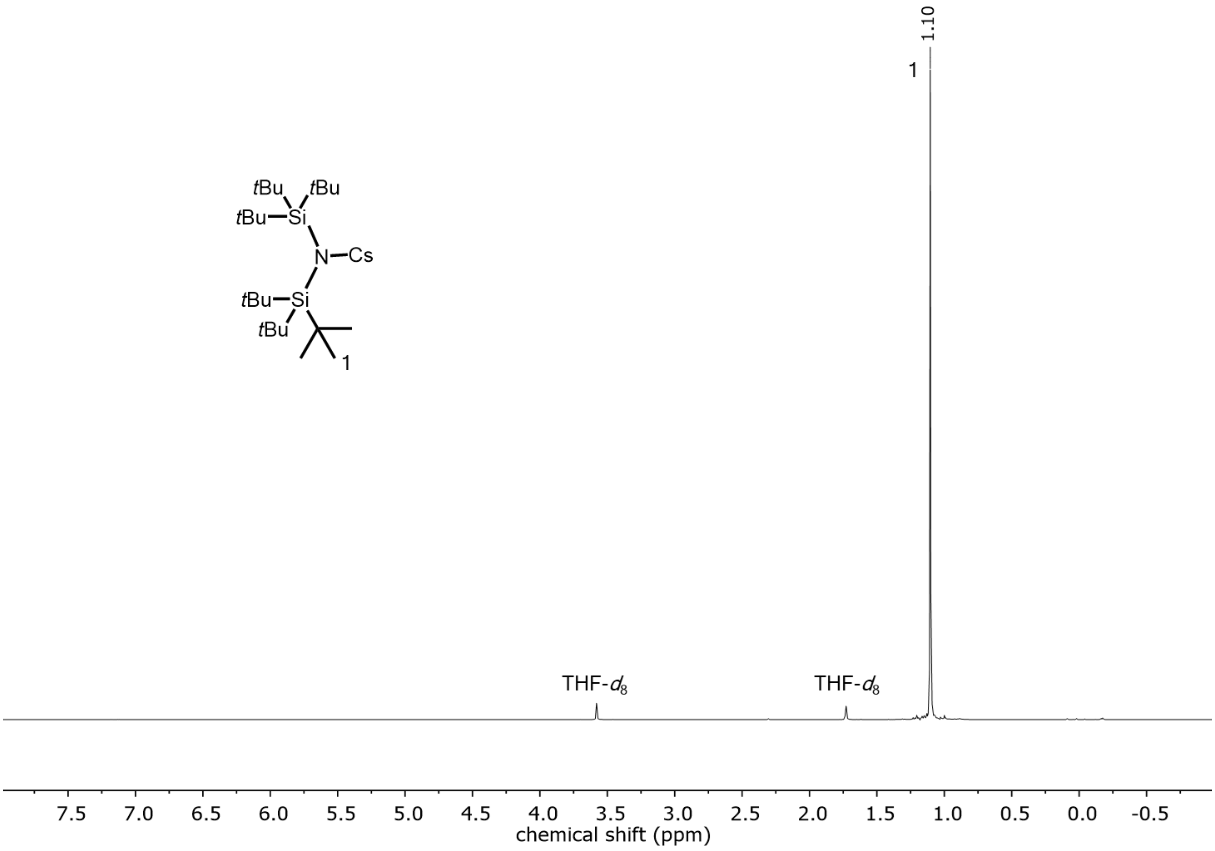

Figure S3: ^{1}H NMR spectrum of (tBu$_3$Si)$_2$NCs in THF-d_8 (600 MHz, 25 °C).

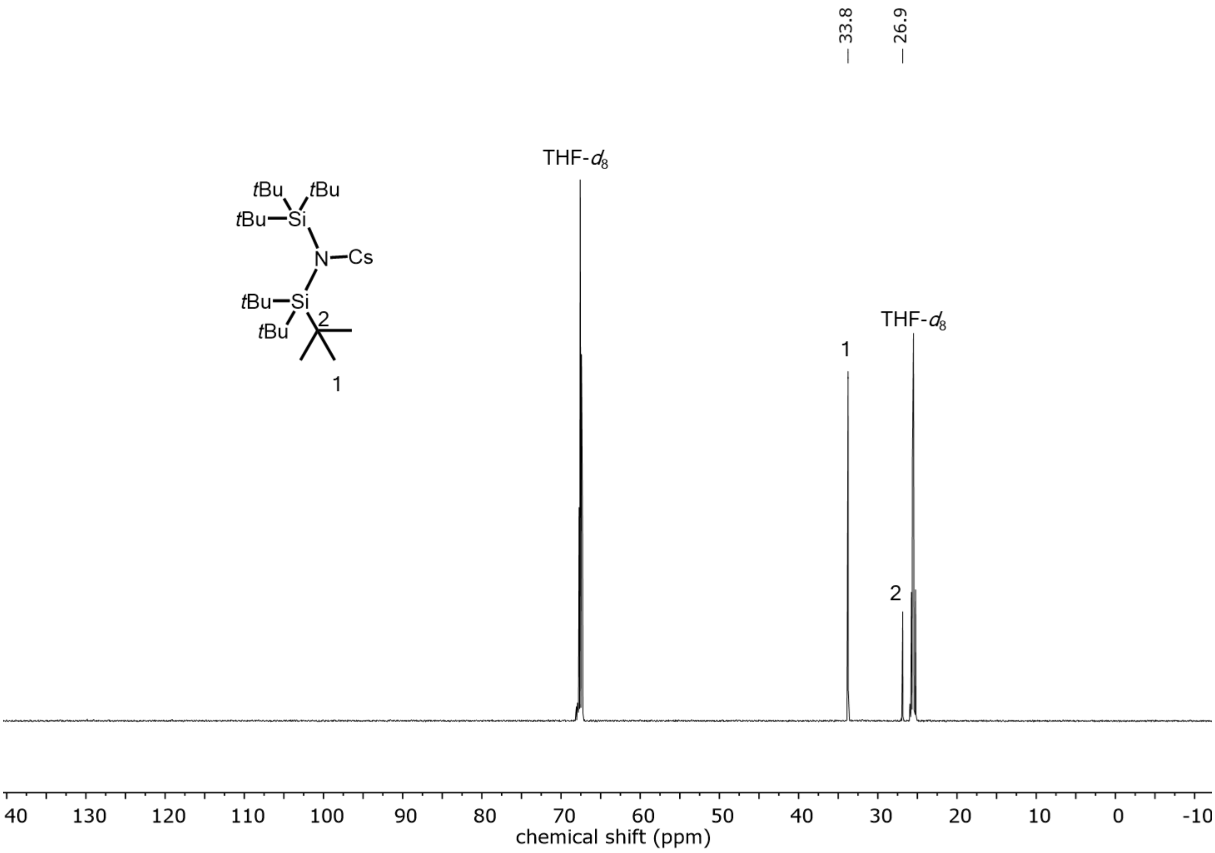

Figure S4: $^{13}C\{^1H\}$ NMR spectrum of (tBu$_3$Si)$_2$NCs in THF-d_8 (151 MHz, 25 °C).

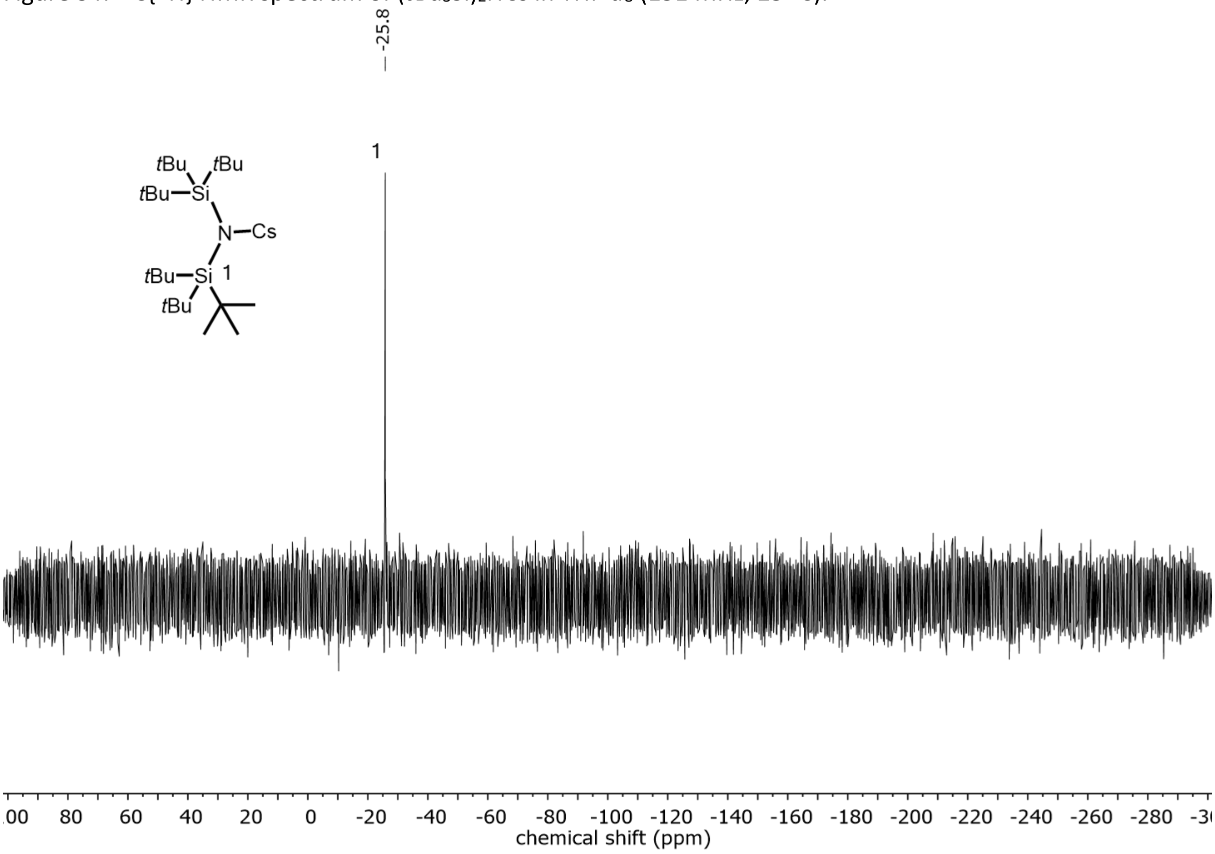

Figure S5: $^{29}Si\{^1H\}$ NMR spectrum of (tBu$_3$Si)$_2$NCs in THF-d_8 (119 MHz, 25 °C).

[(BDI)Mg$^+$ (THF)$_2$] [^{tBu}N$^-$] (4)

A solution of (tBu$_3$Si)$_2$NCs (142 mg, 0.260 mmol) in THF (4 mL) was added to a solution of (^{DIPP}BDI)MgI (151 mg, 0.265 mmol) in THF (5 mL) at room temperature. A colourless precipitate formed immediately and the reaction mixture was stirred for 1 h. The reaction mixture was filtered and dried to yield crude [(BDI)Mg$^+$ (THF)$_2$] [^{tBu}N$^-$] as a colourless solid (142 mg, 0.142 mmol, 55%). Diffraction quality crystals were obtained by cooling a saturated solution of the cationic complex in Et$_2$O from 25 °C to –30 °C. In this case, THF is partially substituted by Et$_2$O, resulting in THF/Et$_2$O disorder.

^{1}H NMR (600 MHz, 25 °C, THF-d_8): δ_H = 7.19–7.16 (m, 6 H, Ar–*H*), 5.04 (s, 1 H, MeCC*H),* 3.63–3.61 (m, 8 H, THF), 3.16–3.11 (m, 4 H, Ar–C*H*Me$_2$), 1.79–1.77 (m, 8 H, THF), 1.73 (s, *Me*CCH, overlapping with solvent signal), 1.25 (d, $^3J_{HH}$ = 6.9 Hz, 12 H, Ar–CH*Me$_2$*), 1.22 (d, $^3J_{HH}$ = 6.9 Hz, 12 H, Ar–CH*Me$_2$*), 1.10 (s, 54 H, –Si(C(C*H$_3$*)$_3$) ppm.

^{13}C{^{1}H} NMR (151 MHz, 25 °C, THF-d_8): δ_C = 143.3 (s, HC*C*Me), 126.6 (s, Aryl), 124.7 (s, Aryl), 124.4 (s, Aryl), 123.6 (s, Aryl), 95.8 (s, H*C*CMe), 68.4 (s, THF), 33.7 (s, –Si(C*Me$_3$*)$_3$), 29.1 (s, Ar–*C*HMe$_2$), 26.9 (s, ArCH*Me$_2$*), 26.5 (s, –Si(*C*Me$_3$)$_3$), 24.7 (s, ArCH*Me$_2$*) ppm.

^{29}Si{^{1}H} NMR (119 MHz, 25 °C, THF-d_8): δ_{Si} = –25.8 ppm.

Elemental analysis: calculated (%) for $C_{61}H_{113}MgN_3O_2Si_2$ (1001.07 g*mol^{-1}): C 73.19, H 11.38, N 4.20; found: C 72.56, H 11.74, N 4.17. Although these results are outside the range viewed as establishing analytical purity, they are provided to illustrate the best values obtained to date.

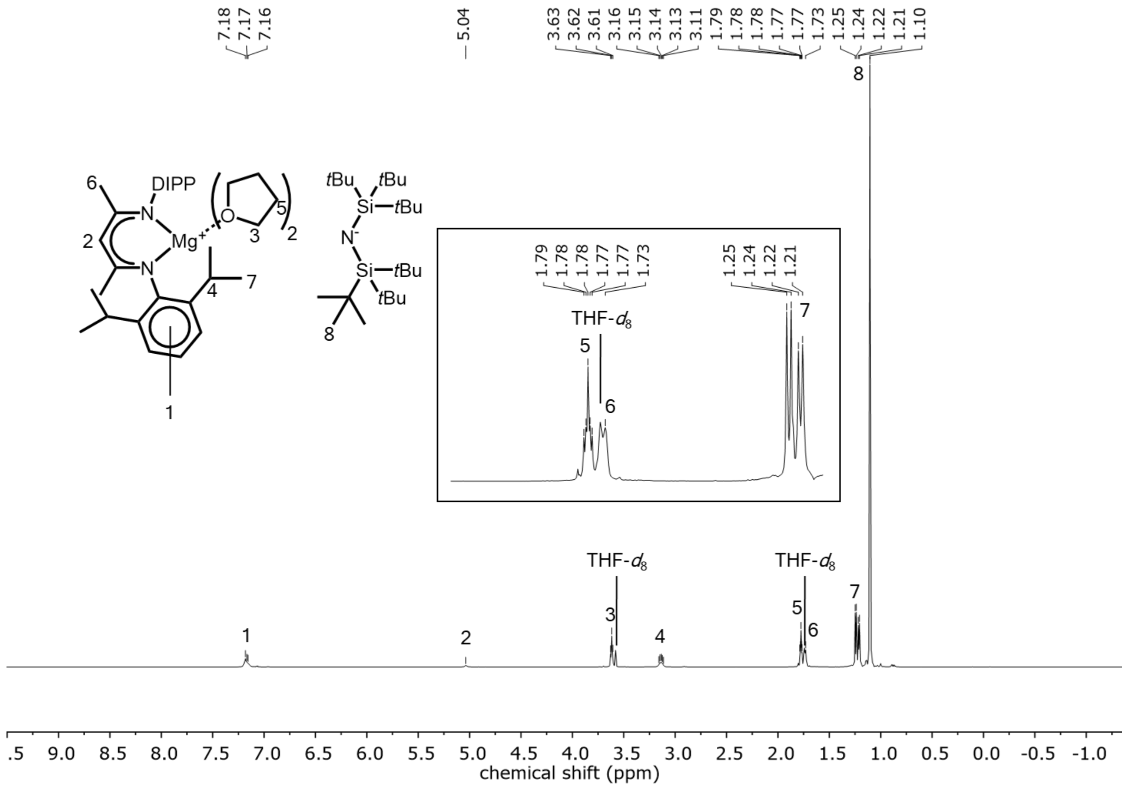

Figure S6: ^{1}H NMR spectrum of $[(BDI)Mg^{+}(THF)_2][tBuN^{-}]$ in THF-d_8 (600 MHz, 25 °C).

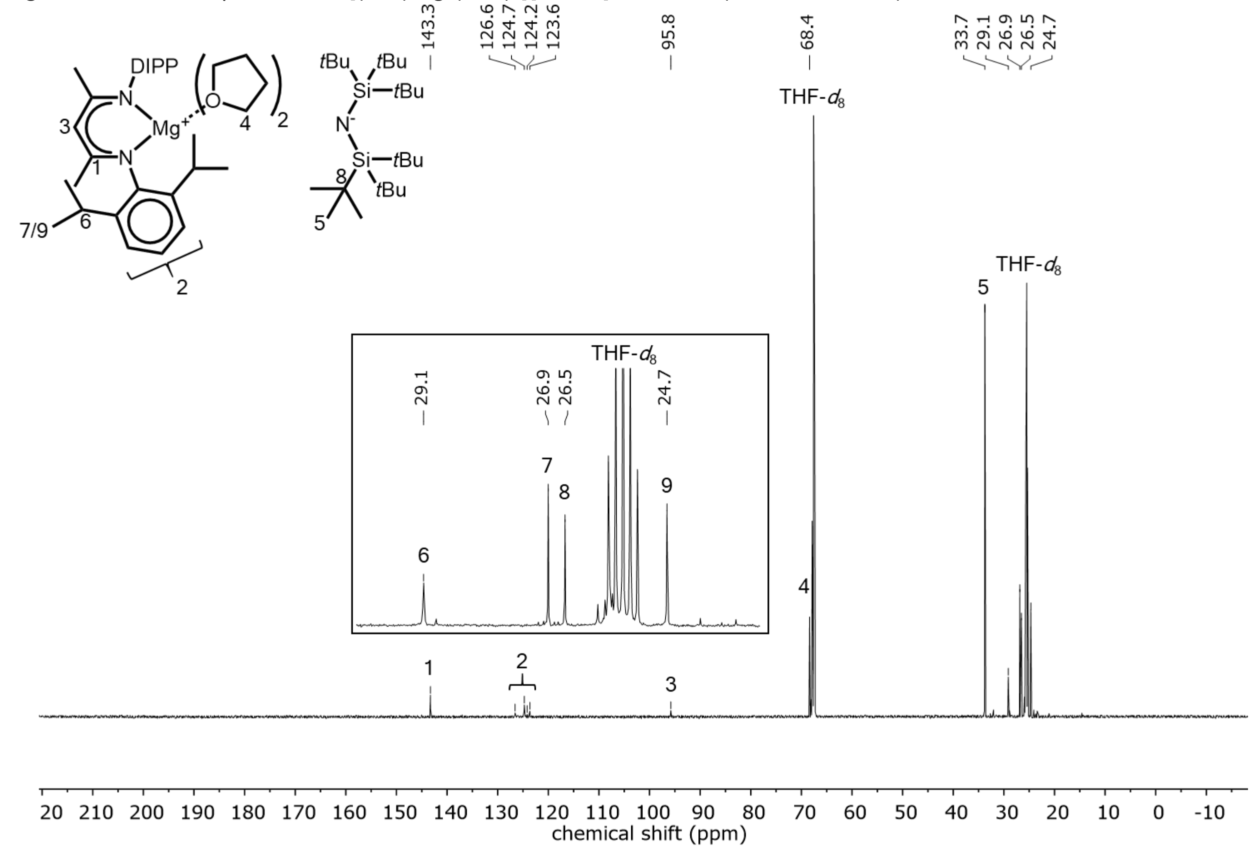

Figure S7: $^{13}C\{^{1}H\}$ NMR spectrum of $[(BDI)Mg^{+}(THF)_2][tBuN^{-}]$ in THF-d_8 (151 MHz, 25 °C).

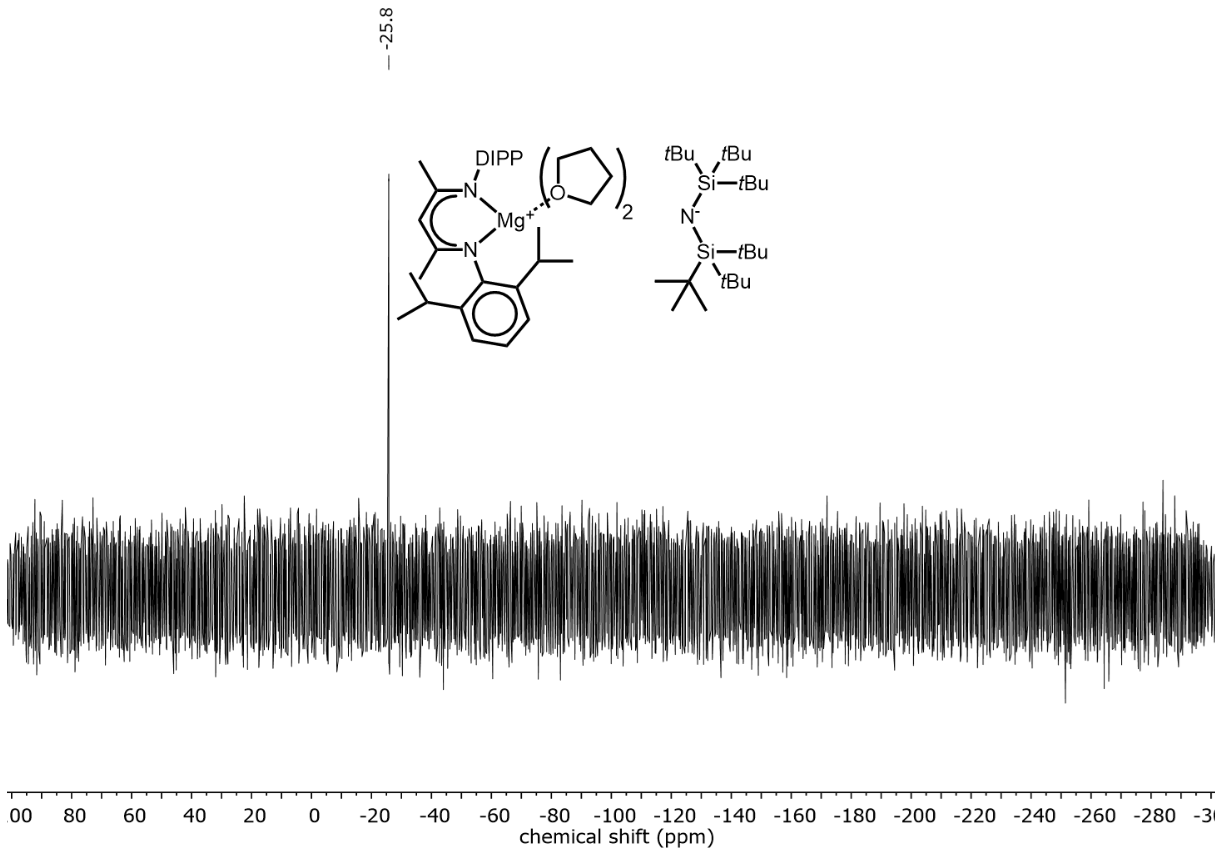

Figure S8: $^{29}Si\{^1H\}$ NMR spectrum of $[(BDI)Mg^+(THF)_2][tBuN^-]$ in THF-d_8 (119 MHz, 25 °C).

(^{tBu}N)Li (6)

(tBu$_3$Si)$_2$NCs (458 mg, 0.839 mmol) and LiI (228 mg, 1.70 mmol) were placed in a 5 mL Retsch stainless steel ball milling vessel equipped with 20 stainless steel balls (3 mm) and ground for 22 h at 30 Hz. The resulting colourless powder was extracted with hexane (2 x 4 mL) and the combined extracts were concentrated until incipient precipitation. The precipitate was redissolved by heating the suspension to 60 °C and after cooling to room temperature, large colourless crystals of (tBu$_3$Si)$_2$NLi were obtained (194 mg, 0.46 mmol, 55%).

^{1}H NMR (600 MHz, 25 °C, C_6D_6): δ_H = 1.10 (s, –Si(C(CH_3)$_3$)$_3$ ppm.

^{13}C{^{1}H} NMR (151 MHz, 25 °C, C_6D_6): δ_C = 32.3 (s, CMe_3), 25.5 (s, Si(CMe$_3$)$_3$ ppm.

^{7}Li NMR (233 MHz, 25 °C, C_6D_6): δ_{Li} = 1.0 ppm.

^{29}Si{^{1}H} NMR (119 MHz, 25 °C, C_6D_6): δ_{Si} = –15.4 ppm.

Elemental analysis: calculated (%) for C24H54LiNSi4 (419.81 g*mol^{-1}): C 68.66, H 12.97, N 3.34; found: C 70.67, H 13.33, N 3.38. Although these results are outside the range viewed as establishing analytical purity, they are provided to illustrate the best values obtained to date.

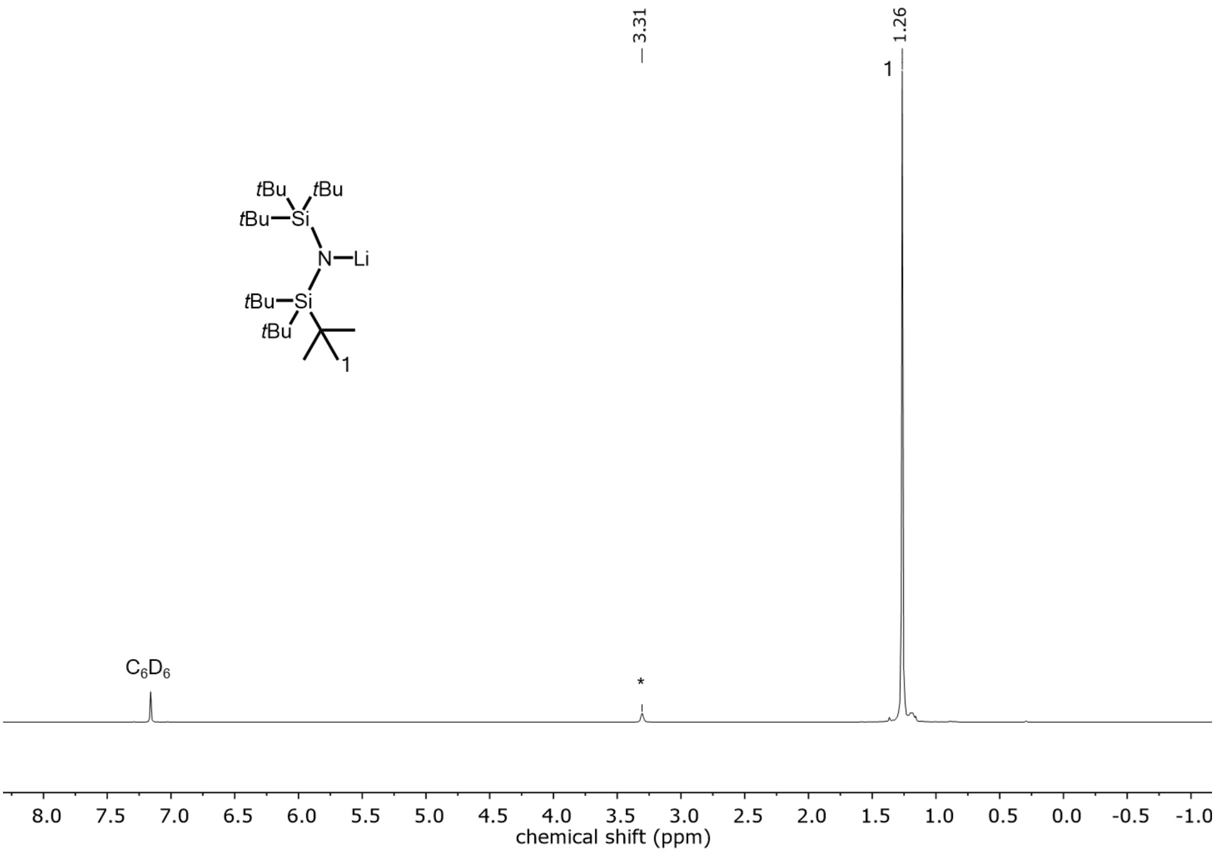

Figure S9: ^{1}H NMR spectrum of (tBu$_3$Si)$_2$NLi in C_6D_6 (600 MHz, 25 °C). * denotes unknown impurity.

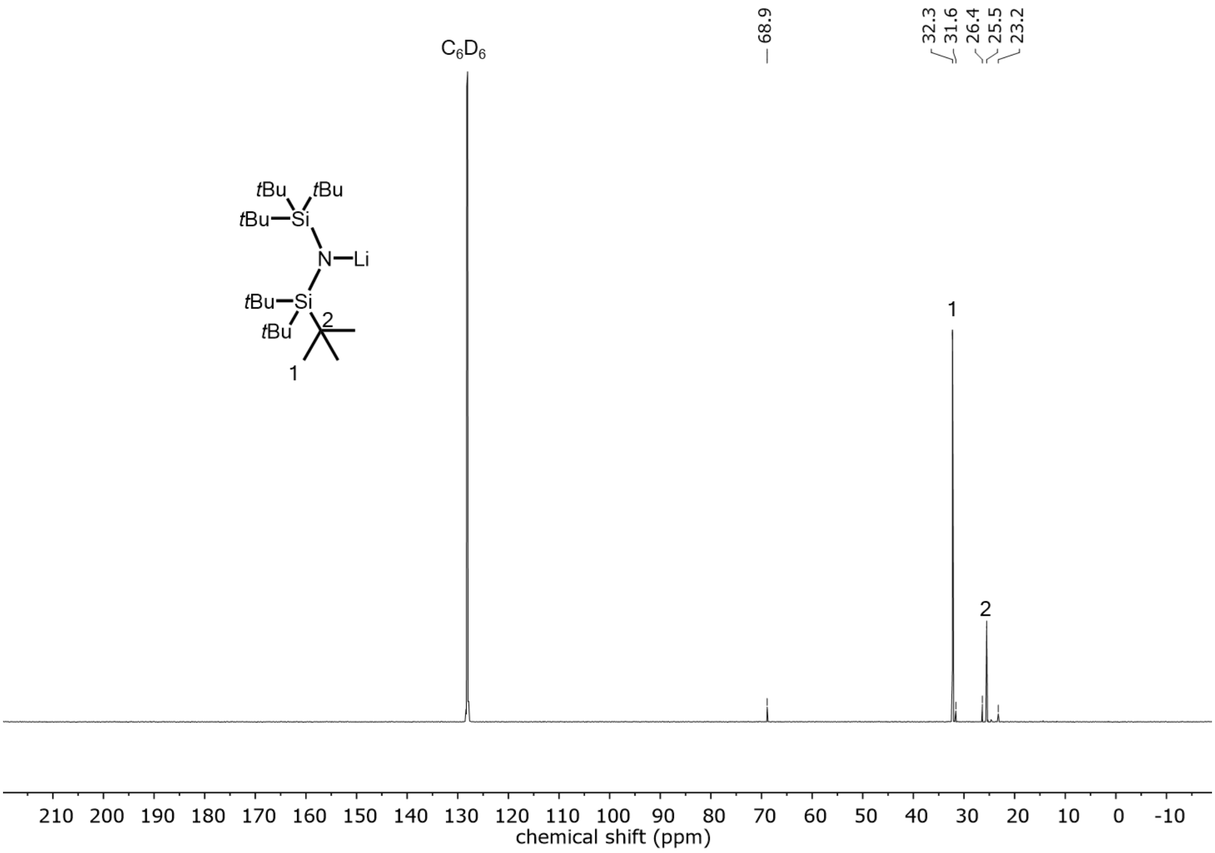

Figure S10: $^{13}C\{^{1}H\}$ NMR spectrum of $(tBu_3Si)_2NLi$ in C_6D_6 (151 MHz, 25 °C).

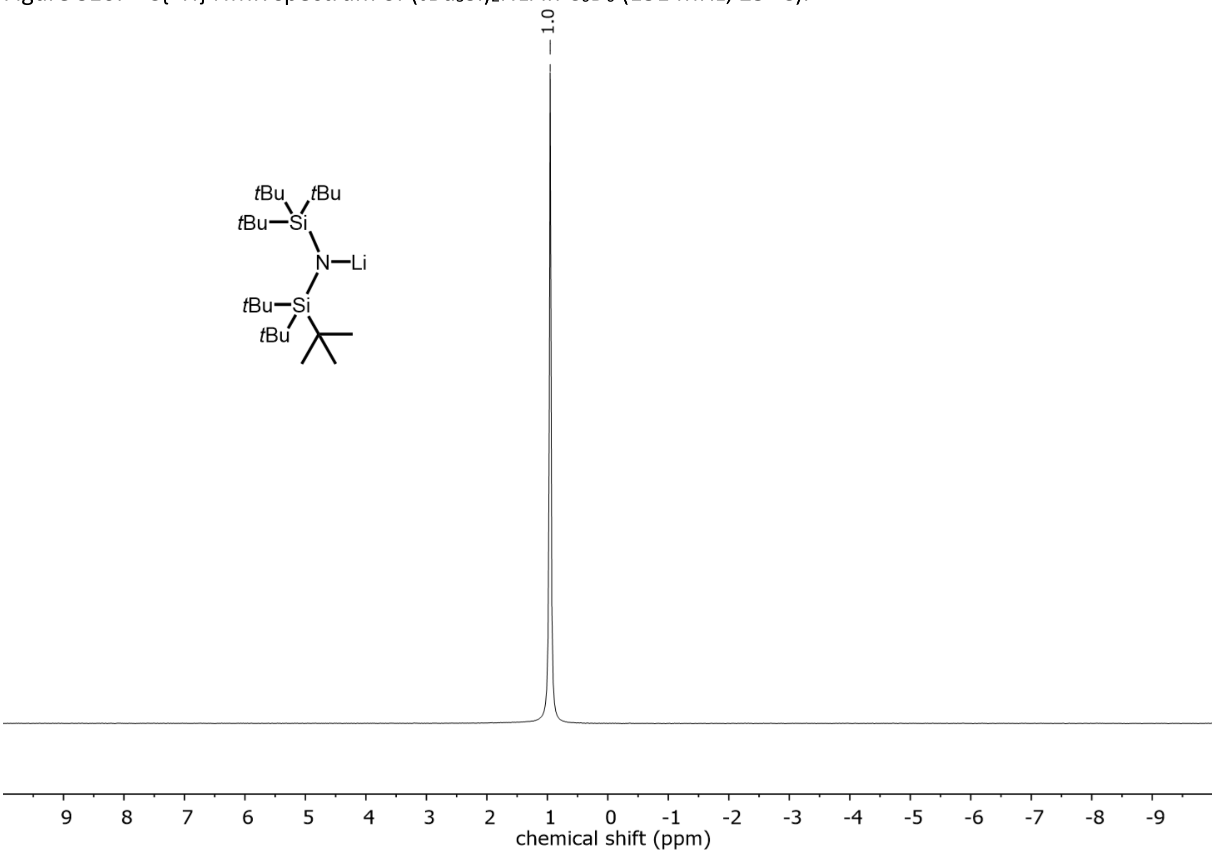

Figure S11: ^{7}Li NMR spectrum of $(tBu_3Si)_2NLi$ in C_6D_6 (233 MHz, 25 °C).

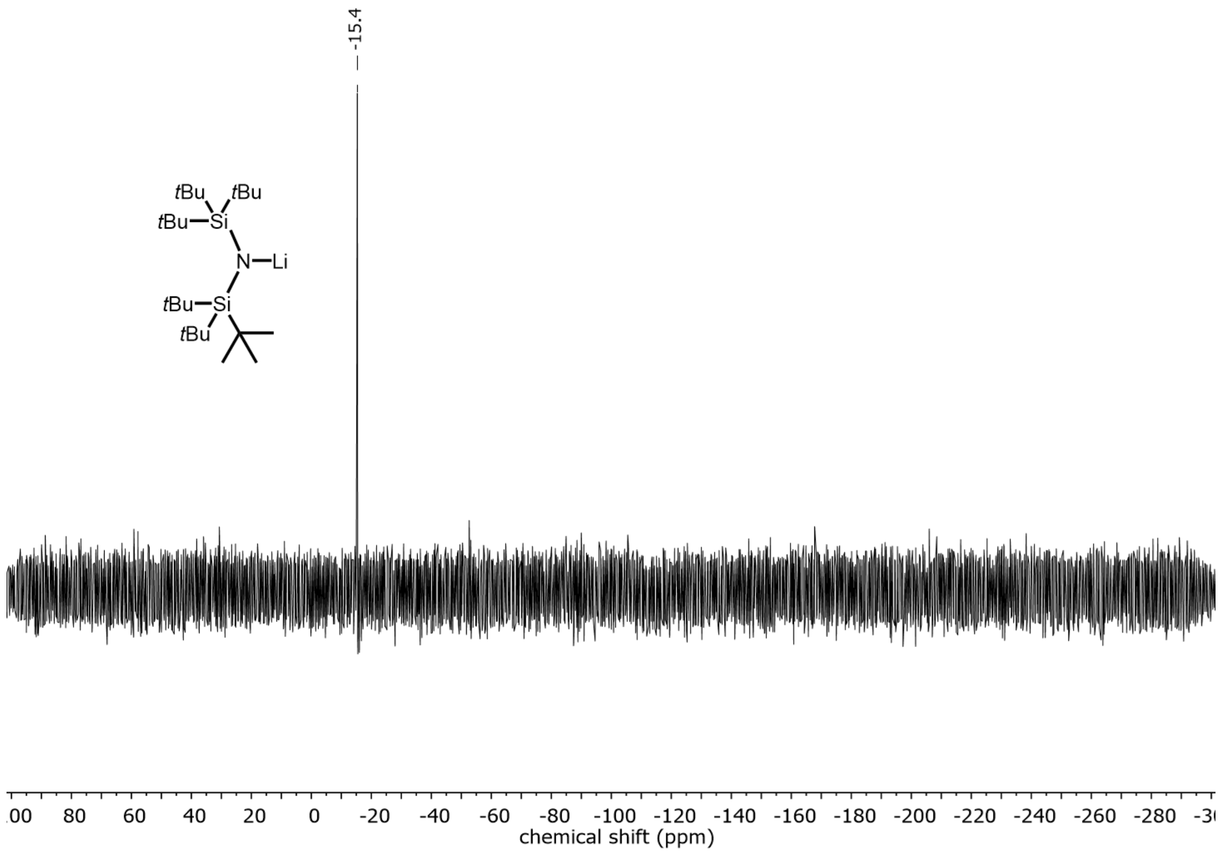

Figure S12: $^{29}Si\{^{1}H\}$ NMR spectrum of $(tBu_3Si)_2NLi$ in C_6D_6 (119 MHz, 25 °C).

[Li^+ (12-crown-4)$_2$] [$^{tBu}N^-$] (7)

(tBu$_3$Si)$_2$NLi (120 mg, 0.286 mmol) was dissolved in toluene (5 mL) and a solution of 12-crown-4 (109 mg, 100 µL, 0.618 mmol) in toluene (4 mL) was added while stirring. A colourless precipitate formed immediately. The reaction mixture was dried under dynamic vacuum and redissolved in a THP/methylcyclohexane mixture (approx. 1:1 by volume) and cooled to –30 °C. The resulting colourless crystals were separated from the mother liquor, washed with cold pentane (2 x 1 mL) and dried under vacuum to obtain [Li^+ ·(12-crown-4)$_2$] [$^{tBu}N^-$] (4) as a colourless solid (157 mg, 203 mmol, 71%). A second crop of crystals was obtained from the mother liquor.

^{1}H NMR (600 MHz, 25 °C, THF-d_8): δ_H = 3.69 (s, 32 H, –OCH_2–), 1.10 (s, 54 H,

–Si(C(CH_3)$_3$)$_3$ ppm.

^{13}C{^{1}H} NMR (151 MHz, 25 °C, THF-d_8): δ_C = 70.0 (s, 32 H, –OCH$_2$–), 33.7 (s,

–Si(CMe_3)$_3$), 26.9 (s, –Si(CMe$_3$)$_3$ ppm.

^{7}Li NMR (233 MHz, 25 °C, THF-d_8): δ_{Li} = –0.4 ppm.

^{29}Si{^{1}H} NMR (119 MHz, 25 °C, THF-d_8): δ_{Si} = –25.8 ppm.

Elemental analysis: calculated (%) for C40H85LiNO$_8$Si2 (772.24 g*mol^{-1}): C 62.30, H 11.11, N 1.82; found: C 62.66, H 11.08, N 1.79. Although these results are outside the range viewed as establishing analytical purity, they are provided to illustrate the best values obtained to date.

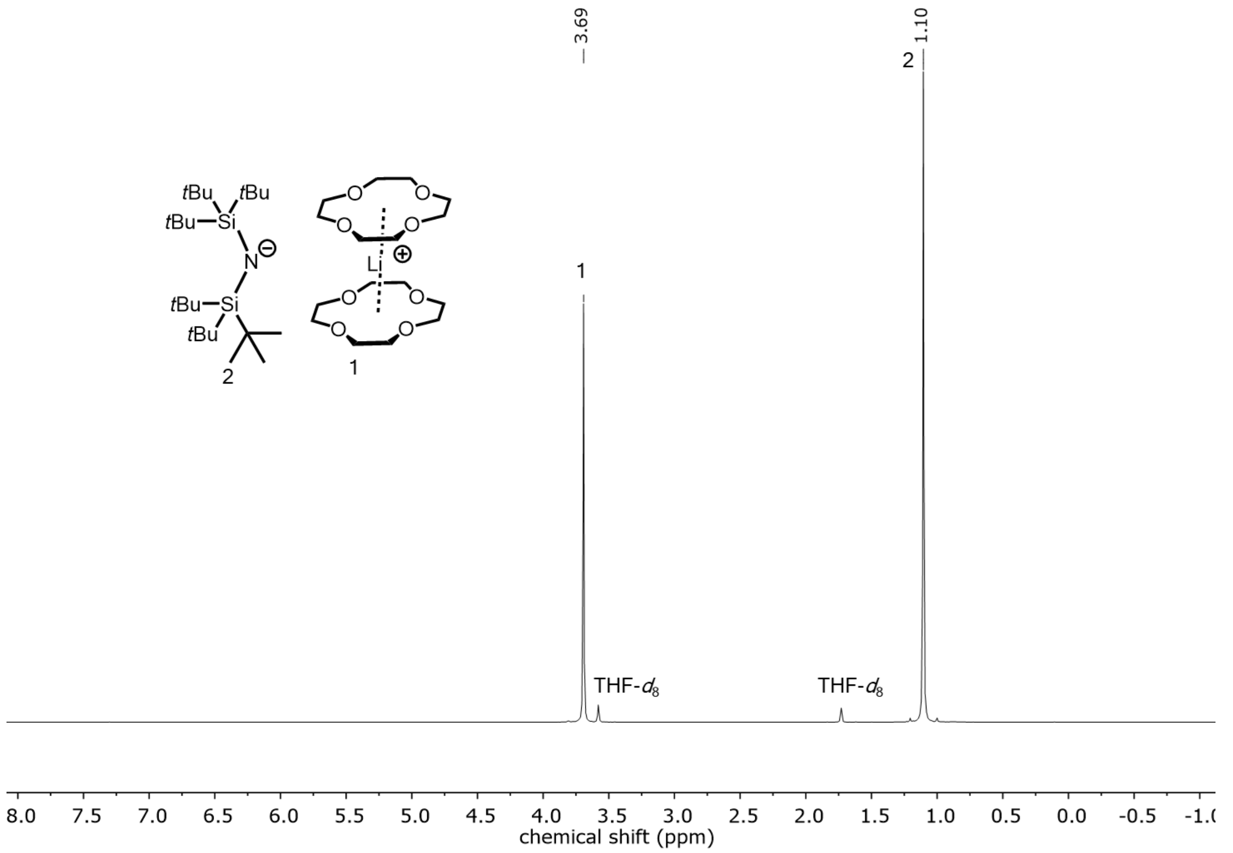

Figure S13: ^{1}H NMR spectrum of [Li^{+} (12-crown-4)$_2$] [$^{tBu}N^{-}$] in THF-d_8 (600 MHz, 25 °C).

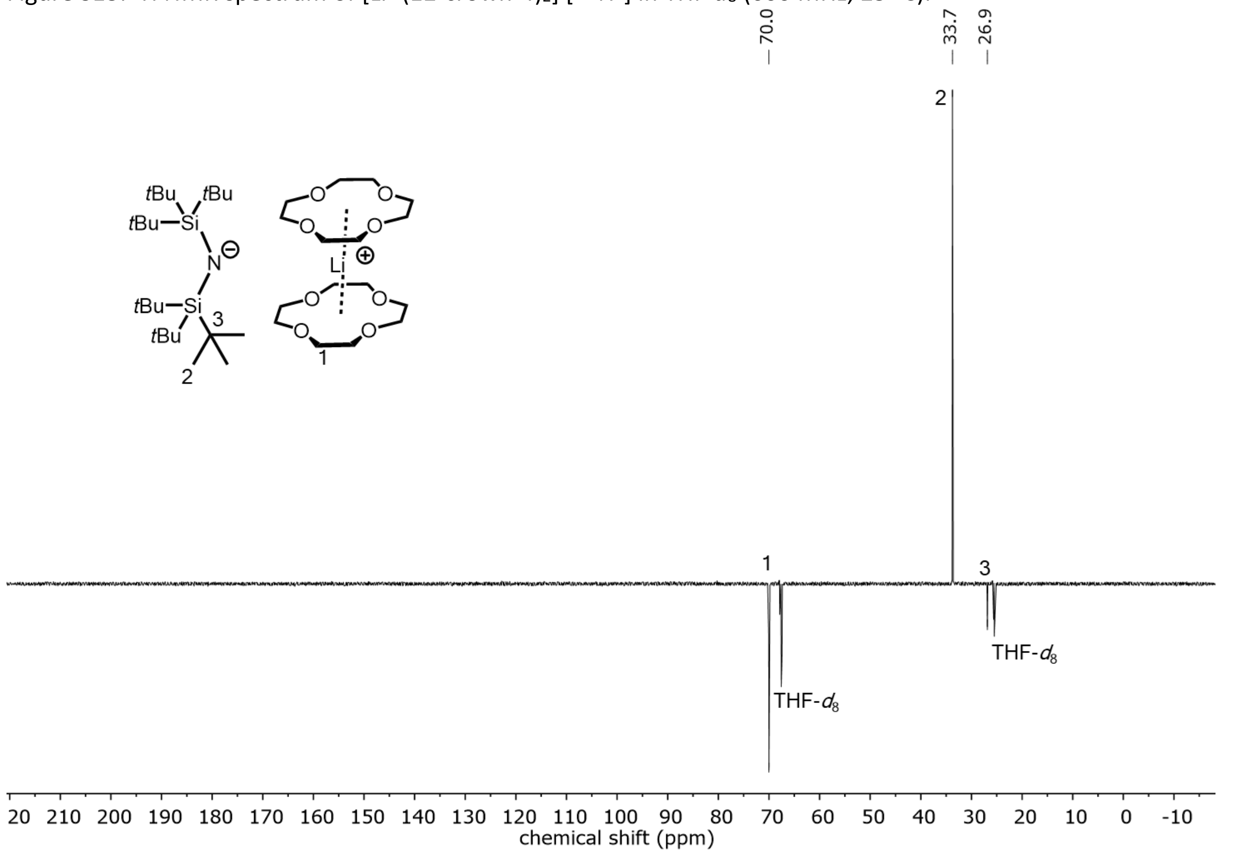

Figure S14: ^{13}C APT NMR spectrum of [Li^{+} (12-crown-4)$_2$] [$^{tBu}N^{-}$] in THF-d_8 (151 MHz, 25 °C).

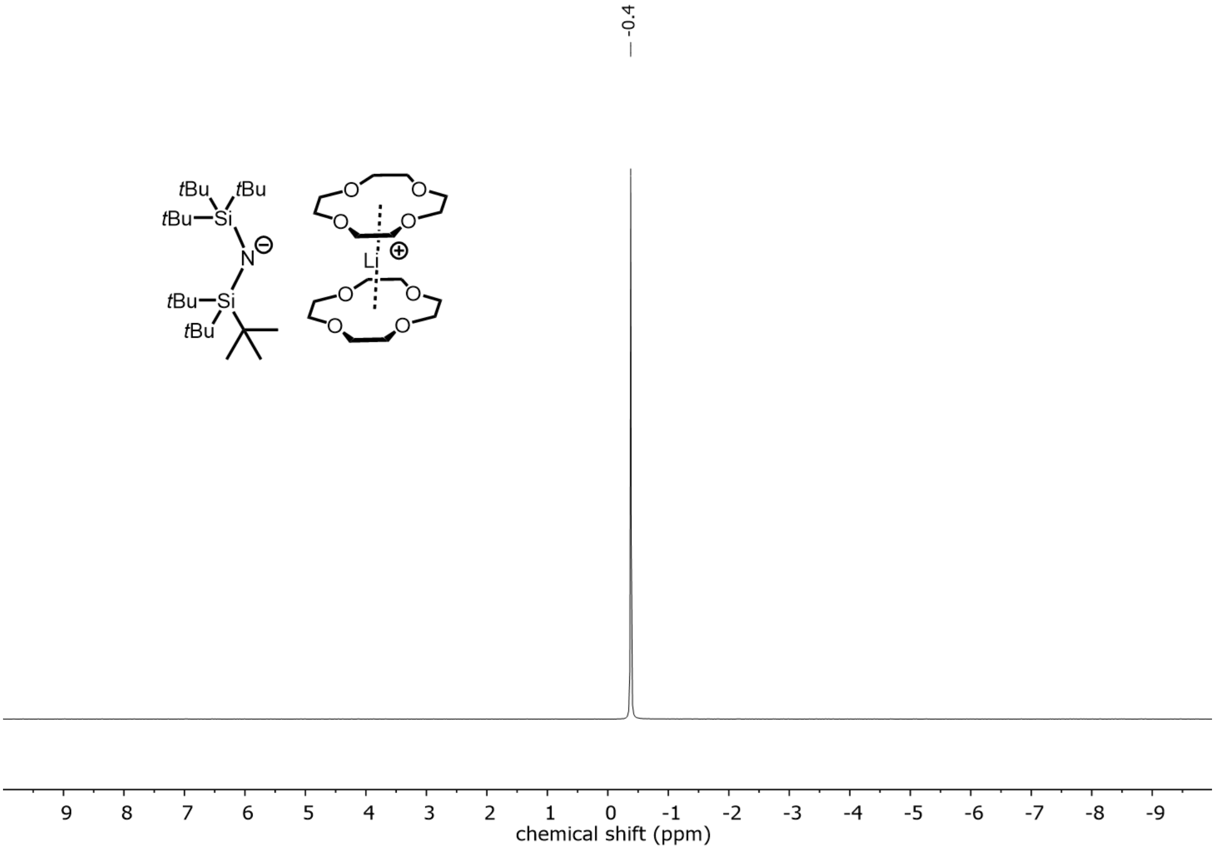

Figure S15: ^{7}Li NMR spectrum of [Li^{+} (12-crown-4)$_{2}$] [^{tBu}N^{-}] in THF-d_{8} (233 MHz, 25 °C).

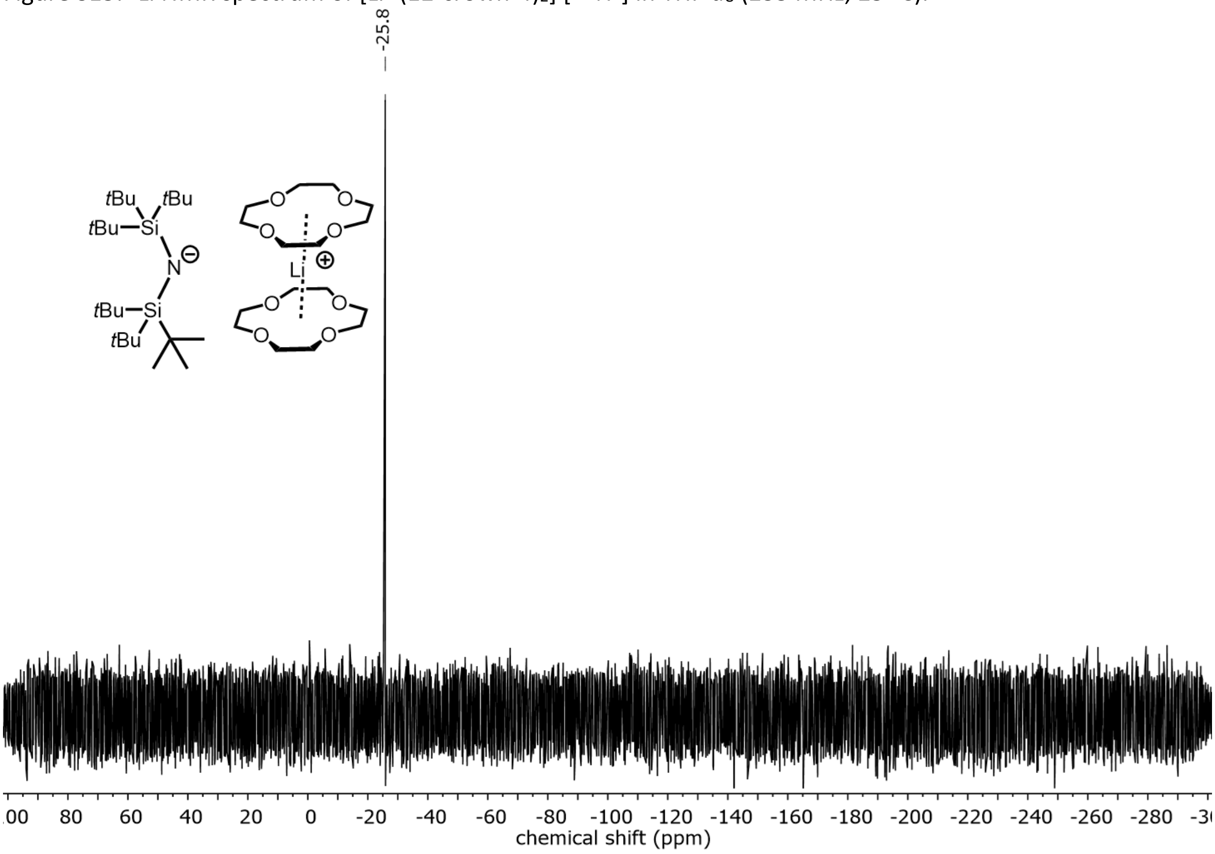

Figure S16: ^{29}Si{^{1}H} NMR spectrum of [Li^{+} (12-crown-4)$_{2}$] [^{tBu}N^{-}] in THF-d_{8} (119 MHz, 25 °C).

(^{iPr}N)Li (9)

(iPr$_3$Si)$_2$NH (2.04 g, 6.17 mmol) was dissolved in hexane (8 mL) and n-BuLi (2.5 M in hexanes, 2.5 mL, 6.25 mmol) was added dropwise at room temperature. The colourless solution was heated to 60 °C for 72 h. After cooling to room temperature, the solution was dried under reduced pressure, yielding (iPr$_3$Si)$_2$NLi as a colourless solid (2.42 g, 5.24 mmol, 85%). Diffraction quality crystals were obtained from a saturated solution of the amide in hexane or toluene at –30 °C.

^{1}H NMR (600 MHz, 25 °C, C_6D_6): δ_H = 1.20 (d, J = 7.6 Hz, 42H, –Si(CH(CH_3)$_2$)$_3$), 0.90 (hept, J = 7.4 Hz, 6H, –Si(CH(CH$_3$)$_2$)$_3$) ppm.

^{13}C{^{1}H} NMR (151 MHz, 25 °C, C_6D_6): δ_C = 20.3 (s, CHMe_2), 16.2 (s, Si(CHMe$_2$)$_3$ ppm.

^{7}Li NMR (233 MHz, 25 °C, C_6D_6): δ_{Si} = –1.4 ppm.

^{29}Si{^{1}H} NMR (119 MHz, 25 °C, C_6D_6): δ_{Si} = –9.4 ppm.

Elemental analysis: calculated (%) for $C_{18}H_{42}LiNSi2$ (335.65 g*mol^{-1}): C 64.41, H 12.61, N 4.17; found: C 62.49, H 12.21, N 3.95. Although these results are outside the range viewed as establishing analytical purity, they are provided to illustrate the best values obtained to date.

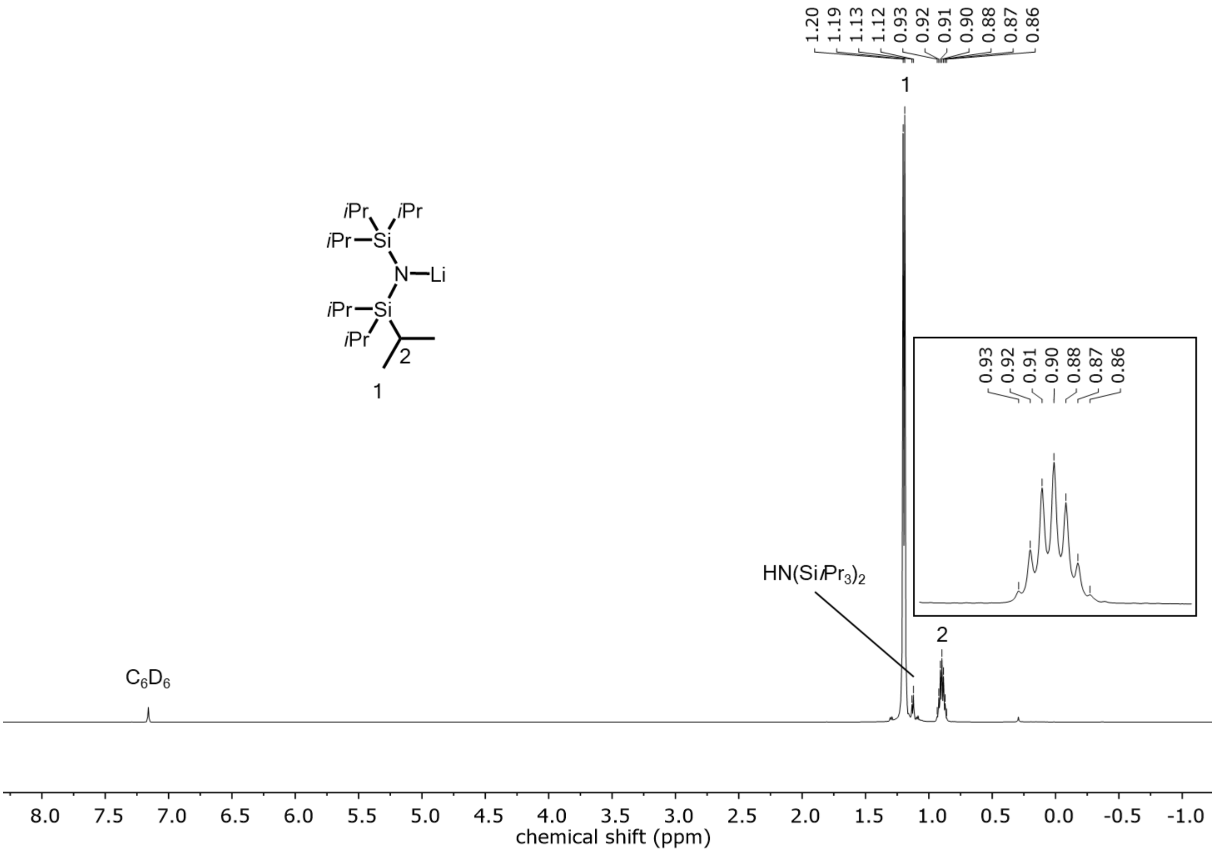

Figure S17: ^{1}H NMR spectrum of (iPr$_3$Si)$_2$NLi in C_6D_6 (600 MHz, 25 °C).

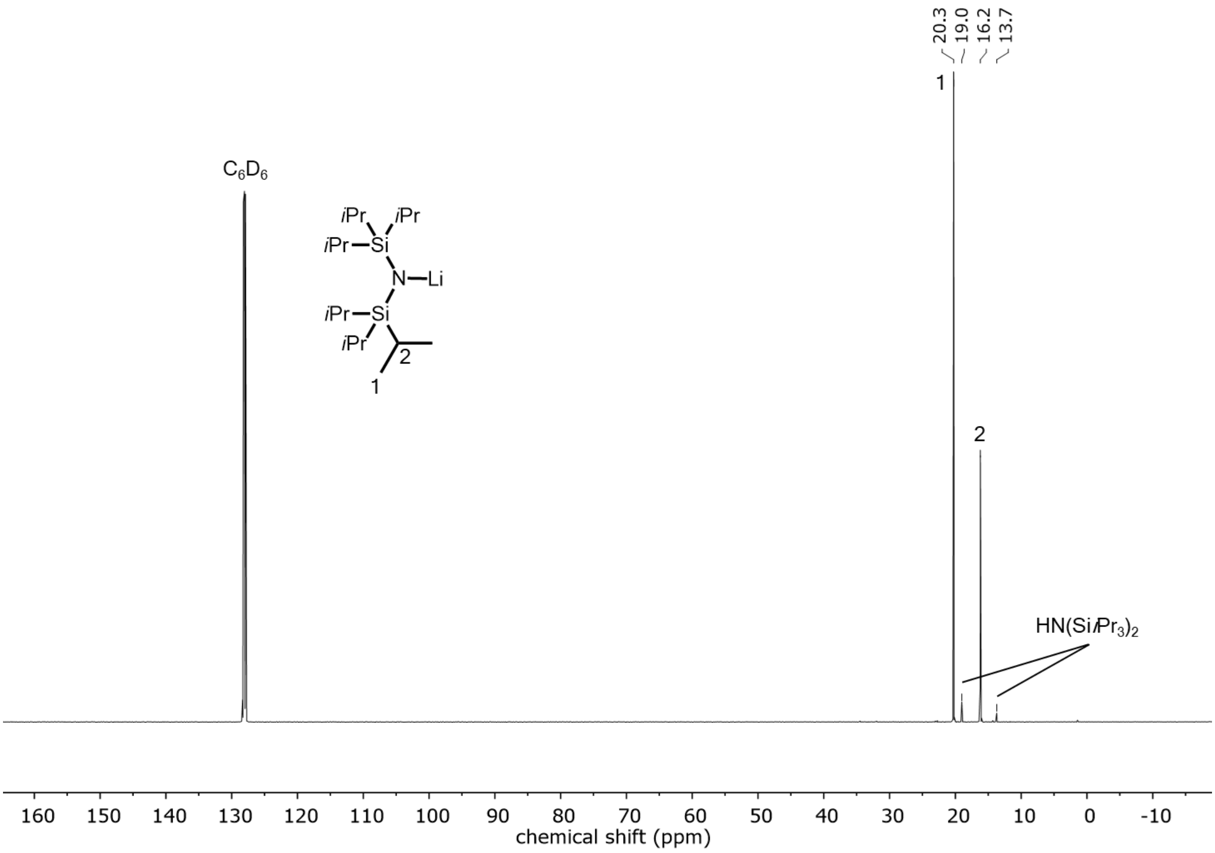

Figure S18: $^{13}C\{^{1}H\}$ NMR spectrum of ($i$$Pr_3Si)_2NLi$ in C_6D_6 (151 MHz, 25 °C).

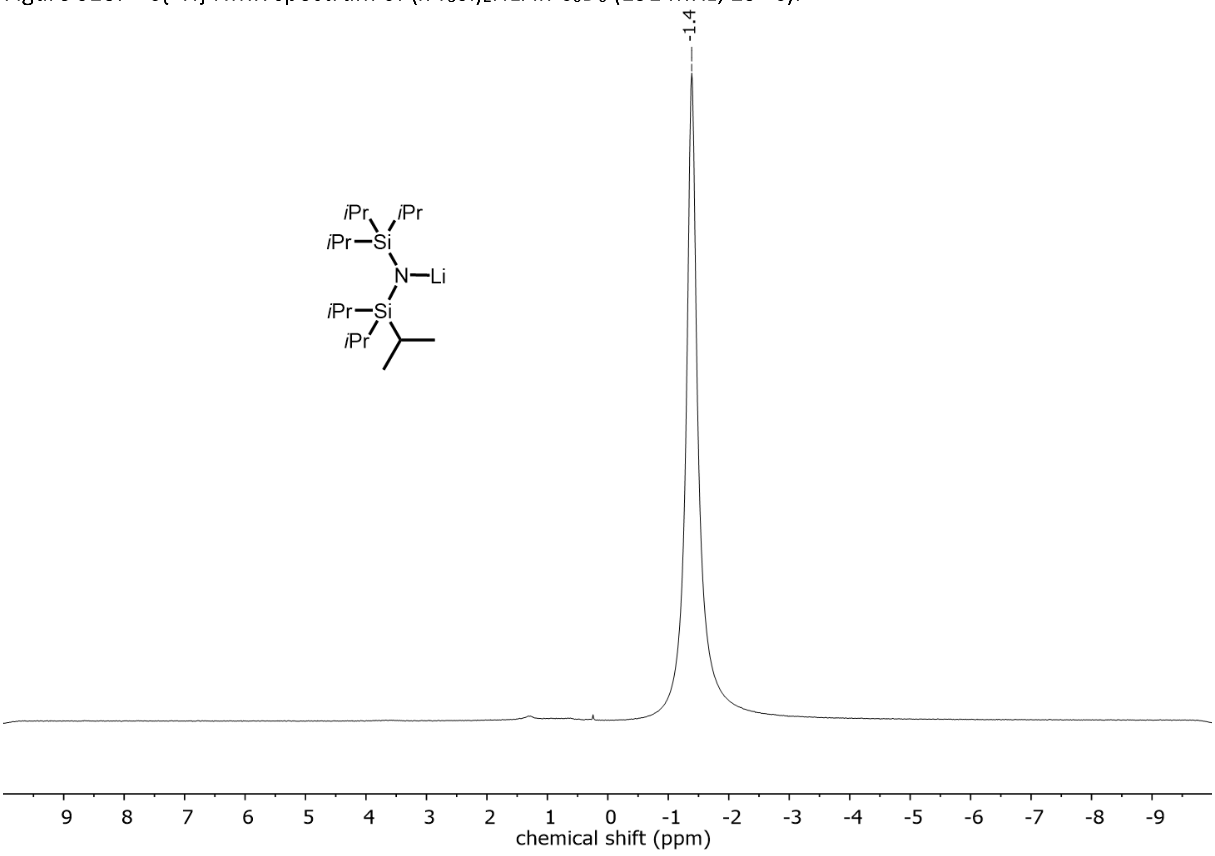

Figure S19: ^{7}Li NMR spectrum of ($i$$Pr_3Si)_2NLi$ in C_6D_6 (233 MHz, 25 °C).

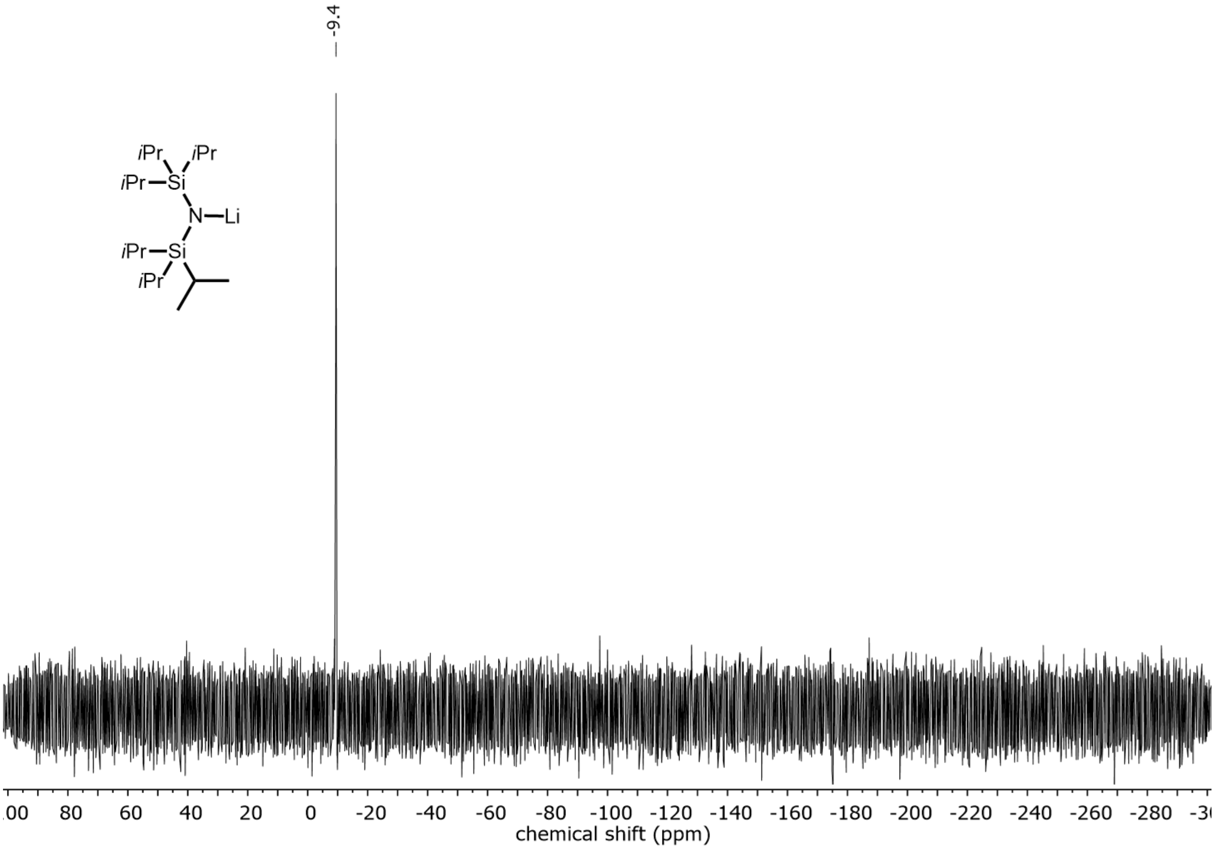

Figure S20: $^{29}Si\{^{1}H\}$ NMR spectrum of (iPr$_3$Si)$_2$NLi in C_6D_6 (119 MHz, 25 °C).

(^{iPr}N)Cs (10)

A Schlenk flask equipped with a glass coated stirring bar was charged with cesium (1.05 g, 7.90 mmol) and THP (10 mL). (iPr$_3$Si)$_2$NH (0.99 g, 3.00 mmol) was added and the mixture heated to 40 °C for 2 d. The brown supernatant solution was decanted and the residue extracted with THP (12 mL). The combined extracts were dried and redissolved in toluene (10 mL) to displace coordinated THP. After drying under reduced pressure, the resulting colourless solid was washed with pentane (3 x 5 mL) to yield (iPr$_3$Si)$_2$NCs (947 mg, 2.05 mmol, 68%). Crystals suitable for X-ray diffraction analysis were obtained by diffusing hexane into a saturated solution of the amide in toluene at –30 °C.

^{1}H NMR (600 MHz, 25 °C, C_6D_6): δ_H = 1.37 (d, $J_{H–H}$ = 7.5 Hz, 36H) –Si(CH(CH_3)$_2$)$_3$), 0.96 (sep, $J_{H–H}$ = 7.5 Hz, 6H, –Si(CH(CH$_3$)$_2$)$_3$) ppm.

^{13}C{^{1}H} NMR (151 MHz, 25 °C, C_6D_6): δ_C = 20.8 (s, CHMe_2), 17.3 (s, Si(CHMe$_2$)$_3$ ppm.

^{29}Si{^{1}H} NMR (119 MHz, 25 °C, C_6D_6): δ_{Si} = –17.1 ppm.

Elemental analysis: calculated (%) for C18H$_{42}$CsNSi2 (461.62 g*mol^{-1}): C 46.83, H 9.17, N 3.03; found: C 47.46, H 9.23, N 3.02.

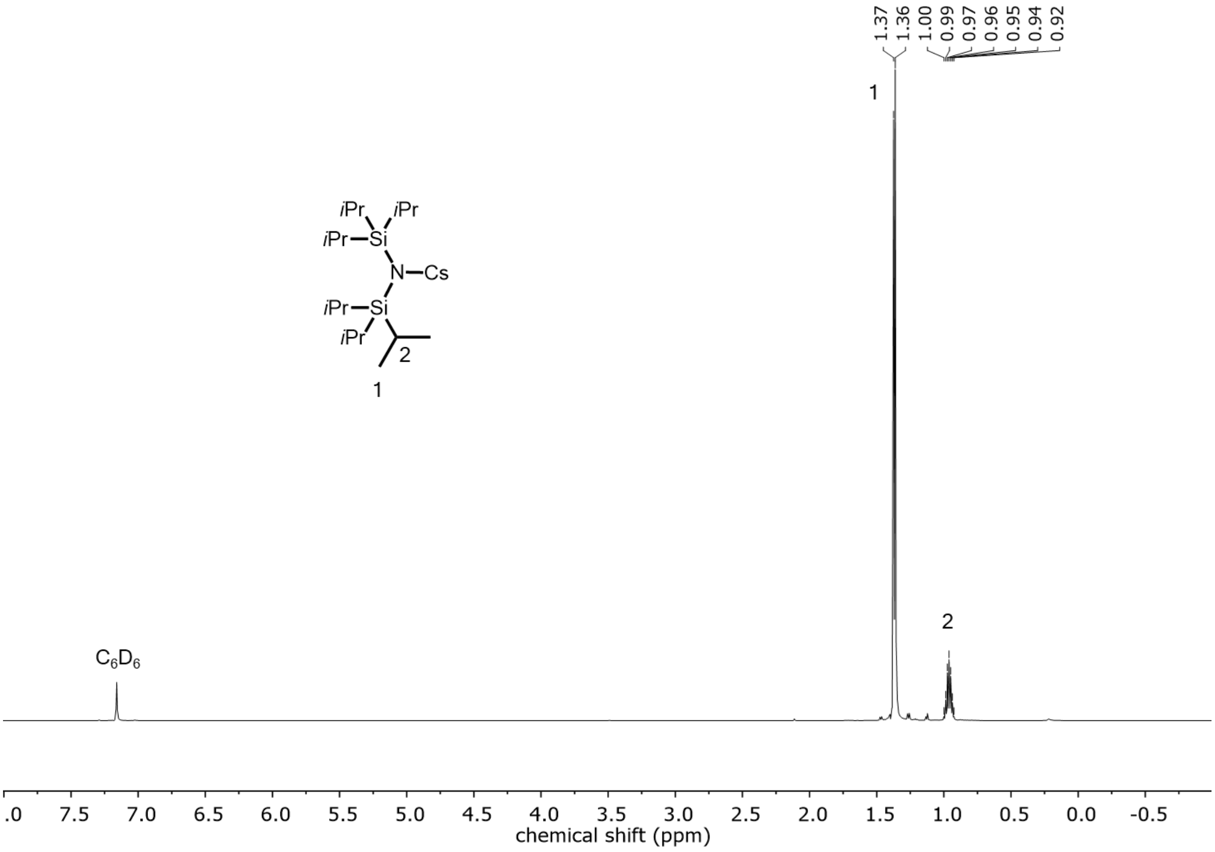

Figure S21: ^{1}H NMR spectrum of (iPr$_3$Si)$_2$NCs in C_6D_6 (600 MHz, 25 °C).

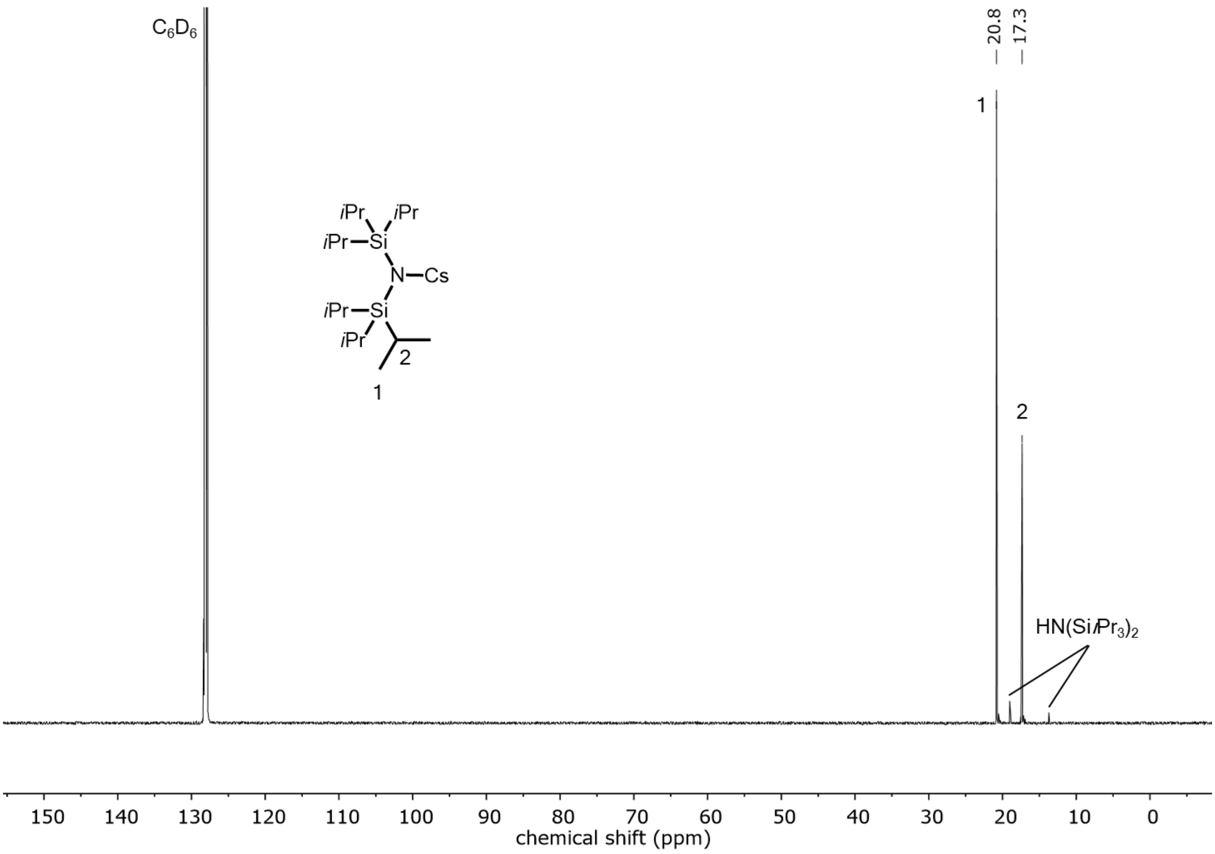

Figure S22: $^{13}C\{^1H\}$ NMR spectrum of ($i$$Pr_3Si)_2NCs$ in C_6D_6 (151 MHz, 25 °C).

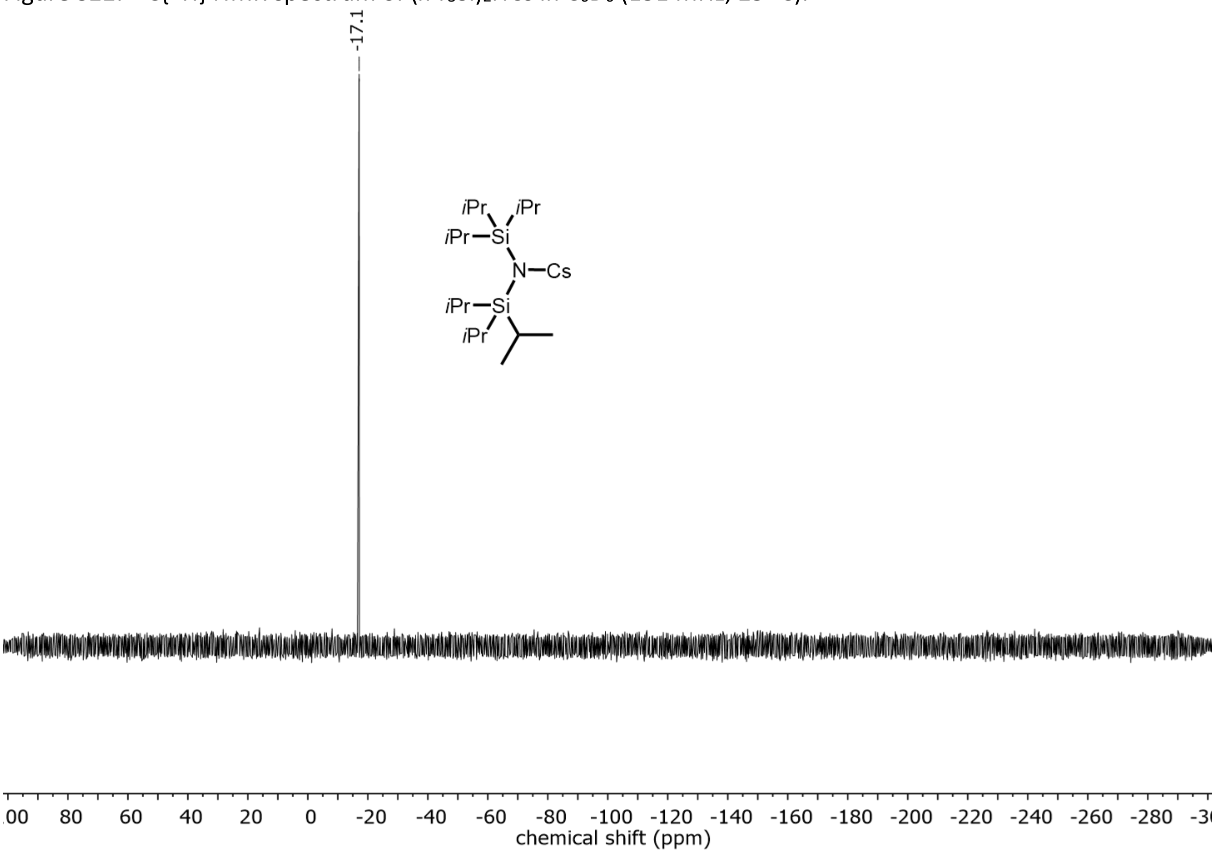

Figure S23 $^{29}Si\{^1H\}$ NMR spectrum of ($i$$Pr_3Si)_2NCs$ in C_6D_6 (119 MHz, 25 °C).

Reaction of ^{tBu}NH with K in liquid NH_3

Ammonia (approx. 40 mL) were condensed into a Schlenk flask containing freshly cut chunks of potassium (51.7 mg, 1.32 mmol) at –78 °C. To the resulting dark blue solution was added a solution of $(tBu_3Si)_2NH$ (551.7 mg, 1.33 mmol) in pentane (12 mL) dropwise. This led to rapid loss of the blue color. After 2 h, a colourless suspension was obtained. Subsequently, the cooling bath was removed and the ammonia evaporated, resulting in a grey solid. Extraction of the solid with pentane (3 x 10 mL) and drying of the combined extracts led to quantitative recovery of the starting amine. The residue after pentane extraction was insoluble in THF. Hydrolysis of the insoluble grey residue resulted in formation of gaseous ammonia indicated by its characteristic smell and its strongly basic properties (wet indicator paper over the solution turned immediately blue). Thus, the residue is most likely KNH_2. Using different solvent mixtures (such as NH_3/Et_2O or NH_3/THF) did not lead to a different outcome.

Reaction of ^{tBu}NH with K and 18-crown-6

A J. Young NMR tube was charged with $(tBu_3Si)_2NH$ (38.1 mg, 92.1 µmol), K (4.1 mg, 105 µmol), 18-crown-6 (22.6 mg, 85.5 µmol) and THF-d_8 (500 µL). The dark blue solution turned colourless within 90 min. ^{1}H NMR spectroscopic monitoring showed no conversion of the amine and decomposition of the crown ether.

DFT Calculations

All calculations were carried out using Gaussian 16A.[7] All methods were used as implemented. All structures were fully optimized on a B3PW91/def2TZVP level of theory.[8–11] All structures were characterized as true minima (Nimag=0) by frequency calculations on the same level of theory. Energies were determined at a B3PW91/def2TZVP level of theory. In all cases Grimme's third dispersion correction with Becke-Johnson damping (GD3BJ) was applied, unless indicated otherwise.[12] All structures were evaluated using Molecule 2.3.[13] Topological analyses were carried out using AIMAll V17 with the wavefunctions obtained from the B3LYP/def2TZVP calculations.[14,15]

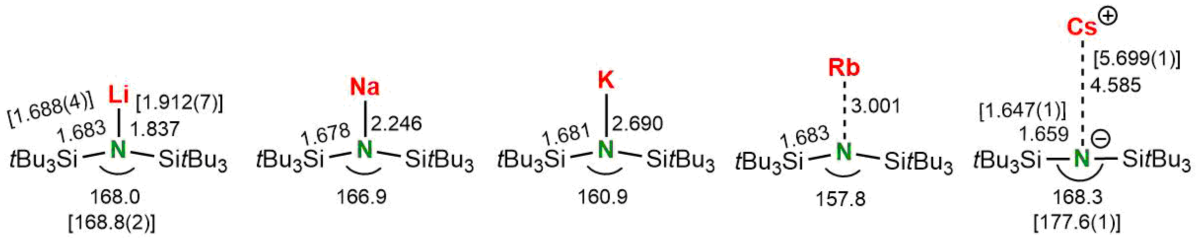

Figure S24: Selected bond distances (Å) and angles for calculated structures of bulky alkali metal amides (B3PW91/def2TZVP incl dispersion). Values between square brackets relate to crystal structures.

Table S1: Selected structural parameters of calculated $(R_3Si)_2N–M$ structures.

	M	R	N–M (Å)	Si–N (Å)	Si–N–Si (°)
1	H	Me	1.0098	1.7335, 1.7336	133.03
2	H	*i*Pr	1.0155	1.7350, 1.7359	143.13
3	H	*t*Bu	1.0175	1.7586, 1.7594	161.19
4	Li	Me	1.7842	1.6794, 1.6892	133.82
5	Li	Me no dispersion	1.7962	1.6089, 1.6914	139.38
6	Li	*i*Pr	1.8209	1.6710, 1.6717	152.71
7	Li	*i*Pr no dispersion	1.8350	1.6737, 1.6806	159.86
8	Li	*t*Bu	1.8373	1.6795, 1.6862	167.99
9	Li	*t*Bu no dispersion	1.8519	1.6930. 1.7008	167.26
10	Na	*t*Bu	1.2456	1.6778, 1.6779	166.91
11	K	*t*Bu	2.6901	1.6808, 1.6809	160.85
12	Rb	*t*Bu	3.0007	1.6833, 1.6838	157.84
13	Cs	Me	2.7232	1.6762, 1.6762	130.84
14	Cs	Me no dispersion	2.7574	1.6795, 1.6795	134.23
15	Cs	*i*Pr	2.8603	1.6718, 1.6722	141.36
16	Cs	*i*Pr no dispersion	2.9163	1.6801, 1.6805	144.29
17	Cs	*t*Bu	4.5852	1.6585, 1.6586	168.32
18	Cs	*t*Bu no dispersion	4.7508	1.6681, 1.6683	170.14
19	Free amide	Me	-	1.6432, 1.6432	141.84
20	Free amide	Me no dispersion	-	1.6335, 1.6335	179.75
21	Free amide	*i*Pr	-	1.6369, 1.6386	158.13
19	Free amide	*i*Pr no dispersion	-	1.6433, 1.6456	166.22
20	Free amide	*t*Bu	-	1.6481, 1.6487	179.99
21	Free amide	*t*Bu no dispersion	-	1.6594, 1.6598	180

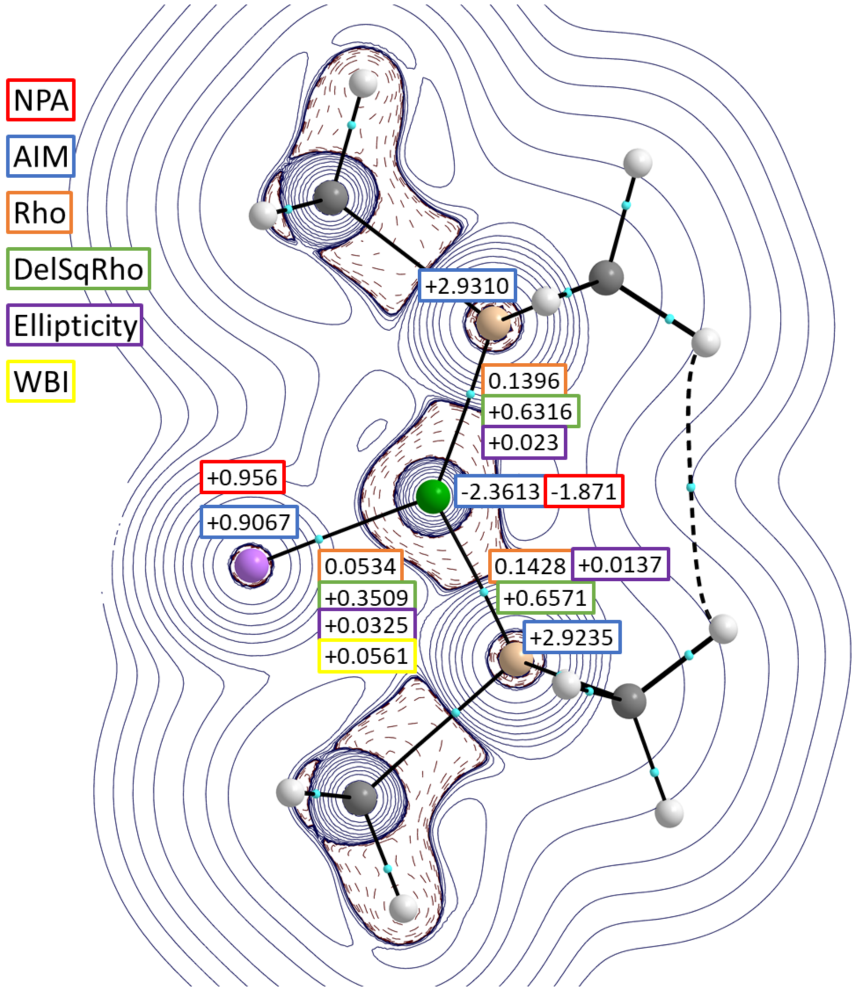

Figure S25: Atoms-In-Molecules: Contour plots of the Laplacian, $\nabla^2\rho(r)$, of (^{Me}N)Li (calculated with dispersion) through the plane formed by the N and Si atoms. Light-blue dots: bond critical point (BCP); red boxes: NPA charges; blue boxes: AIM charges; orange boxes: electron densities in the BCPs; green boxes: Laplacian $\nabla^2\rho$ in BCPs; purple boxes: bond ellipticity. Red dashed lines indicate areas of charge concentration, while blue solid lines show areas of charge depletion. Most hydrogen atoms were omitted for clarity.

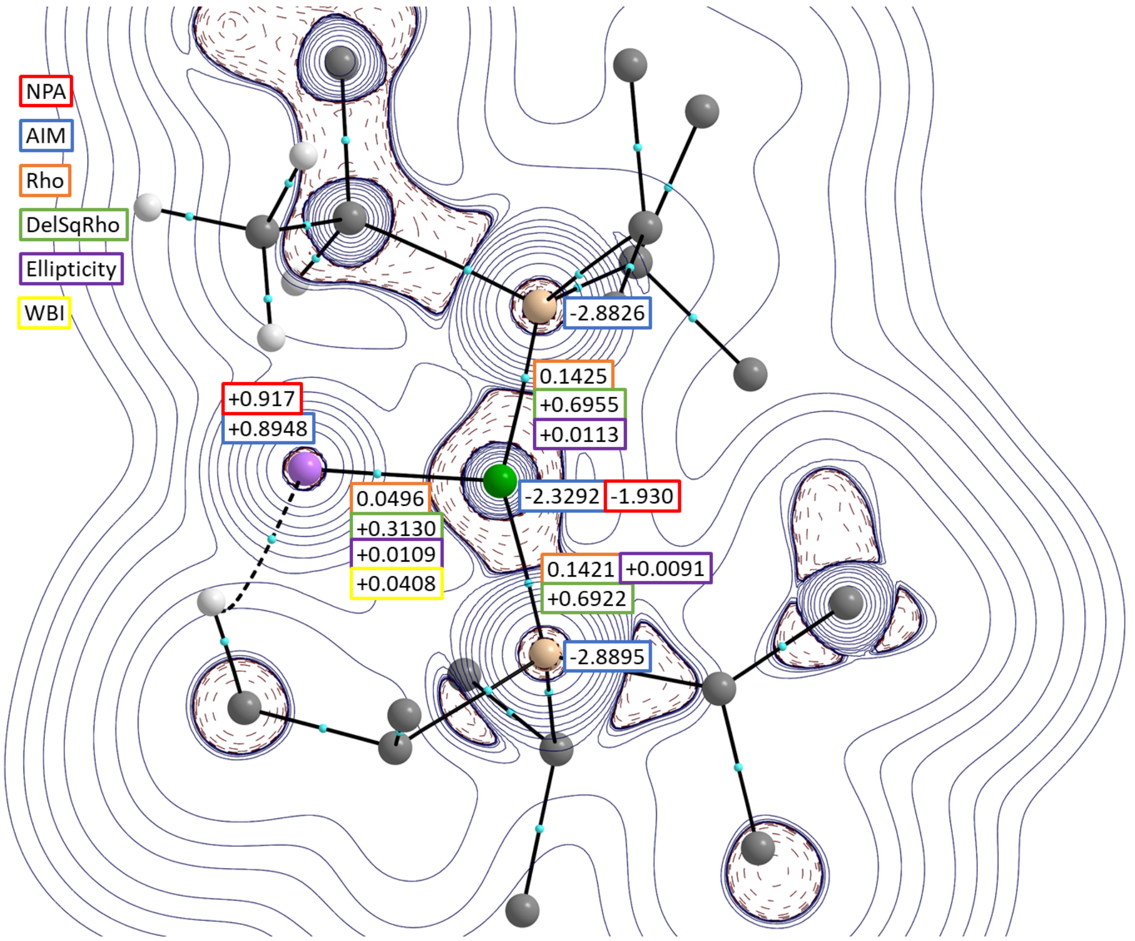

Figure S26: Atoms-In-Molecules: Contour plots of the Laplacian, $\nabla^2\rho(r)$, of (^{iPr}N)Li (calculated with dispersion) through the plane formed by the N and Si atoms. Light-blue dots: bond critical point (BCP); red boxes: NPA charges; blue boxes: AIM charges; orange boxes: electron densities in the BCPs; green boxes: Laplacian $\nabla^2\rho$ in BCPs; purple boxes: bond ellipticity. Red dashed lines indicate areas of charge concentration, while blue solid lines show areas of charge depletion. Most hydrogen atoms were omitted for clarity.

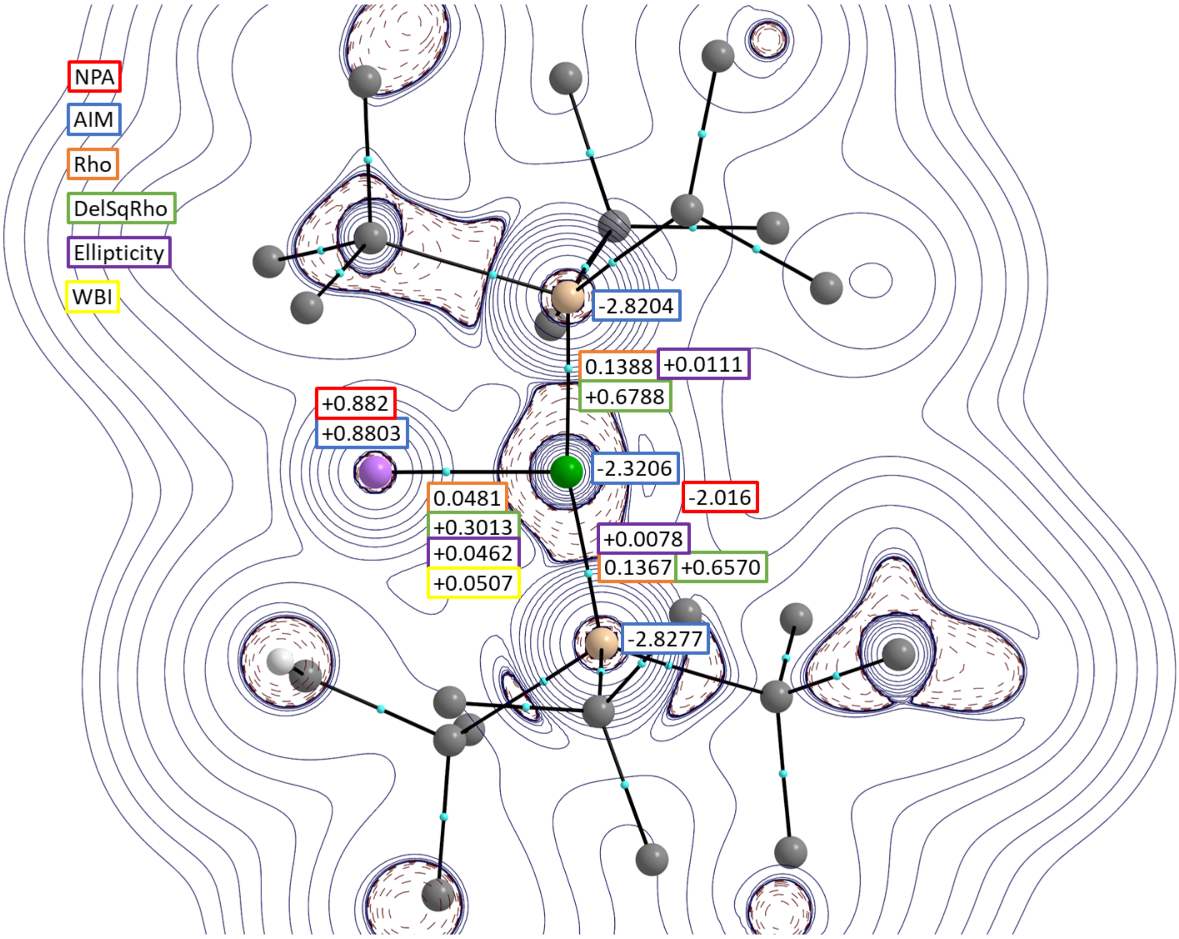

Figure S27: Atoms-In-Molecules: Contour plots of the Laplacian, $\nabla^2\rho(r)$, of (^{tBu}N)Li (calculated with dispersion) through the plane formed by the N and Si atoms. Light-blue dots: bond critical point (BCP); red boxes: NPA charges; blue boxes: AIM charges; orange boxes: electron densities in the BCPs; green boxes: Laplacian $\nabla^2\rho$ in BCPs; purple boxes: bond ellipticity. Red dashed lines indicate areas of charge concentration, while blue solid lines show areas of charge depletion. Most hydrogen atoms were omitted for clarity.

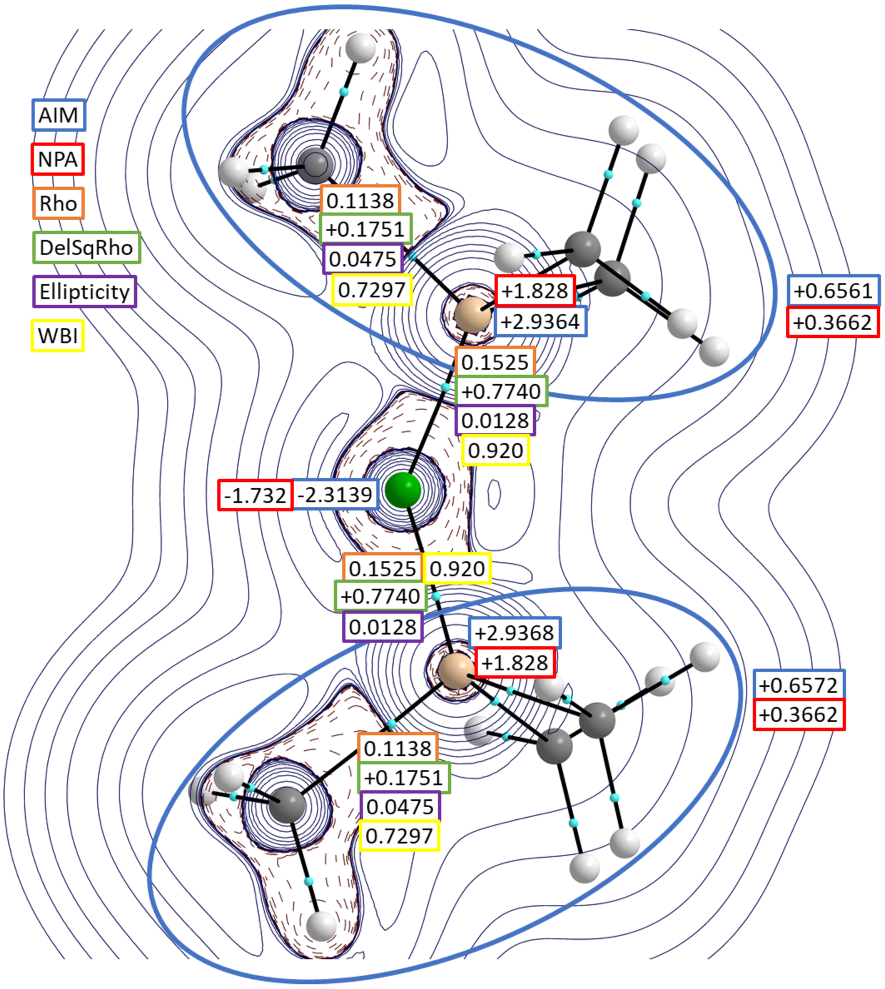

Figure S28: Atoms-In-Molecules: Contour plots of the Laplacian, $\nabla^2\rho(r)$, of free $(^{Me}N)^-$ (calculated with dispersion) through the plane formed by the N and Si atoms. Light-blue dots: bond critical point (BCP); red boxes: NPA charges; blue boxes: AIM charges; orange boxes: electron densities in the BCPs; green boxes: Laplacian $\nabla^2\rho$ in BCPs; purple boxes: bond ellipticity. Red dashed lines indicate areas of charge concentration, while blue solid lines show areas of charge depletion.

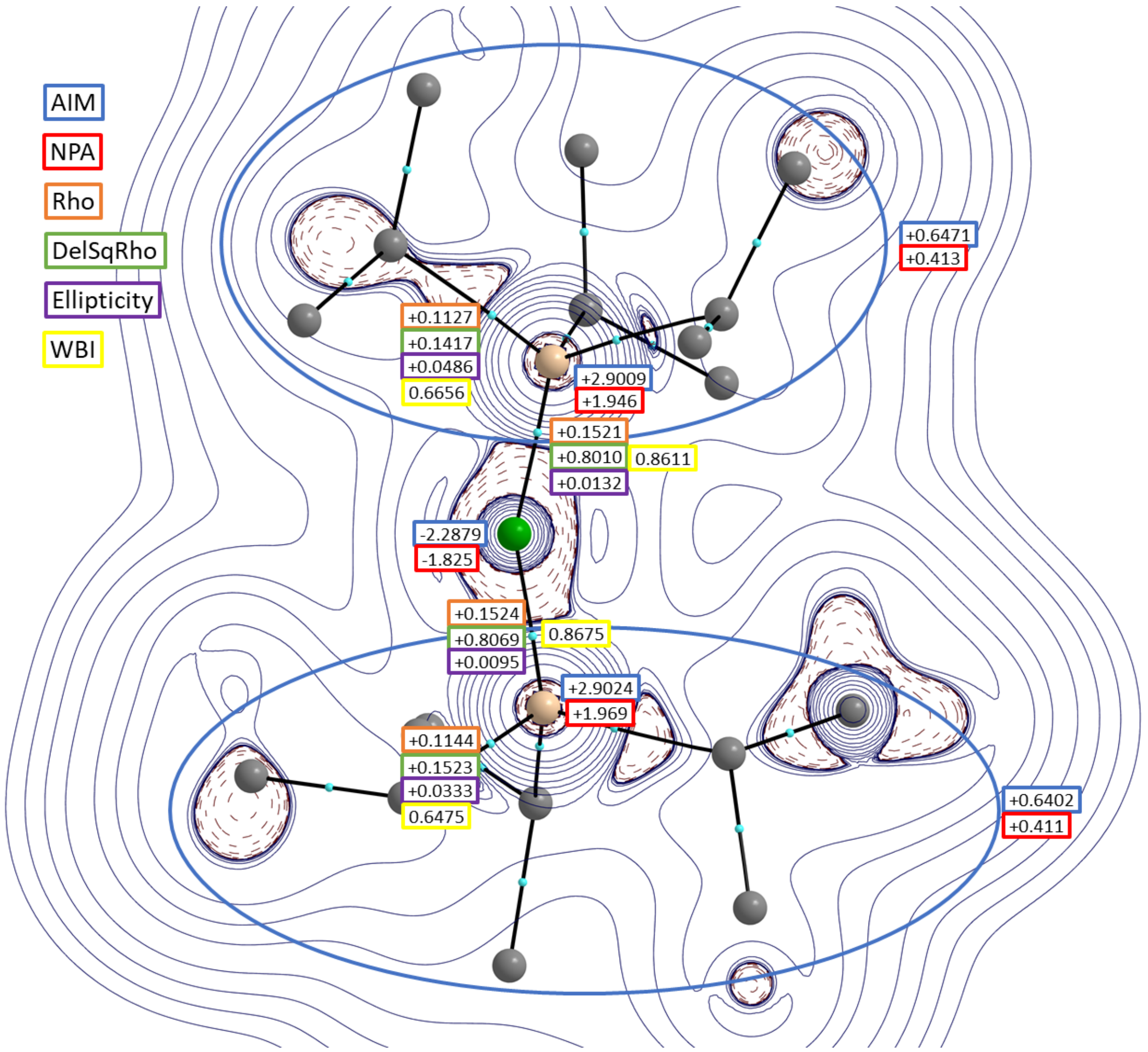

Figure S29: Atoms-In-Molecules: Contour plots of the Laplacian, $\nabla^2\rho(r)$, of free $(^{iPr}N)^-$ (calculated with dispersion) through the plane formed by the N and Si atoms. Light-blue dots: bond critical point (BCP); red boxes: NPA charges; blue boxes: AIM charges; orange boxes: electron densities in the BCPs; green boxes: Laplacian $\nabla^2\rho$ in BCPs; purple boxes: bond ellipticity. Red dashed lines indicate areas of charge concentration, while blue solid lines show areas of charge depletion.

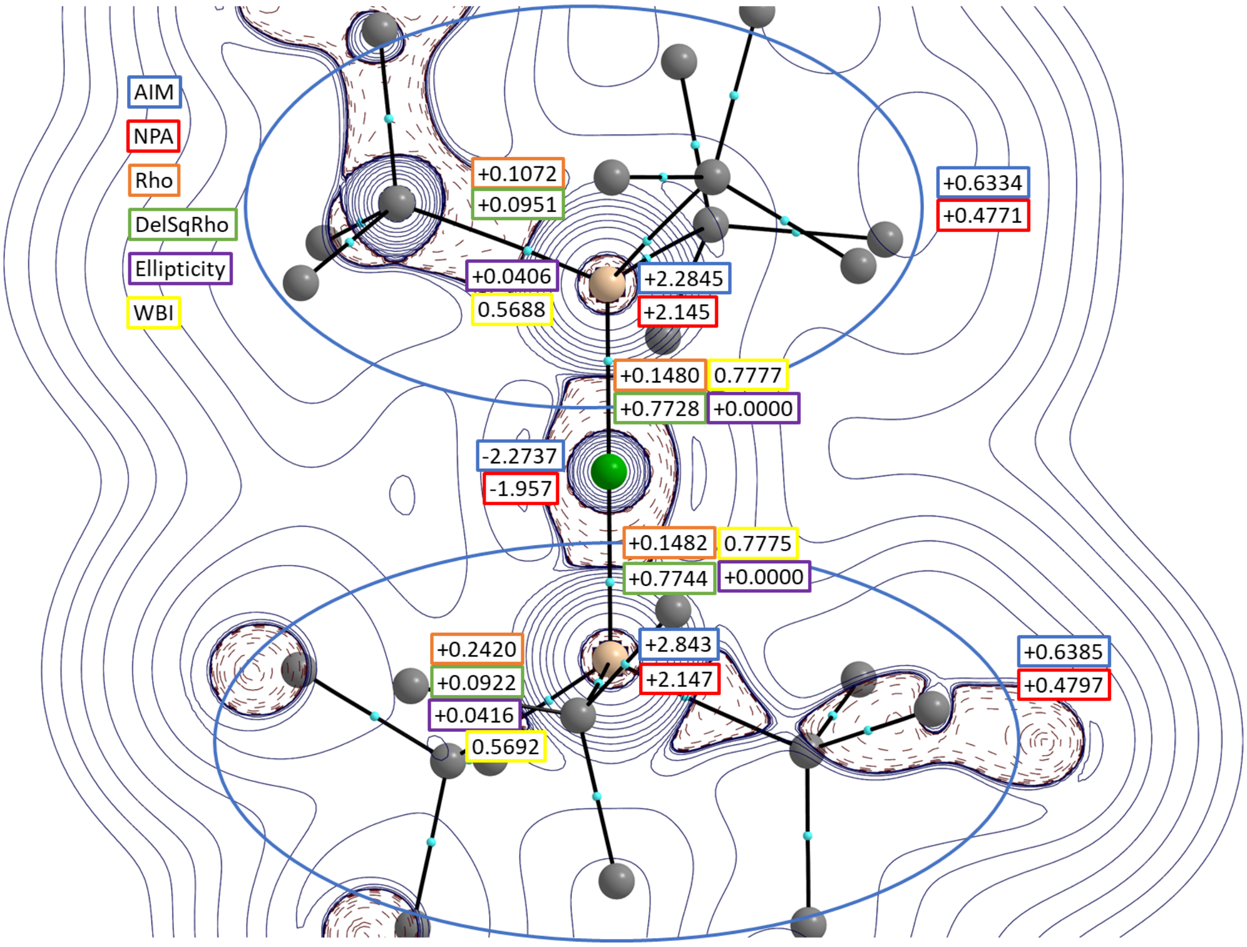

Figure S30: Atoms-In-Molecules: Contour plots of the Laplacian, $\nabla^2\rho(r)$, of free $(^{tBu}N)^-$ (calculated with dispersion) through the plane formed by the N and Si atoms. Light-blue dots: bond critical point (BCP); red boxes: NPA charges; blue boxes: AIM charges; orange boxes: electron densities in the BCPs; green boxes: Laplacian $\nabla^2\rho$ in BCPs; purple boxes: bond ellipticity. Red dashed lines indicate areas of charge concentration, while blue solid lines show areas of charge depletion. H atoms omitted for clarity.

Buried Volume Determination & Steric Maps

Molecular graphics and analyses performed with UCSF ChimeraX version 1.6.1, developed by the Resource for Biocomputing, Visualization, and Informatics at the University of California, San Francisco, with support from National Institutes of Health R01-GM129325 and the Office of Cyber Infrastructure and Computational Biology, National Institute of Allergy and Infectious Diseases.[16,17] Buried volumes and steric maps were determined using the SEQCROW plugin.[18] xyz files derived from crystallographic measurements or DFT calculation were used as input files. The calculations were run by using Bondi radii scaled by 1.17 for the atoms and sphere of R = 3.5 Å. The centre of the sphere was positioned at the metal centre and protons were included in the calculation.

Table S2: Buried volumes of various lithium amides.

	**Molecule**	**%VBur**
1	(*i*$Pr_3Si)_2NLi$, **X-ray** structure	63.0
2	(*t*$Bu_3Si)_2NLi$, **X-ray** structure	85.1
3	$(Me_3Si)_2NLi$, calculated with dispersion	45.4
4	$(Me_3Si)_2NLi$, calculated without dispersion	46.6
5	(*i*$Pr_3Si)_2NLi$, calculated with dispersion	72.7
6	(*i*$Pr_3Si)_2NLi$, calculated without dispersion	74.8
7	(*t*$Bu_3Si)_2NLi$, calculated with dispersion	86.6
8	(*t*$Bu_3Si)_2NLi$, calculated without dispersion	85.8

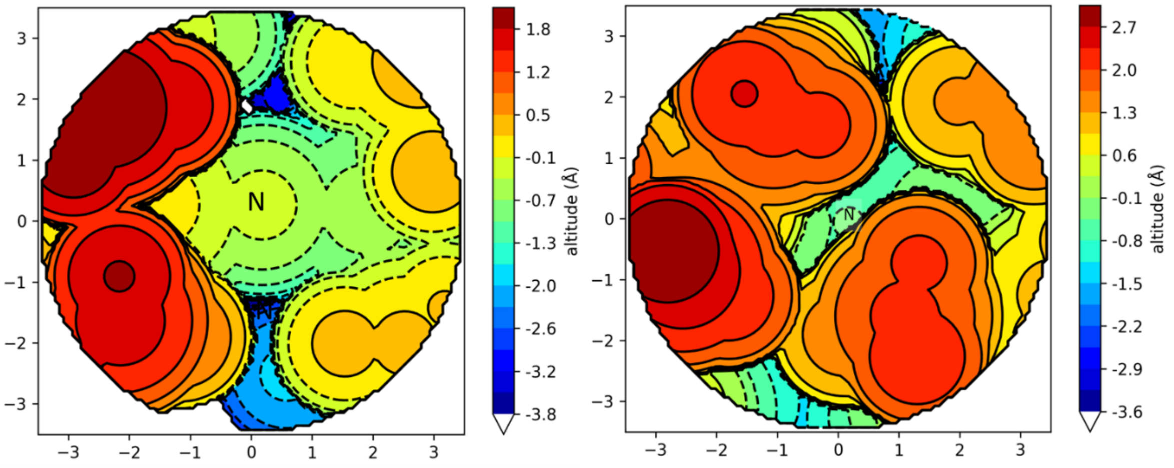

(*i*$Pr_3Si)_2NLi$
63.0 %VBur

(*t*$Bu_3Si)_2NLi$
85.1 %VBur

Figure S31: steric maps of (*i*$Pr_3Si)_2NLi$ (Table S2, entry 1) and (*t*$Bu_3Si)_2NLi$ (Table S1, entry 2).

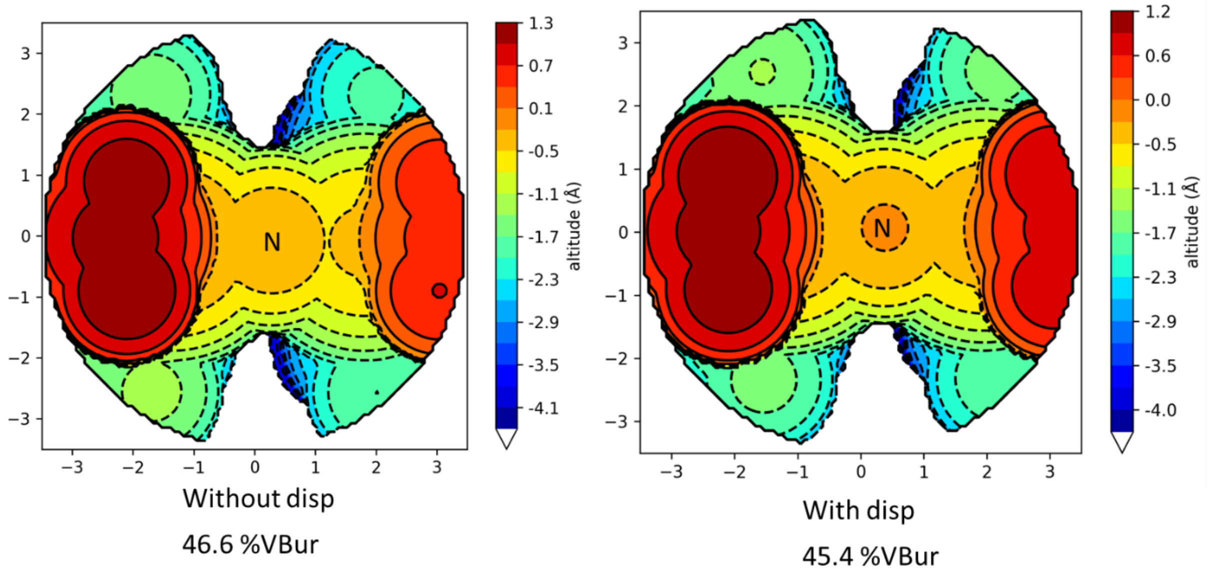

Figure S32: steric maps of calculated $(Me_3Si)_2NLi$ monomers without dispersion (left; Table S2, entry 4) and with dispersion (right; Table S2, entry 3).

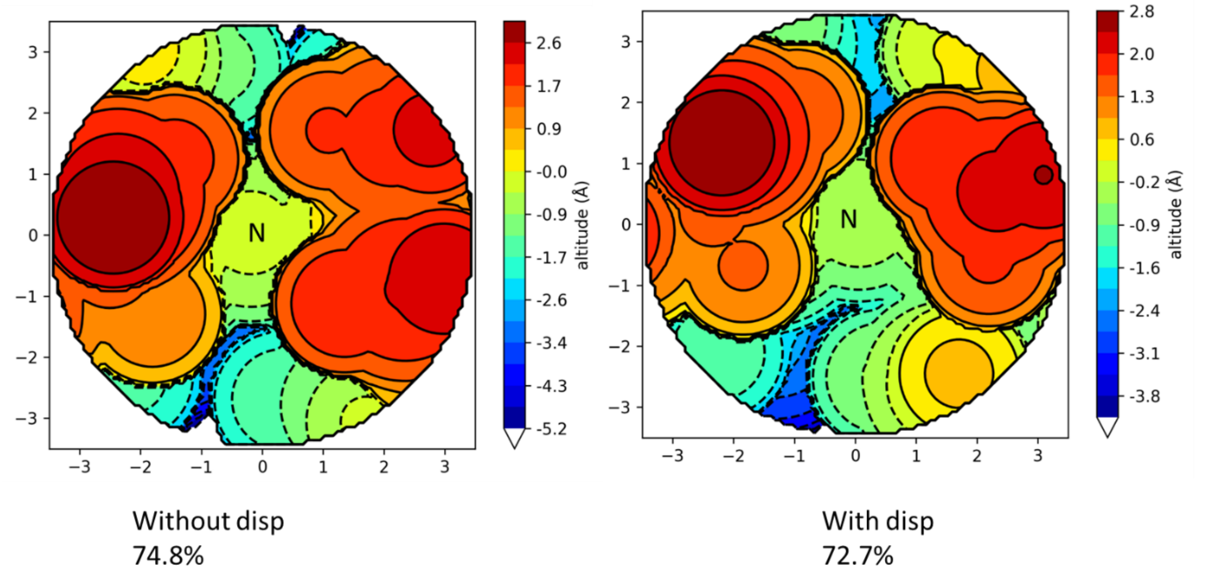

Figure S33: steric maps of calculated $(\textit{i}Pr_3Si)_2NLi$ without dispersion (left; Table S2, entry 6) and with dispersion (right; Table S2, entry 5).

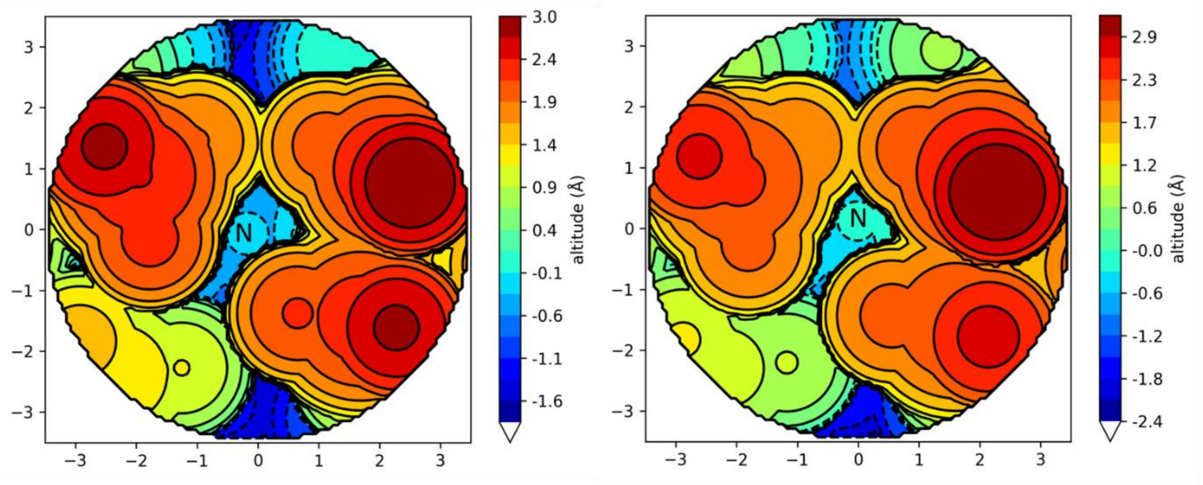

Without Disp
85.8%

With disp
86.6%

Figure S34: steric maps of calculated (tBu$_3$Si)$_2$NLi without dispersion (left; Table S2, entry 8) and with dispersion (right; Table S2, entry 7).

XRD Data

Suitable single crystals of all compounds were embedded in protective perfluoropolyalkyether oil (viscosity 1800 cSt; ABCR GmbH) on a microscope slide and a single specimen was selected and subsequently transferred to the cold nitrogen gas stream of the diffractometer.

The intensity data was collected at 100 K using MoK_α radiation (λ = 0.71073 Å; compounds (^{tBu}N)Cs (toluene)$_3$], (^{iPr}N)Cs) or CuK_α radiation (λ = 1.54184 Å; all other compounds) on an Agilent SuperNova dual radiation diffractometer with microfocus X-ray sources and mirror optics. The measured data were processed with the CrysAlisPro software package.[19] Data were corrected for Lorentz and polarization effects, and an empirical absorption correction using spherical harmonics as well as a numerical absorption correction based on Gaussian integration over a multifaceted crystal model were applied. Using Olex2,[19] the structures were solved by dual-space methods (SHELXT)[21] and refined by full-matrix least-squares procedures on F^2 using SHELXL.[22] All non-hydrogen atoms were refined with anisotropic displacement parameters. Most H-atoms were placed in geometrically calculated positions and refined by using a riding model where each H-atom was assigned a fixed isotropic displacement parameter with a value equal to 1.2U_{eq} (CH or CH_2) or 1.5U_{eq} (CH_3) of its parent C-atom.

In case of ^{tBu}NH, the N atoms are not located on special positions as indicated by the given model, but these atoms are severely disordered about those positions (not modeled) and likely occupy a large number of slightly different orientations. Consequently, the NH protons were also not included in the final model.

Compound [K^+ (18-crown-6)(THF)$_2$] [*t*BuN$^-$] contains 1.5 cations and 1.5 anions per asymmetric unit. The half of the anion as well as the three THF ligands are disordered. This disorder was modeled with the help of similarity restraints (SADI, SIMU) and rigid bond restraints (RIGU).[23] The relative occupancies of the two alternative orientations in each case were refined to 0.514(2)/0.486(2) (anion), 0.570(11)/0.430(11) (THF 1), 0.778(8)/0.222(8) (THF 2) and 0.785(7)/0.215(7) (THF 3), respectively.

In case of compound (^{tBu}N)Cs, the Cs atom is disordered about a 2-fold axis and was treated accordingly (Part -1, occupancy 0.5).

The same treatment was used for the four disordered THF moieties in the structure of [ISr$^+$·(THF)$_6$][^{tBu}N$^-$]. Here, site occupancy factors of 0.838(11)/0.162(11) (THF ligand 1), 0.693(14)/0.307(14) (THF ligand 2), 0.74(2)/0.26(2) (co-crystalized THF 1) and 0.551(16)/0.449(16) (co-crystalized THF 2) were observed after refinement.

The crystal of [(BDI)Mg$^+$·(THF)$_{1.26}$(Et_2O)$_{0.7}$][^{tBu}N$^-$] was a non-merohedral twin. The fractional contributions of the two twin domains were refined to 0.561(1) and 0.439(1). The structure is also

substantially disordered. The anion as well as 3 out of 4 *i*Pr groups and one THF ligand were found to occupy two alternative orientations each. Additionally, substitutional disorder between Et_2O and THF was found for the second neutral ligand. The corresponding site occupancy factors are 0.564(2)/0.436(2) (anion + 2 x *i*Pr + THF) and 0.745(6)/0.255(6) (*i*Pr + Et_2O/THF), respectively. Similarity restraints (SADI, SIMU) and rigid bond restraints (RIGU)[23] were used during refinement. In order to obtain more reasonable C-C bond lengths, an additional DFIX restraint on the diethyl ether ligand was applied.

The crystal of (^{tBu}N)Li was a racemic twin. The fractional contributions of the two twin domains were refined to 0.526(18) and 0.474(18). The asymmetric unit contained four independent molecules. The hydrogen atoms of molecule 1 were placed in the positions indicated by a difference electron density map and their positions were refined together with an isotropic displacement parameter. Although this is possible for most of the hydrogen atoms of the other three molecules as well, minor amounts of (untreated) disorder in molecule 2 and 3 and the resolved disorder in molecule 4 led to unreasonable position of some the hydrogen atoms. Therefore, the hydrogen atoms in the molecules 2-4 were placed and refined using a riding model as described above. The observed disorder of one Si*t*Bu_3 moiety in molecule 4 was modeled with the help of similarity restraints (SADI) and rigid bond restraints (RIGU).[23] Additionally, a rather tight ISOR restraint was placed on one of the carbon atoms (C29A) of the less occupied orientation. The relative occupancies of the two alternative orientations were refined to 0.772(3) and 0.228(3).

Severe disorder also affects the crown ether ligands in [Li^+·(12-crown-4)$_2$] [$^{tBu}N^-$] In this case, similarity restraints (SADI, SIMU) were sufficient to build a satisfying disorder model. The relative occupancies of the two alternative orientations of those two ligands are 0.72(2)/0.28(2) and 0.858(3)/0.142(3), respectively.

The cation in [Li^+·(PMDTA)(THF)] [$^{tBu}N^-$] is also severely disordered. The use of similarity restraints (SADI, SIMU) and rigid bond restraints (RIGU)[23] was required during refinement. The site occupancy factors for the two alternative orientations of the Li(pmdta) moiety were refined to 0.871(2) and 0.129(2), while those for the THF ligand were found to be 0.532(11) and 0.468(11).

The crystal structure data has been deposited with the Cambridge Crystallographic Data Centre. CCDC 2321294-2321305 contain the supplementary crystallographic data for the complexes. This data can be obtained free of charge from The Cambridge Crystallographic Data Centre via www.ccdc.cam.ac.uk/data_request/cif.

Crystallographic and refinement data are summarized in Table S3–6.

Table S 3: Crystal data and structure refinement.

Compound	^{tBu}NH	[K^{+} (18-crown-6)(THF)$_2$] [^{tBu}N^{-}]	(^{tBu}N)Cs
Identification code	hasj200807a	hasj210730a	hasj201211b
Empirical formula	$C_{24}H_{54}NSi_2$ a)	$C_{44}H_{94}KNO_8Si_2$	$C_{45}H_{78}CsNSi_2$
Formula weight	412.86	860.48	822.17
Temperature/K	100.00(10)	100.0(3)	100.01(10)
Crystal system	triclinic	triclinic	monoclinic
Space group	P-1	P-1	P2$_1$/c
a/Å	8.6659(5)	10.7820(2)	20.2860(4)
b/Å	11.1071(6)	15.4196(3)	14.13261(17)
c/Å	14.7913(8)	23.6530(4)	17.3397(3)
α/°	76.376(5)	80.1846(15)	90
β/°	86.941(4)	78.8889(16)	113.967(2)
γ/°	72.404(5)	83.5830(15)	90
Volume/Å^3	1318.67(13)	3789.54(12)	4542.57(15)
Z	2	3	4
ρ_{calc}g/cm^3	1.040	1.131	1.202
μ/mm^{-1}	1.260	1.739	0.897
F(000)	466.0	1428.0	1752.0
Crystal size/mm^3	0.273 × 0.22 × 0.167	0.439 × 0.326 × 0.26	0.552 × 0.365 × 0.334
Radiation	Cu Kα (λ = 1.54184)	Cu Kα (λ = 1.54184)	Mo Kα (λ = 0.71073)
2Θ range for data collection/°	8.582 to 144.45	5.836 to 144.532	4.394 to 59.056
Index ranges	-10 ≤ h ≤ 8, -10 ≤ k ≤ 13, -17 ≤ l ≤ 18	-13 ≤ h ≤ 8, -19 ≤ k ≤ 19, -28 ≤ l ≤ 28	-27 ≤ h ≤ 27, -18 ≤ k ≤ 18, -23 ≤ l ≤ 23
Reflections collected	9950	28075	79133
Independent reflections	5068 [R_{int} = 0.0213, R_{sigma} = 0.0280]	14567 [R_{int} = 0.0352, R_{sigma} = 0.0415]	11623 [R_{int} = 0.0363, R_{sigma} = 0.0263]
Data/restraints/parameters	5068/1546/382	14567/739/979	11623/0/463
Goodness-of-fit on F^2	1.048	1.022	1.057
Final R indexes [I>=2σ (I)]	R_1 = 0.0321, wR_2 = 0.0858	R_1 = 0.0420, wR_2 = 0.1123	R_1 = 0.0257, wR_2 = 0.0568
Final R indexes [all data]	R_1 = 0.0360, wR_2 = 0.0894	R_1 = 0.0486, wR_2 = 0.1200	R_1 = 0.0331, wR_2 = 0.0603
Largest diff. peak/hole / e Å^{-3}	0.34/-0.24	0.31/-0.42	0.78/-0.52
CCDC number	2321294	2321295	2321296

a) NH proton not included in final refinement und formula, see text

Table S 4: Crystal data and structure refinement (continued).

Compound	$(^{tBu}N)Cs$ (toluene)$_3$	[ISr$^+$ (THF)$_6$] [$^{tBu}N^-$]·2THF	[(BDI)Mg$^+$ (THF)$_{1.26}$(Et$_2$O)$_{0.74}$] [$^{tBu}N^-$]
Identification code	hasj211115a	hasj211029b	hasj231120b_twin1_hklf5
Empirical formula	$C_{24}H_{54}CsNSi_2$	$C_{56}H_{118}INO_8Si_2Sr$	$C_{61}H_{112.48}MgN_3O_2Si_2$
Formula weight	545.77	1204.21	1000.51
Temperature/K	100.0(6)	100.0(6)	99.97(11)
Crystal system	tetragonal	monoclinic	monoclinic
Space group	$P4_12_12$	$P2_1/n$	$P2_1/c$
a/Å	11.47464(7)	20.1783(2)	16.7968(4)
b/Å	11.47464(7)	12.98186(13)	16.8461(3)
c/Å	21.21699(17)	25.3950(3)	23.1147(5)
α/°	90	90	90
β/°	90	106.4553(12)	106.935(2)
γ/°	90	90	90
Volume/Å^3	2793.59(4)	6379.80(13)	6256.9(2)
Z	4	4	4
ρ_{calc}g/cm^3	1.298	1.254	1.062
μ/mm^{-1}	11.212	5.697	0.910
F(000)	1152.0	2576.0	2222.0
Crystal size/mm^3	0.216 × 0.078 × 0.075	0.389 × 0.274 × 0.234	0.418 × 0.399 × 0.104
Radiation	Cu Kα (λ = 1.54184)	Cu Kα (λ = 1.54184)	Cu Kα (λ = 1.54184)
2Θ range for data collection/°	8.762 to 145.192	6.59 to 145.448	7.604 to 145.252
Index ranges	-9 ≤ h ≤ 13, -14 ≤ k ≤ 14, -25 ≤ l ≤ 26	-24 ≤ h ≤ 23, -9 ≤ k ≤ 16, -15 ≤ l ≤ 30	-20 ≤ h ≤ 20, -20 ≤ k ≤ 20, -28 ≤ l ≤ 27
Reflections collected	11155	23607	16924
Independent reflections	2712 [R_{int} = 0.0237, R_{sigma} = 0.0173]	12282 [R_{int} = 0.0278, R_{sigma} = 0.0351]	16924 [R_{int} = 0.072, R_{sigma} = 0.0270]
Data/restraints/parameters	2712/0/142	12282/270/725	16924/924/1048
Goodness-of-fit on F^2	1.049	1.057	1.128
Final R indexes [I>=2σ (I)]	R_1 = 0.0176, wR_2 = 0.0449	R_1 = 0.0268, wR_2 = 0.0670	R_1 = 0.0540, wR_2 = 0.1614
Final R indexes [all data]	R_1 = 0.0179, wR_2 = 0.0451	R_1 = 0.0289, wR_2 = 0.0687	R_1 = 0.0630, wR_2 = 0.1646
Largest diff. peak/hole / e Å^{-3}	0.26/-0.39	0.65/-0.44	0.40/-0.28
Flack parameter	-0.0101(19)	-	-
CCDC number	2321297	2321298	2321299

Table S 5: Crystal data and structure refinement (continued).

Compound	$(^{tBu}N)Li$	$[Li^+ (12\text{-crown-}4)_2]\ [^{tBu}N^-]$	$[Li^+ (PMDTA)(THF)]\ [^{tBu}N^-]$
Identification code	hasj230309b	hasj211125b	hasj211125a
Empirical formula	$C_{24}H_{54}LiNSi_2$	$C_{40}H_{86}LiNO_8Si_2$	$C_{37}H_{85}LiN_4OSi_2$
Formula weight	419.80	772.21	665.20
Temperature/K	100.00(10)	100.0(4)	100.0(6)
Crystal system	triclinic	monoclinic	monoclinic
Space group	P1	C2/c	$P2_1/c$
a/Å	11.12464(16)	20.1279(3)	9.03040(10)
b/Å	14.7718(2)	26.8869(4)	26.9733(3)
c/Å	17.2761(3)	17.1171(3)	17.9436(2)
α/°	86.9319(12)	90	90
β/°	72.3912(13)	90.741(2)	96.5480(10)
γ/°	76.3044(13)	90	90
Volume/$Å^3$	2628.52(7)	9262.6(3)	4342.18(8)
Z	4	8	4
$\rho_{calc}g/cm^3$	1.061	1.107	1.018
μ/mm^{-1}	1.265	1.054	0.951
F(000)	944.0	3424.0	1496.0
Crystal size/mm^3	0.3 × 0.198 × 0.163	0.529 × 0.275 × 0.178	0.339 × 0.275 × 0.239
Radiation	Cu Kα (λ = 1.54184)	Cu Kα (λ = 1.54184)	Cu Kα (λ = 1.54184)
2Θ range for data collection/°	5.368 to 152.54	6.574 to 144.674	5.942 to 151.534
Index ranges	$-13 \le h \le 13, -18 \le k \le 18, -21 \le l \le 21$	$-24 \le h \le 24, -31 \le k \le 22, -18 \le l \le 20$	$-9 \le h \le 11, -33 \le k \le 20, -21 \le l \le 22$
Reflections collected	60832	16992	15845
Independent reflections	20603 [R_{int} = 0.0268, R_{sigma} = 0.0283]	8855 [R_{int} = 0.0253, R_{sigma} = 0.0308]	8713 [R_{int} = 0.0171, R_{sigma} = 0.0231]
Data/restraints/parameters	20603/132/1388	8855/2200/706	8713/1551/570
Goodness-of-fit on F^2	1.042	1.091	1.041
Final R indexes [I>=2σ (I)]	R_1 = 0.0395, wR_2 = 0.1076	R_1 = 0.0562, wR_2 = 0.1518	R_1 = 0.0390, wR_2 = 0.1053
Final R indexes [all data]	R_1 = 0.0445, wR_2 = 0.1132	R_1 = 0.0620, wR_2 = 0.1566	R_1 = 0.0414, wR_2 = 0.1077
Largest diff. peak/hole / e $Å^{-3}$	0.41/-0.25	0.61/-0.27	0.54/-0.25
Flack parameter	0.474(18)		
CCDC number	2321300	2321301	2321302

Table S 6: Crystal data and structure refinement (continued).

Compound	(^{iPr}N)Li	(^{iPr}N)Li (toluene)	(^{iPr}N)Cs
Identification code	hasj230729a	hasj220322c	hasj211027d
Empirical formula	$C_{18}H_{42}LiNSi_2$	$C_{25}H_{50}LiNSi_2$	$C_{18}H_{42}CsNSi_2$
Formula weight	335.64	427.78	461.61
Temperature/K	99.99(10)	100.0(2)	100.0(6)
Crystal system	monoclinic	monoclinic	orthorhombic
Space group	$P2_1/c$	C2/c	Pnna
a/Å	10.0842(2)	37.2445(4)	14.3530(4)
b/Å	16.5825(3)	8.91795(8)	17.6841(7)
c/Å	13.3677(2)	16.82946(17)	8.9669(3)
α/°	90	90	90
β/°	104.184(2)	102.8939(10)	90
γ/°	90	90	90
Volume/Å^3	2167.22(7)	5448.87(9)	2275.98(13)
Z	4	8	4
ρ_{calc}g/cm^3	1.029	1.043	1.347
μ/mm^{-1}	1.433	1.233	1.732
F(000)	752.0	1904.0	960.0
Crystal size/mm^3	0.303 × 0.283 × 0.149	0.337 × 0.221 × 0.127	0.303 × 0.235 × 0.136
Radiation	Cu Kα (λ = 1.54184)	Cu Kα (λ = 1.54184)	Mo Kα (λ = 0.71073)
2Θ range for data collection/°	8.66 to 145.05	9.744 to 144.944	4.606 to 59.436
Index ranges	-12 ≤ h ≤ 10, -20 ≤ k ≤ 18, -12 ≤ l ≤ 16	-45 ≤ h ≤ 45, -10 ≤ k ≤ 11, -20 ≤ l ≤ 20	-18 ≤ h ≤ 19, -24 ≤ k ≤ 23, -10 ≤ l ≤ 12
Reflections collected	7518	29522	10789
Independent reflections	4142 [R_{int} = 0.0238, R_{sigma} = 0.0337]	5330 [R_{int} = 0.0190, R_{sigma} = 0.0117]	2888 [R_{int} = 0.0288, R_{sigma} = 0.0266]
Data/restraints/parameters	4142/0/211	5330/0/275	2888/0/107
Goodness-of-fit on F^2	1.031	1.024	1.048
Final R indexes [I>=2σ (I)]	R_1 = 0.0324, wR_2 = 0.0831	R_1 = 0.0296, wR_2 = 0.0764	R_1 = 0.0234, wR_2 = 0.0513
Final R indexes [all data]	R_1 = 0.0365, wR_2 = 0.0861	R_1 = 0.0304, wR_2 = 0.0771	R_1 = 0.0310, wR_2 = 0.0554
Largest diff. peak/hole / e Å^{-3}	0.34/-0.29	0.31/-0.25	0.49/-0.32
CCDC number	2321303	2321304	2321305

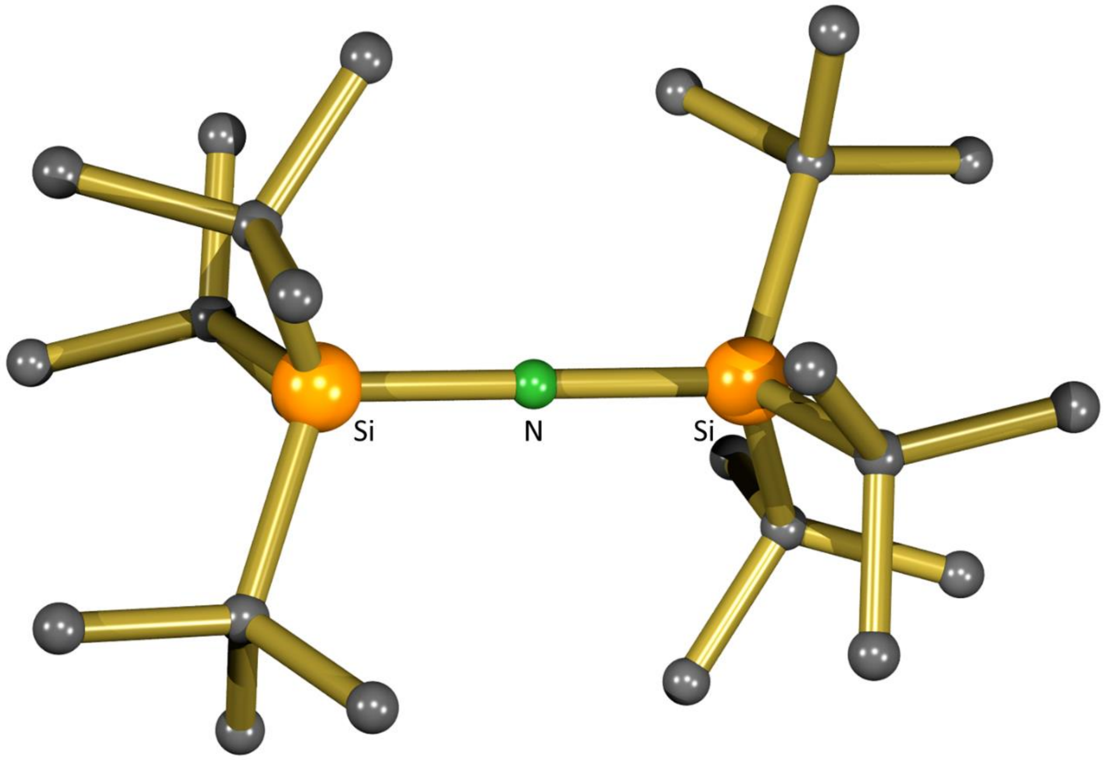

Figure S35: Molecular structure ^{tBu}NH. H atoms omitted.

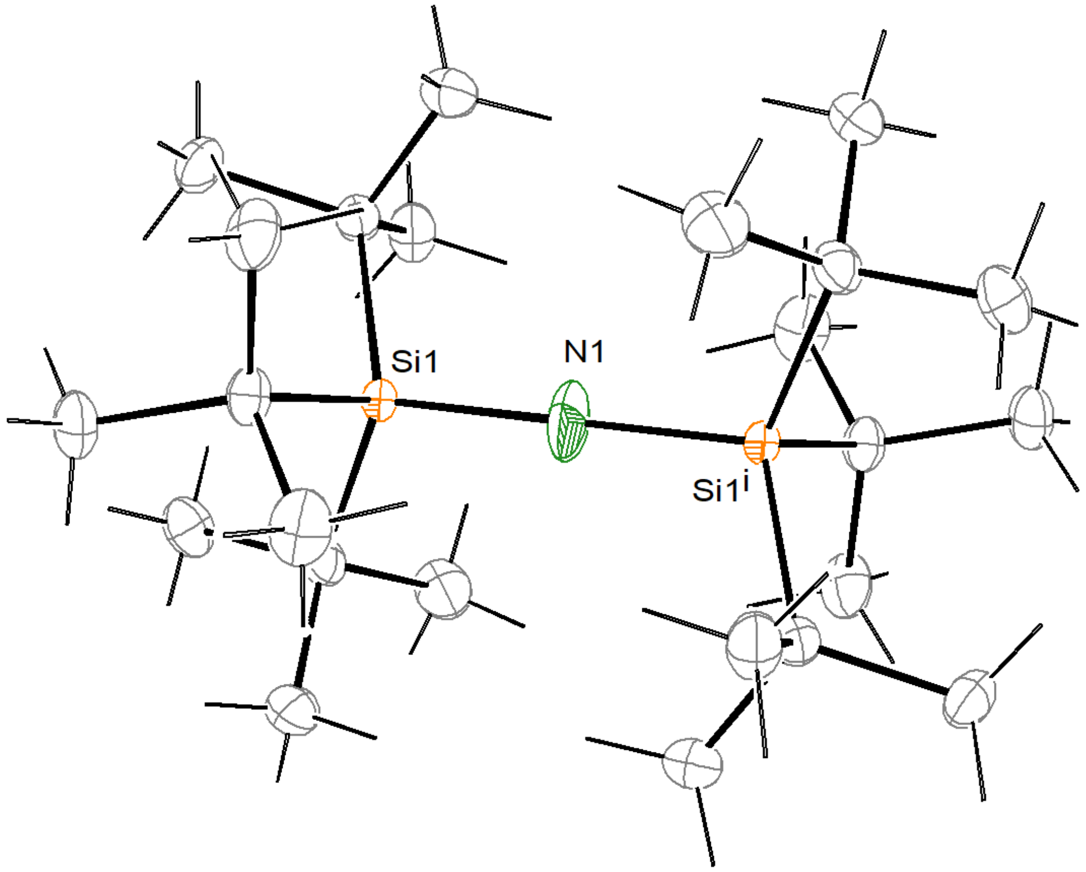

Figure S36: ORTEP plot of ^{tBu}NH. Ellipsoids are at 50% probability.

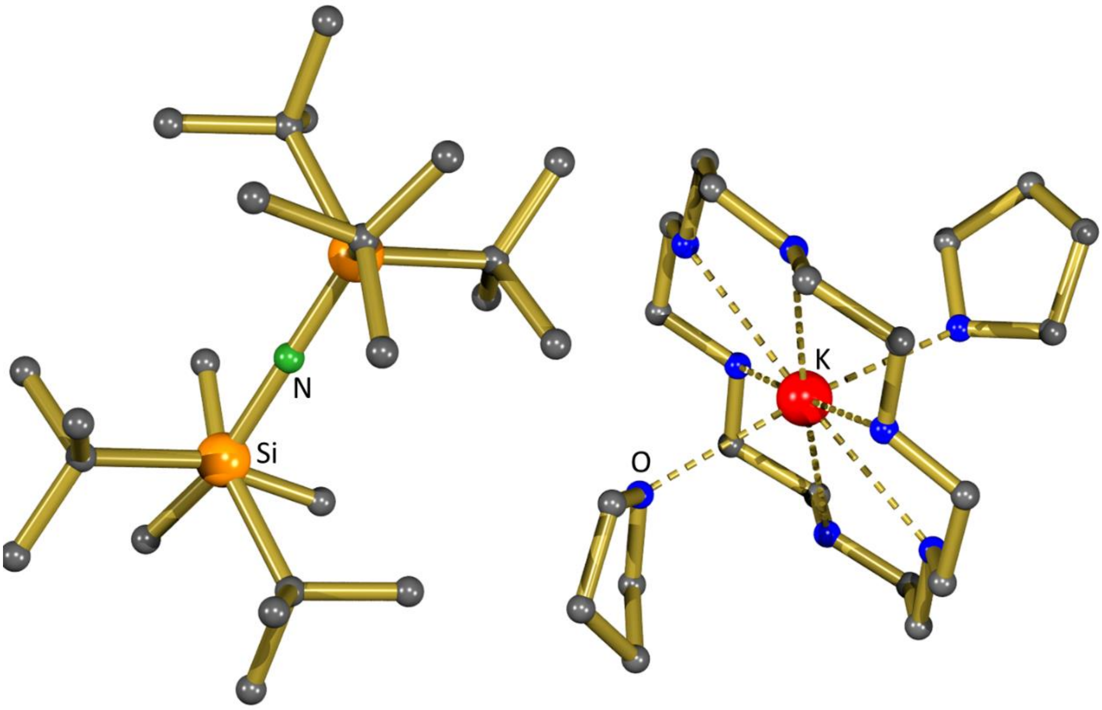

Figure S37: Molecular structure of [K⁺ (18-crown-6)(THF)₂] [^{tBu}N⁻]. H atoms omitted.

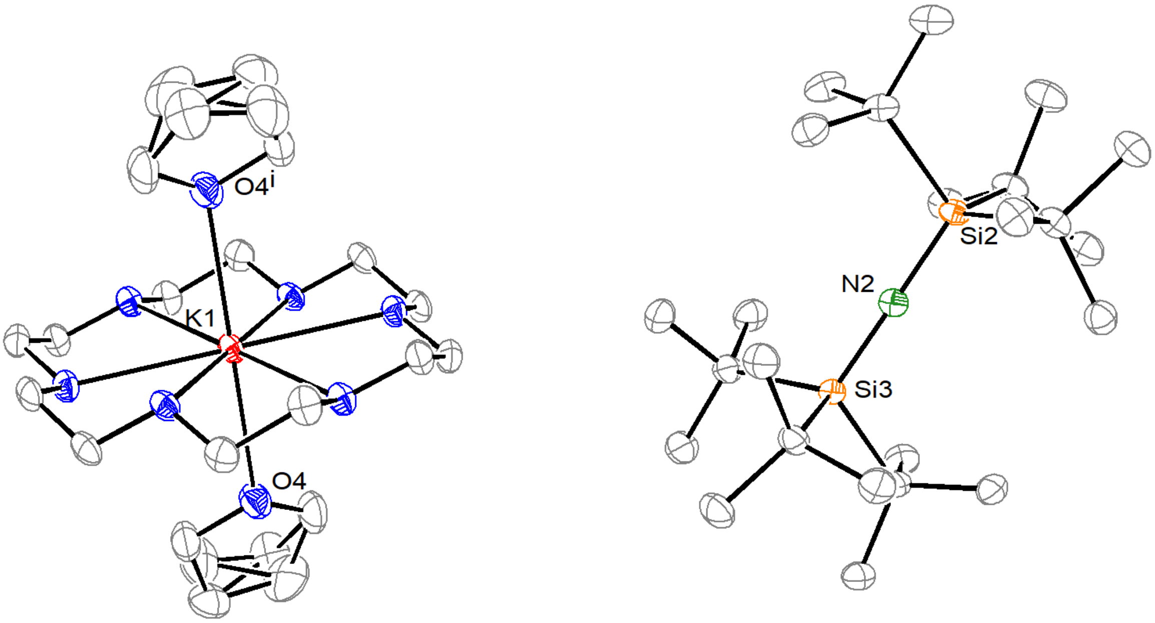

Figure S38: ORTEP plot of [K⁺ (18-crown-6)(THF)₂] [^{tBu}N⁻]. Ellipsoids are at 50% probability.

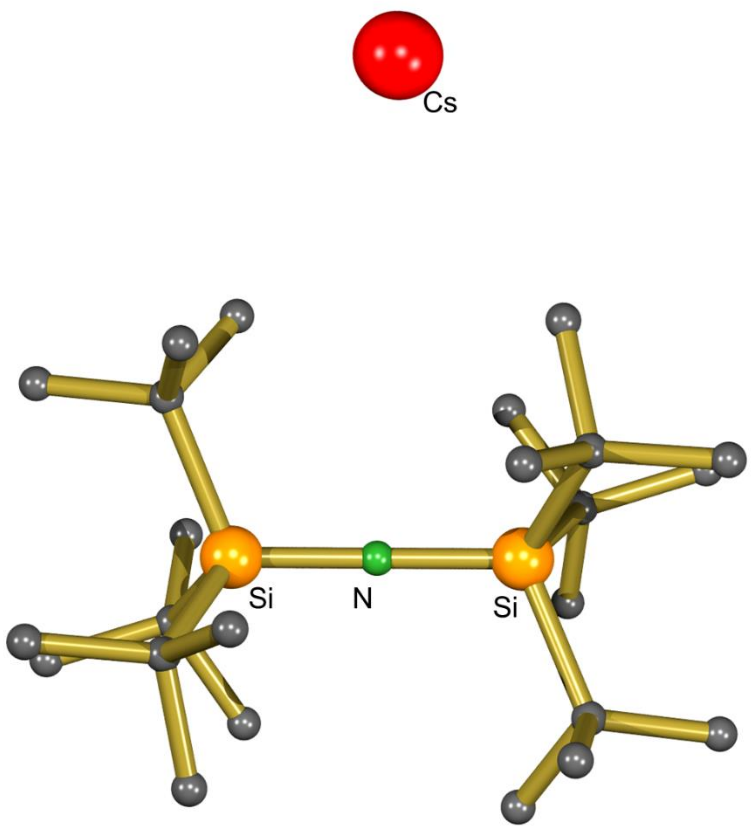

Figure S39: Molecular structure of (^{tBu}N)Cs. H atoms omitted.

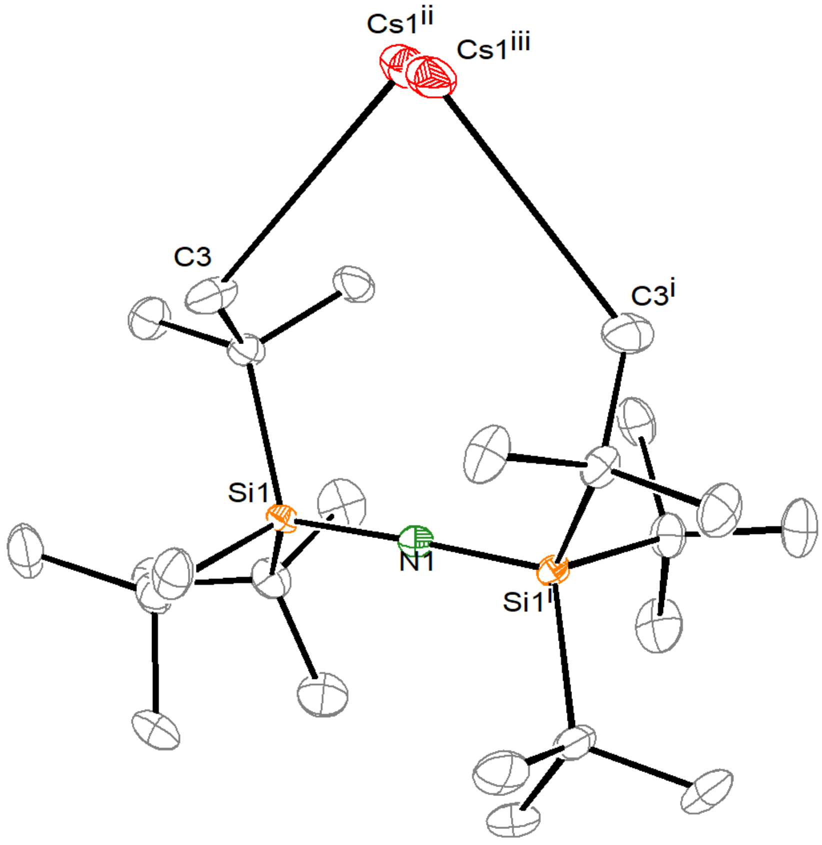

Figure S40: ORTEP plot of (^{tBu}N)Cs. Ellipsoids are at 50% probability.

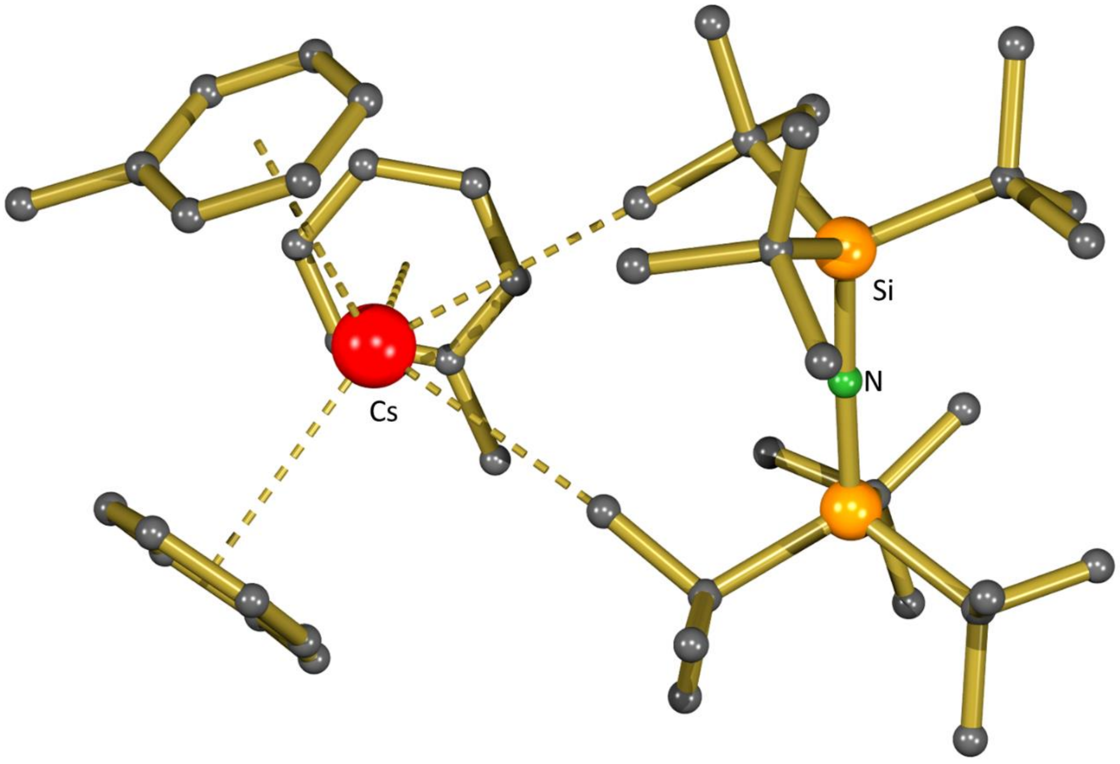

Figure S41: Molecular structure of (^{tBu}N)Cs (toluene)$_3$. H atoms omitted.

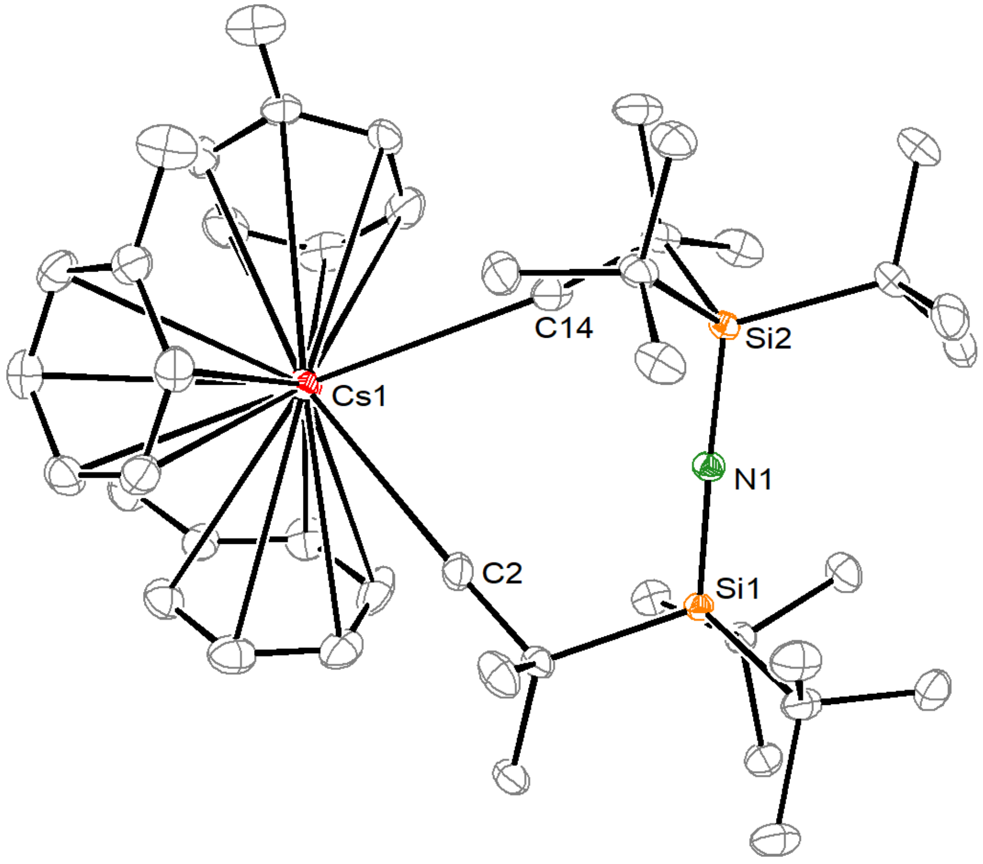

Figure S42: ORTEP plot of (^{tBu}N)Cs (toluene)$_3$. Ellipsoids are at 50% probability.

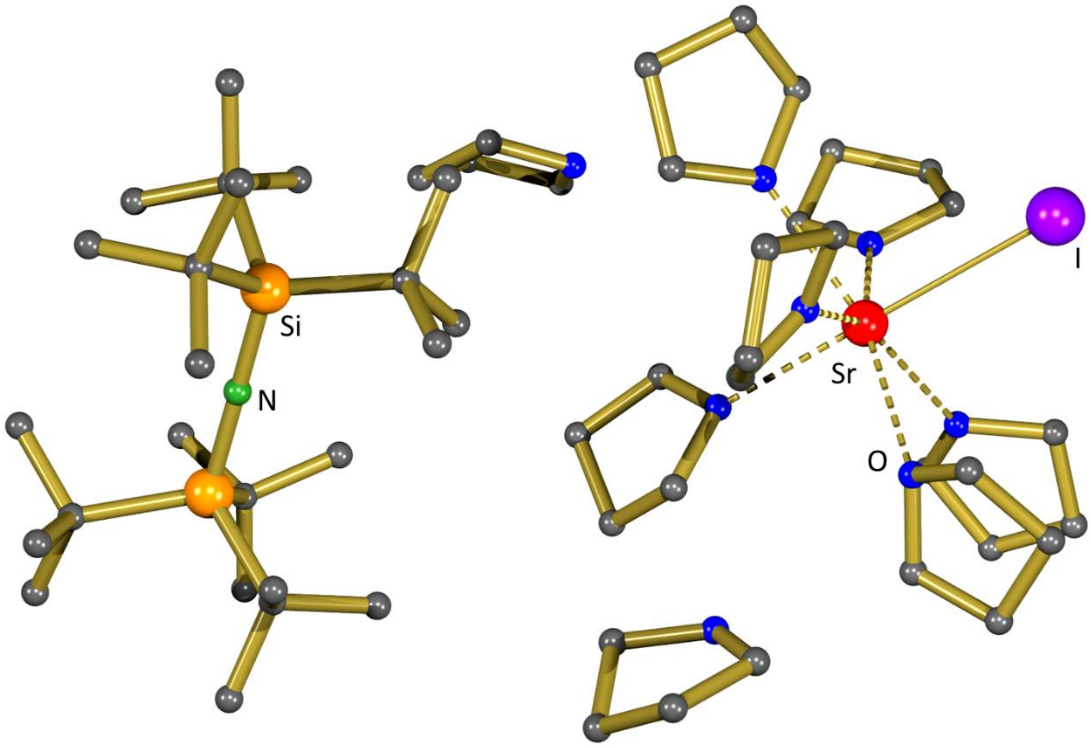

Figure S43: Molecular structure of [ISr^{+}·$(THF)_6$][$^{tBu}N^{-}$]·$(THF)_2$. H atoms omitted.

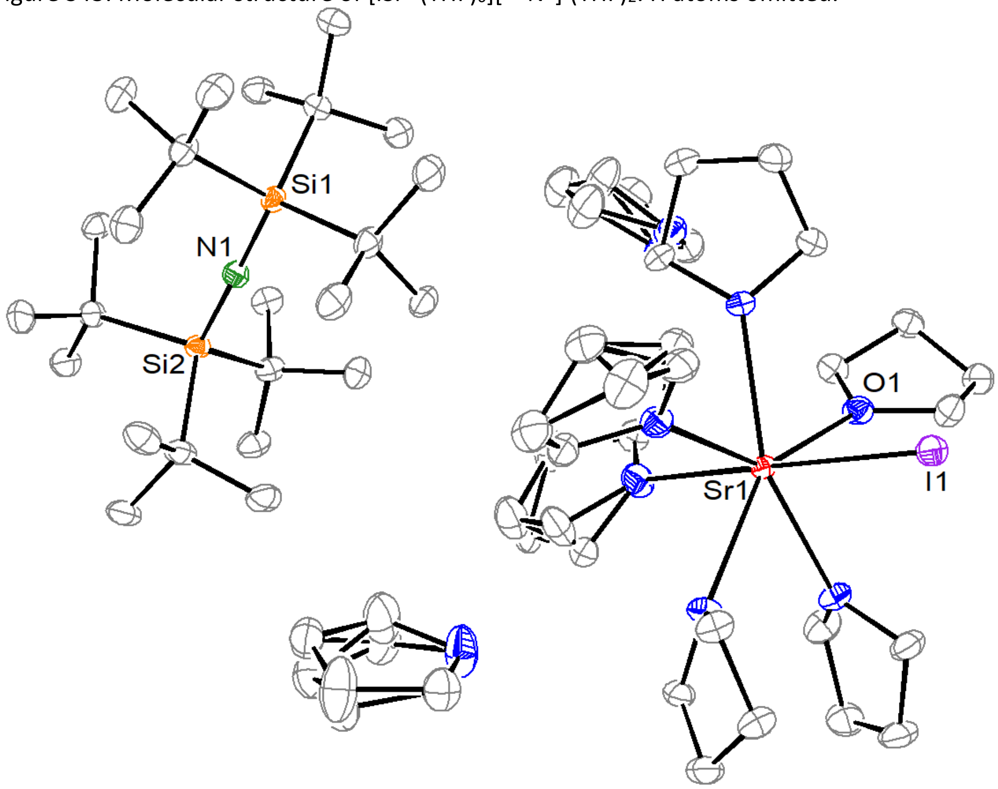

Figure S44: ORTEP plot of [ISr^{+}·$(THF)_6$][$^{tBu}N^{-}$]·$(THF)_2$. Ellipsoids are at 50% probability.

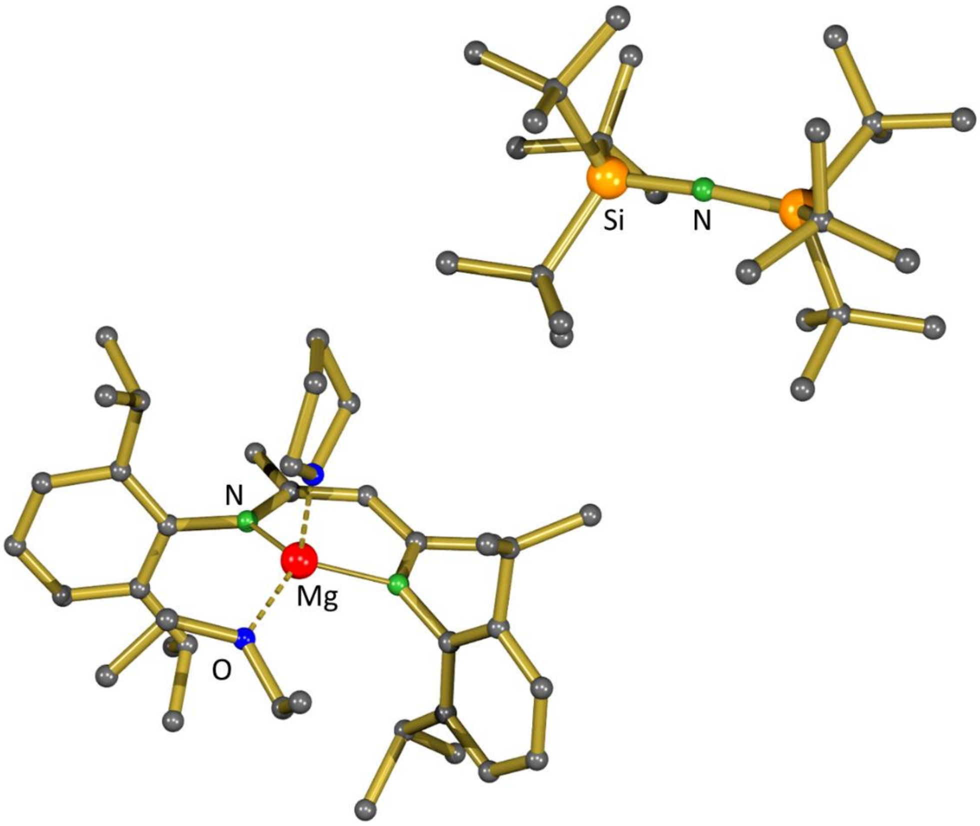

Figure S45: Molecular structure of [(BDI)Mg^{+}·(THF)(Et_2O)][$^{tBu}N^{-}$]. H atoms omitted.

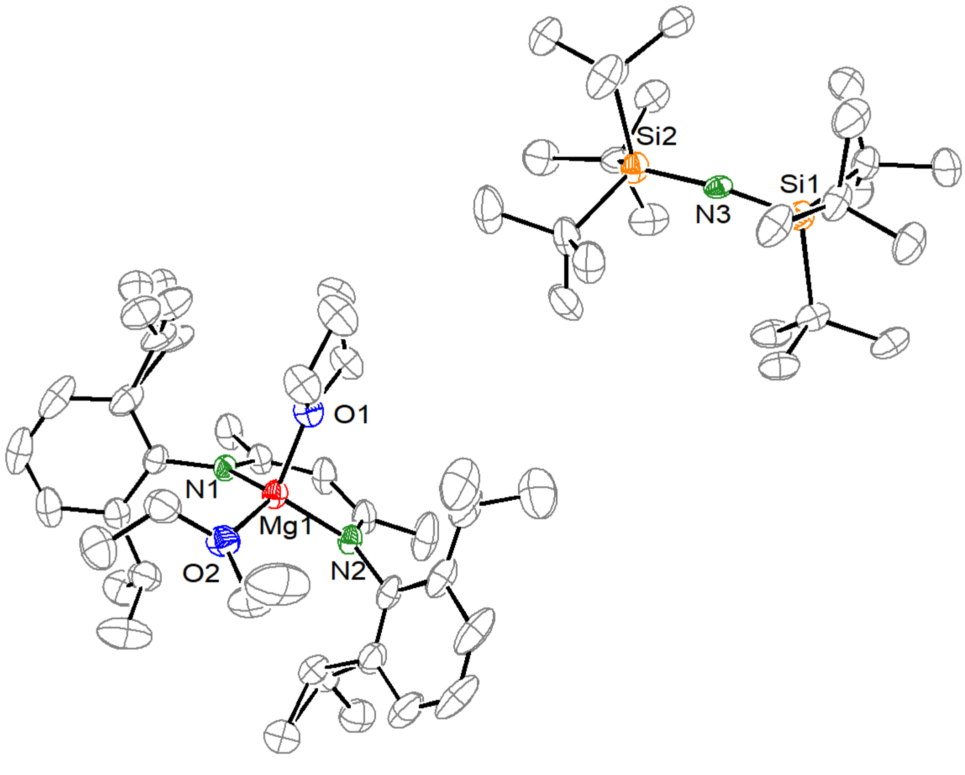

Figure S46: ORTEP plot of of [(BDI)Mg^{+}·(THF)(Et_2O)][$^{tBu}N^{-}$]. Ellipsoids are at 50% probability.

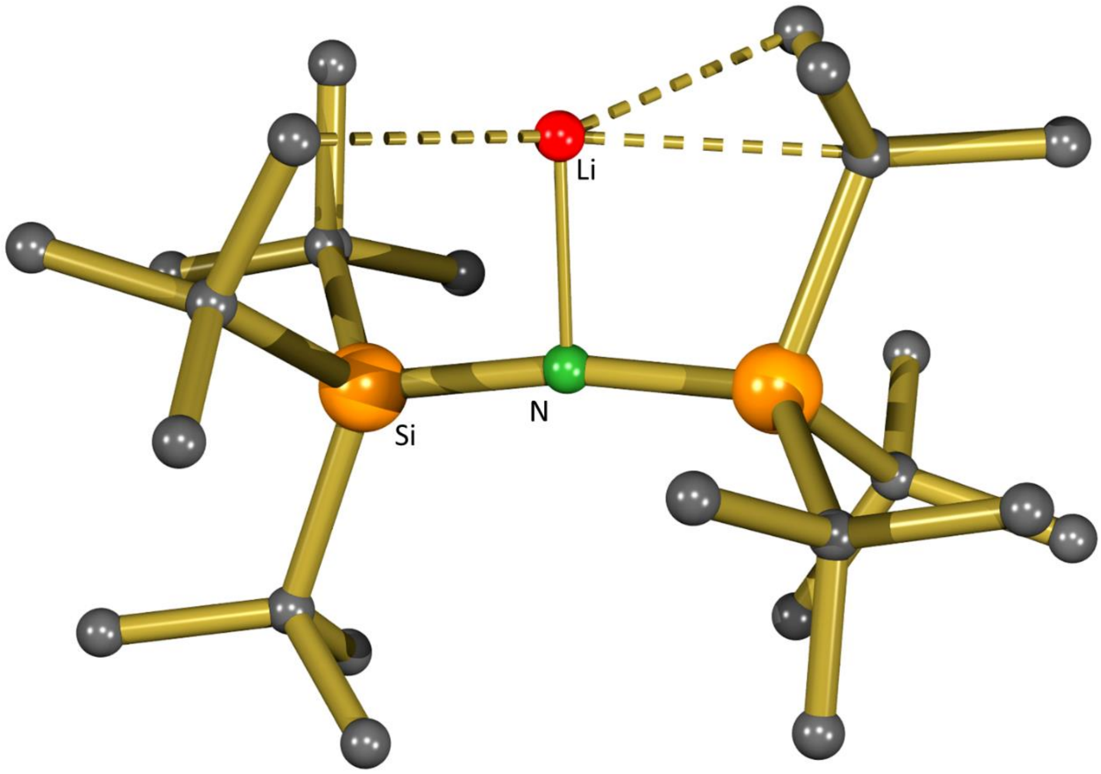

Figure S47: Molecular structure of (^{tBu}N)Li. H atoms omitted.

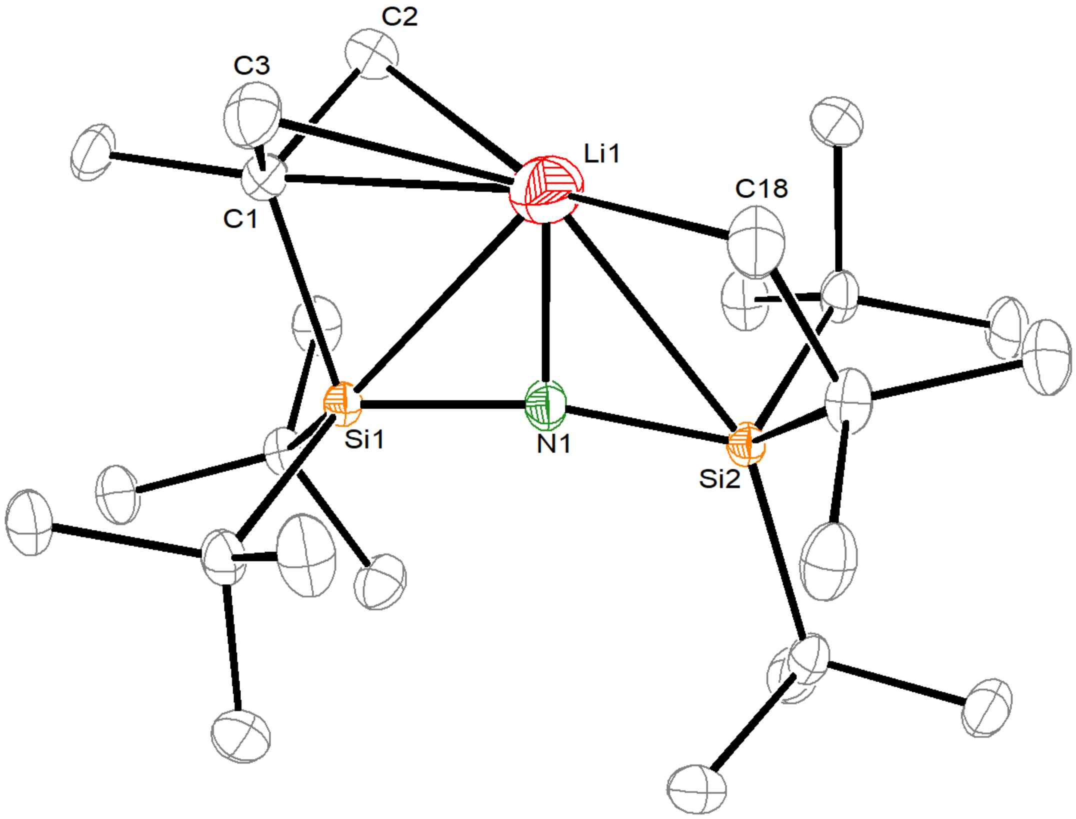

Figure S48: ORTEP plot of (^{tBu}N)Li. Ellipsoids are at 50% probability.

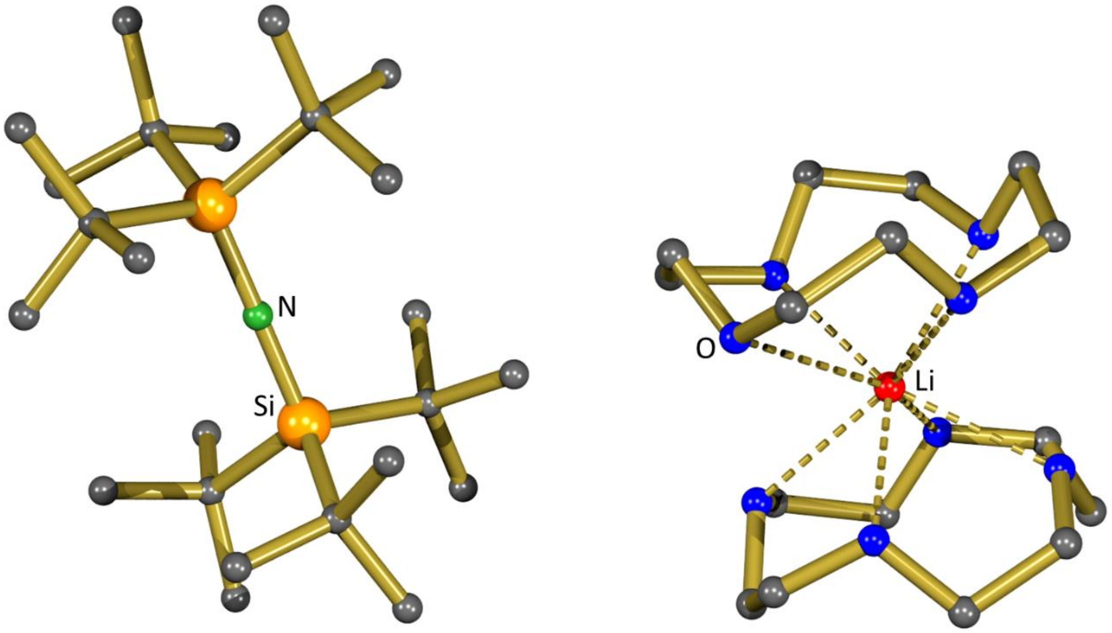

Figure S49: Molecular structure of $[Li^+\cdot(12\text{-crown-4})_2]$ $[{}^{tBu}N^-]$. H atoms omitted.

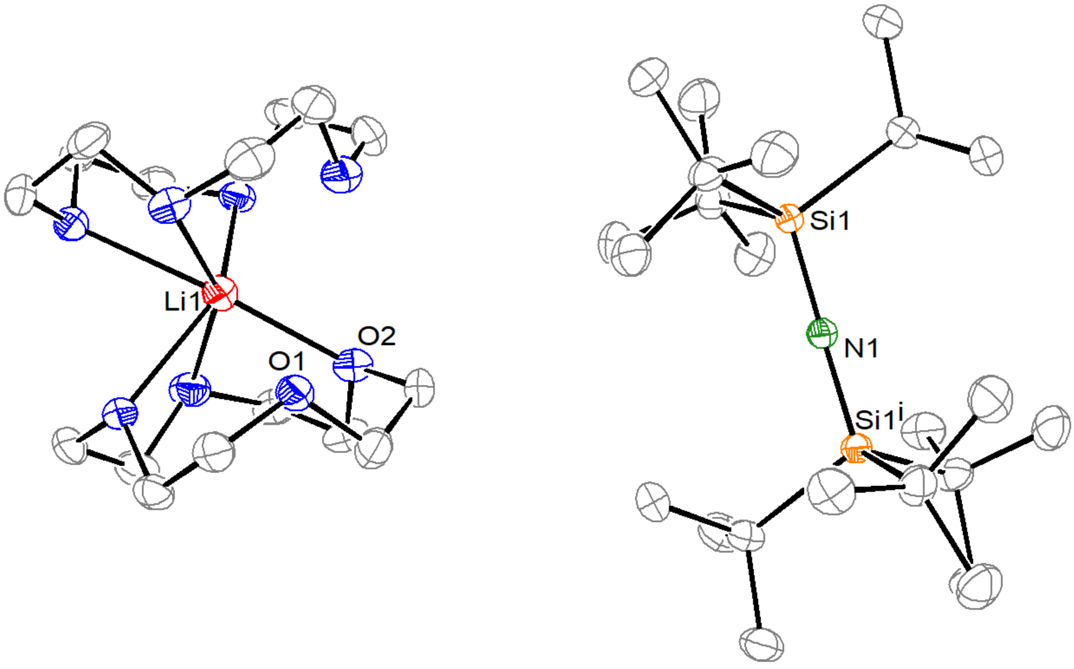

Figure S50: ORTEP plot of $[Li^+\cdot(12\text{-crown-4})_2]$ $[{}^{tBu}N^-]$. Ellipsoids are at 50% probability.

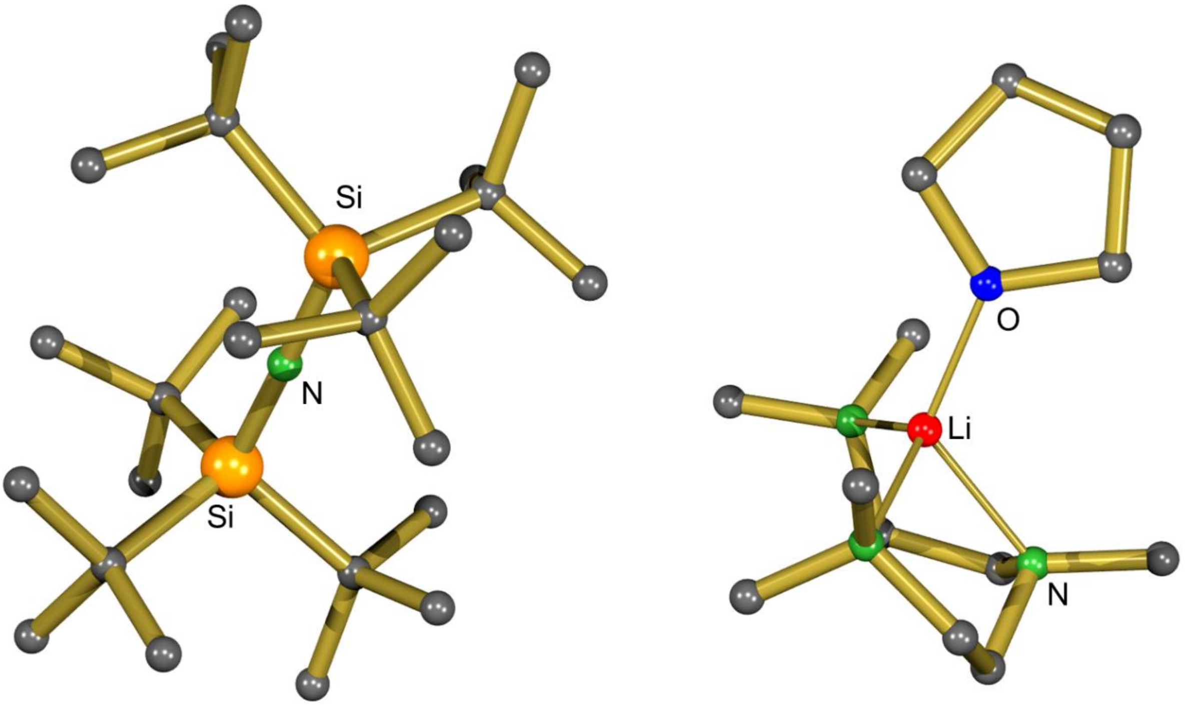

Figure S51: Molecular structure of [Li^{+}·(PMDTA)(THF)] [^{tBu}N^{-}]. H atoms omitted.

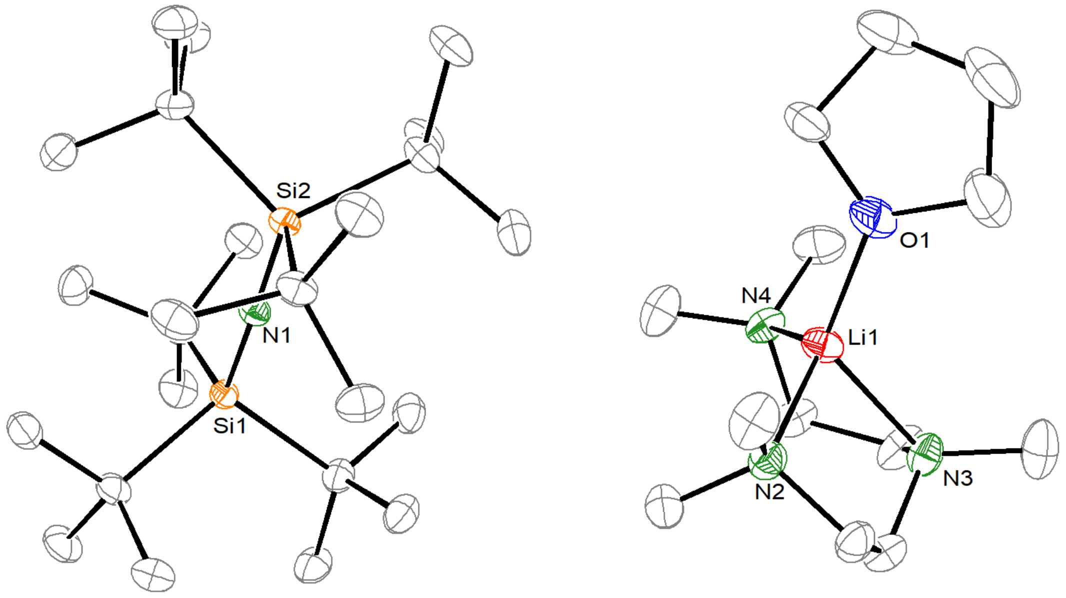

Figure S52: ORTEP plot of [Li^{+}·(PMDTA)(THF)] [^{tBu}N^{-}]. Ellipsoids are at 50% probability.

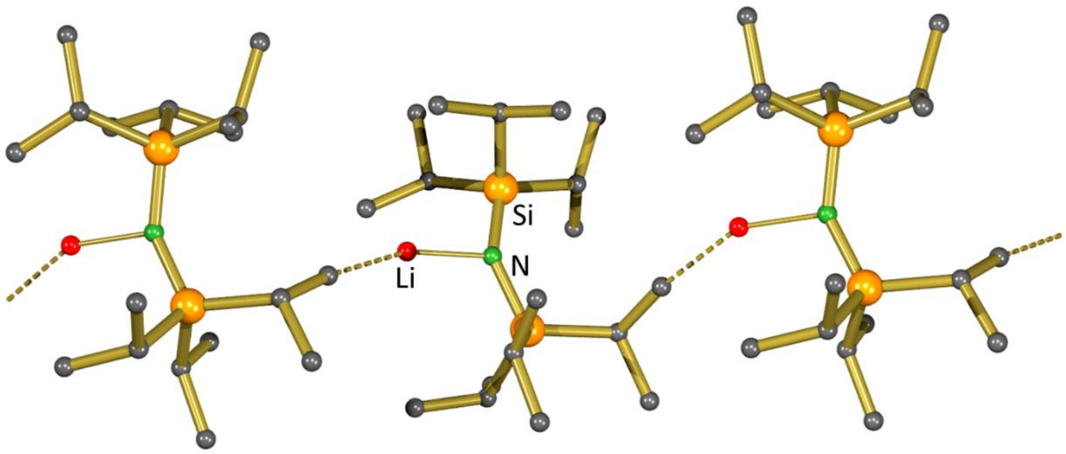

Figure S53: Molecular structure of (^{iPr}N)Li. H atoms omitted.

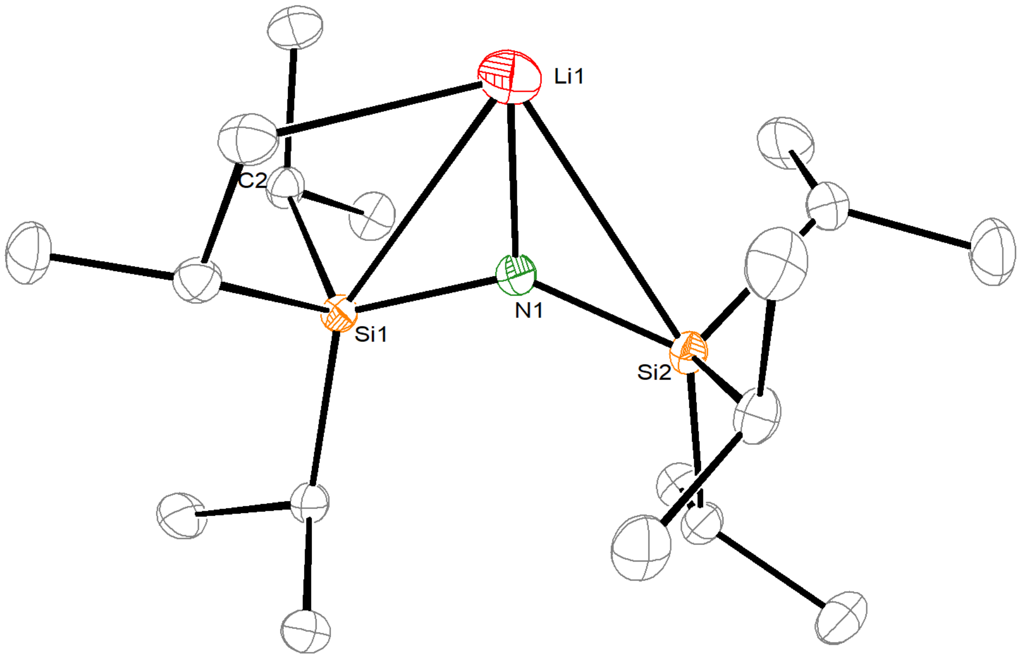

Figure S54: ORTEP plot of a (^{iPr}N)Li monomer. Ellipsoids are at 50% probability.

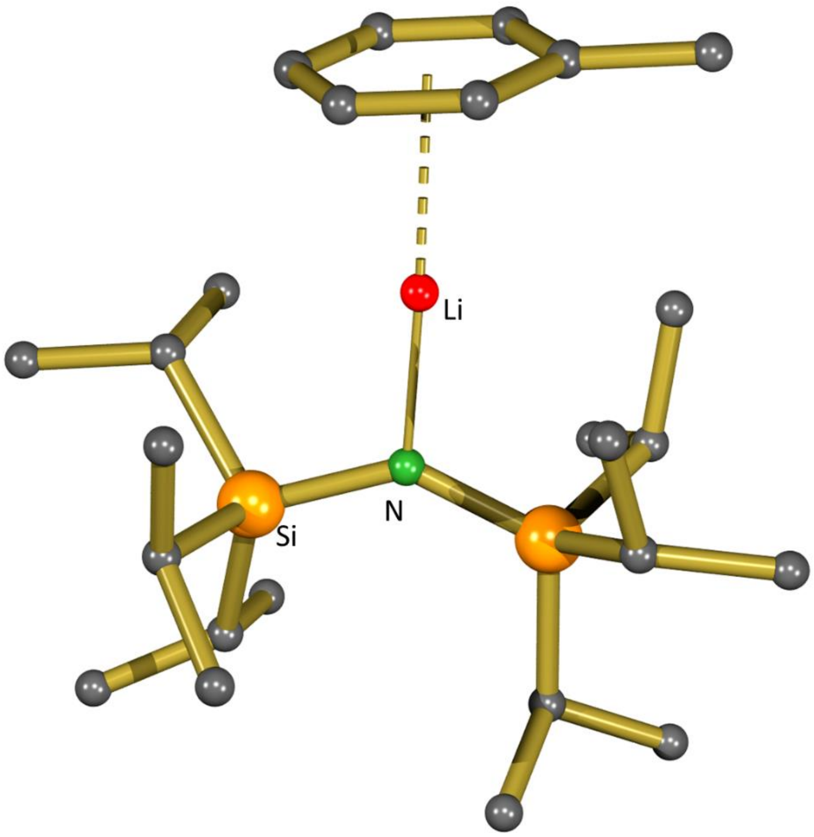

Figure S55: Molecular structure of (^{iPr}N)Li (toluene). H atoms omitted.

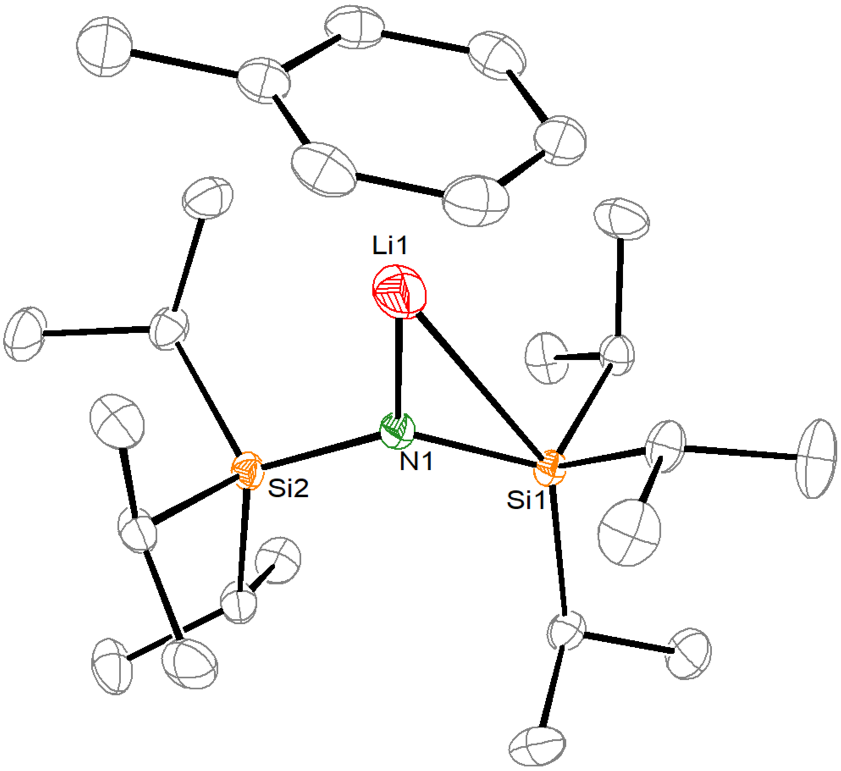

Figure S56: ORTEP plot of (^{iPr}N)Li (toluene). Ellipsoids are at 50% probability.

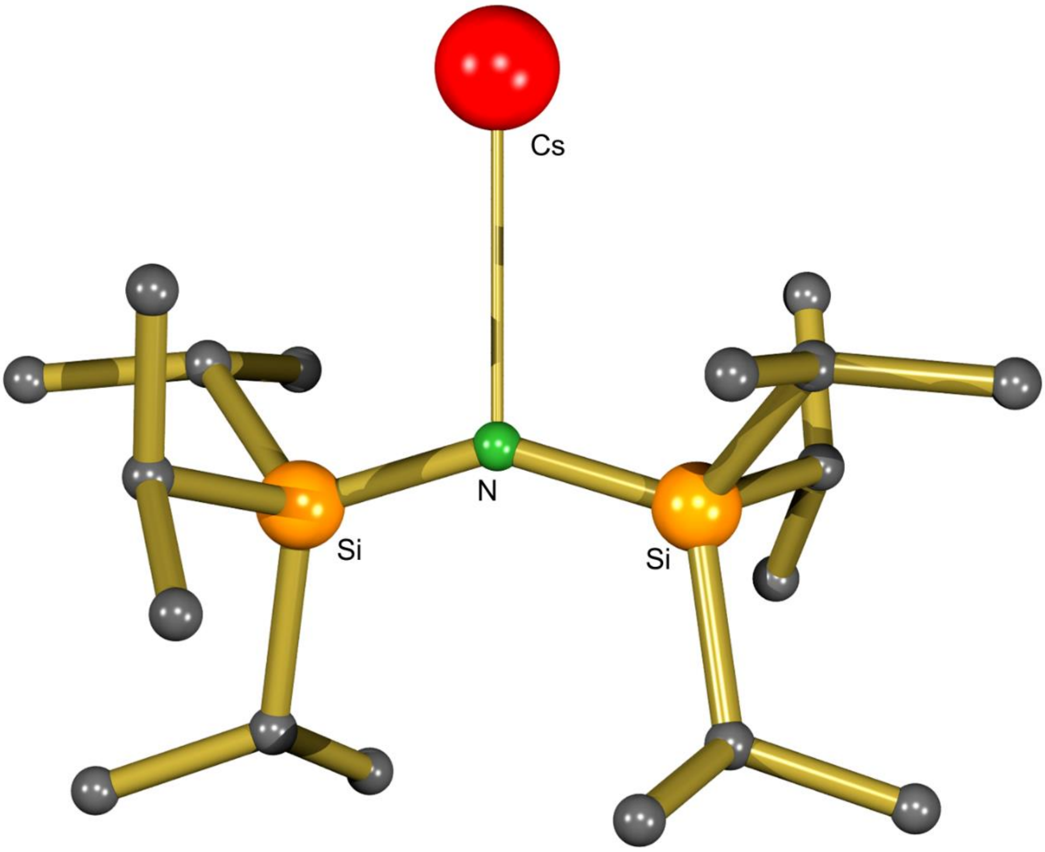

Figure S57: Molecular structure of (^{iPr}N)Cs. H atoms omitted.

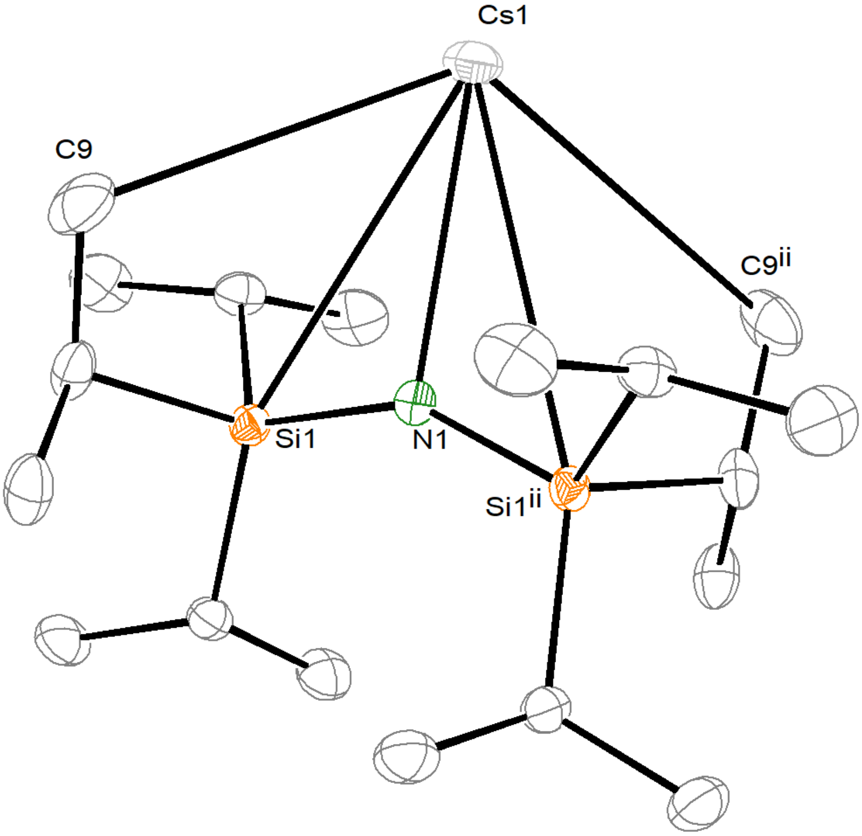

Figure S58: ORTEP plot of (^{iPr}N)Cs. Ellipsoids are at 50% probability.

XYZ Coordinates

28
$(Me_3Si)_2NLi$_tzvp
Si 1.609673 0.013278 0.000008
Si -1.552410 -0.047515 0.000015
N 0.011778 0.567933 0.000072
C 2.760896 1.521696 -0.001463
H 2.602049 2.144159 -0.889243
H 3.815319 1.230814 -0.001495
H 2.602524 2.145551 0.885431
C 2.054336 -1.017859 -1.518599
H 1.461400 -1.936095 -1.558509
H 3.109673 -1.307115 -1.515124
H 1.861500 -0.463310 -2.441244
C 2.055178 -1.015474 1.519989
H 1.862938 -0.459426 2.441857
H 3.110487 -1.304835 1.516359
H 1.462168 -1.933581 1.561729
C -2.689090 1.515511 0.000052
H -2.576658 2.145317 0.896010
H -3.739829 1.216491 0.000005
H -2.576611 2.145395 -0.895847
C -2.053087 -1.041710 -1.520901
H -1.859645 -0.483674 -2.440851
H -3.113256 -1.311709 -1.502698
H -1.477866 -1.970115 -1.576944
C -2.053156 -1.041841 1.520825
H -1.478010 -1.970302 1.576753
H -3.113345 -1.311757 1.502602
H -1.859658 -0.483926 2.440836
Li -0.475924 2.296658 0.000044

28
$(Me_3Si)_2NLi$_tzvp-disp
Si 1.581936 0.034840 -0.000035
Si -1.516182 -0.034445 0.000032
N 0.012911 0.660453 0.000044
C 2.803963 1.475557 -0.005181
H 2.668507 2.101354 -0.893520
H 3.841875 1.131950 -0.005236
H 2.670312 2.106216 0.880007
C 1.952645 -1.023688 -1.513491
H 1.316325 -1.912678 -1.531026
H 2.993380 -1.359937 -1.529130
H 1.762446 -0.465452 -2.434006
C 1.955917 -1.015354 1.518411
H 1.768505 -0.451737 2.436221
H 2.996471 -1.352204 1.533276
H 1.319003 -1.903752 1.542703
C -2.730416 1.459598 0.001139
H -2.647940 2.092122 0.897754
H -3.763103 1.104521 0.001305
H -2.648592 2.092958 -0.894958
C -1.948439 -1.051962 -1.519319
H -1.768066 -0.482868 -2.434687
H -2.993328 -1.375043 -1.514542
H -1.323580 -1.947747 -1.563954
C -1.947637 -1.053874 1.518322
H -1.322955 -1.949880 1.561236
H -2.992590 -1.376750 1.513833
H -1.766472 -0.486097 2.434346
Li -0.545774 2.354886 0.000277

64
$(\textit{i}Pr_3Si)_2NLi$_tzvp
Si 1.608896 -0.111936 -0.079163
Si -1.690414 -0.021448 0.038951
N -0.040248 0.209540 -0.118659
C 2.358366 1.353131 -1.133543
H 1.665108 1.384337 -1.995885
C 3.746913 1.177988 -1.751061
C 2.303419 2.717360 -0.425053
C 2.072254 -1.769179 -0.931148
C 1.162379 -2.903501 -0.451249
C 3.528148 -2.232923 -0.829884
H 1.848802 -1.597281 -1.994649
C 2.343456 -0.125269 1.697915
C 1.613875 0.803374 2.670256
C 3.854814 0.102990 1.800300
H 2.149116 -1.154461 2.035478
C -2.552182 1.690042 -0.358182
C -2.199283 2.805314 0.634118
H -3.616476 1.474128 -0.207143
C -2.447631 2.200873 -1.803026
C -2.429344 -1.324562 -1.164025
C -1.727187 -1.373582 -2.523168
C -3.946652 -1.250532 -1.359713
H -2.217285 -2.279866 -0.662560
C -2.214064 -0.435911 1.836940
C -1.629053 -1.773752 2.295888
C -3.702396 -0.363900 2.182774
H -1.707584 0.347465 2.418385
H -2.773456 3.717407 0.436638
H -2.390997 2.505898 1.665380
H -1.140592 3.110741 0.617866
H -3.022168 3.123661 -1.940309
H -1.430738 2.453464 -2.152942
H -2.815839 1.468494 -2.521131
H -3.855813 -0.461931 3.263609
H -4.166339 0.577092 1.875546
H -4.264417 -1.174794 1.712578
H -1.785866 -1.926845 3.369604
H -2.103503 -2.614405 1.780445
H -0.555682 -1.834969 2.106294
H -4.303490 -2.088735 -1.968928
H -4.497136 -1.277209 -0.418122
H -4.241427 -0.335181 -1.882123
H -2.050804 -2.246543 -3.101385
H -1.957751 -0.491721 -3.128756
H -0.642564 -1.419722 -2.419648
H 1.945545 0.632567 3.700991
H 1.812792 1.855898 2.445028
H 0.533795 0.659557 2.633209
H 4.204319 -0.058038 2.826647
H 4.431718 -0.559105 1.153524
H 4.121673 1.130743 1.537563
H 2.440543 3.549918 -1.124824
H 1.387299 2.914879 0.152684
H 3.100508 2.793040 0.317439
H 4.021455 2.046512 -2.361608
H 4.516366 1.067786 -0.983361
H 3.792836 0.300160 -2.396762
H 3.690703 -3.127204 -1.442513

H 4.248370 -1.481178 -1.155591
H 3.780811 -2.507229 0.198038
H 1.338887 -3.818589 -1.027985
H 1.356466 -3.146956 0.598214
H 0.105811 -2.651796 -0.539066
Li -0.114995 1.917617 -0.785043

64
(*i*Pr$_3$Si)$_2$NLi_tzvp-disp
Si 1.56148 -0.10664 -0.10757
Si -1.68213 0.00744 0.02642
N -0.05767 0.27647 -0.26228
C 2.42497 1.28076 -1.16242
H 1.89488 1.23681 -2.13898
C 3.88888 1.07236 -1.53960
C 2.23773 2.67941 -0.55995
C 2.04936 -1.75935 -0.92520
C 1.00546 -2.84320 -0.66621
C 3.43336 -2.29617 -0.56314
H 2.03864 -1.54183 -2.00383
C 2.17955 -0.07425 1.69549
C 1.36075 0.88448 2.55695
C 3.67226 0.18803 1.88821
H 1.97657 -1.09315 2.05643
C -2.57431 1.70837 0.00774
C -2.36425 2.48777 1.30329
H -3.64577 1.51335 -0.10895
C -2.14253 2.57674 -1.17690
C -2.45955 -1.05297 -1.37062
C -1.68922 -0.93839 -2.68508
C -3.94624 -0.79770 -1.61561
H -2.35304 -2.09015 -1.02303
C -2.09232 -0.78001 1.70622
C -1.29320 -2.05165 1.96624
C -3.58362 -1.02633 1.92347
H -1.76725 -0.03545 2.44395
H -2.80738 3.48916 1.25250
H -2.80748 1.97771 2.15960
H -1.29656 2.60817 1.51372
H -2.82680 3.41175 -1.35514
H -1.18109 3.07246 -0.96201
H -2.07953 2.02266 -2.12149
H -3.78415 -1.35717 2.94831
H -4.18523 -0.13161 1.74392
H -3.95292 -1.81047 1.25725
H -1.49703 -2.45071 2.96598
H -1.54369 -2.83615 1.24666
H -0.22043 -1.87126 1.89279
H -4.35830 -1.50970 -2.33922
H -4.54138 -0.87387 -0.70464
H -4.10901 0.20349 -2.02584
H -2.05229 -1.65688 -3.42804
H -1.81467 0.05584 -3.13119
H -0.62044 -1.10345 -2.54331
H 1.62489 0.78844 3.61573
H 1.54225 1.92460 2.27136
H 0.29187 0.70491 2.45096
H 3.95552 0.07628 2.94041
H 4.29925 -0.48808 1.30636
H 3.93270 1.20987 1.59944
H 2.49585 3.47643 -1.26578
H 1.22772 2.87421 -0.17231
H 2.88749 2.79733 0.30895
H 4.25970 1.89202 -2.16500
H 4.52170 1.02768 -0.65211
H 4.02929 0.14503 -2.09538
H 3.65664 -3.21031 -1.12412
H 4.23591 -1.58537 -0.76364
H 3.48184 -2.55402 0.49826
H 1.20487 -3.73478 -1.27044
H 1.01924 -3.15501 0.38119
H -0.00424 -2.50026 -0.88961
Li 0.10734 1.66251 -1.43165

82
(*t*Bu$_3$Si)$_2$NLi_tzvp
Si 1.674129 -0.060865 -0.007279
Si -1.698461 -0.029823 0.019198
N -0.006647 0.142138 -0.011503
C 2.461170 1.822508 0.089250
C 2.335032 2.622606 -1.225800
C 3.968707 1.773032 0.387213
C 1.874272 2.705249 1.221033
C 2.323191 -0.868811 -1.687972
C 2.157123 -2.397866 -1.655201
C 3.791279 -0.608010 -2.055123
C 1.461670 -0.353860 -2.849396
C 2.402925 -1.066638 1.521834
C 2.362008 -0.269446 2.834988
C 3.852007 -1.547687 1.349123
C 1.532803 -2.313300 1.737539
C -2.490107 1.349470 -1.207465
C -1.734568 2.691481 -1.166182
C -3.960577 1.675574 -0.905540
C -2.418316 0.913064 -2.678434
C -2.398053 -1.782122 -0.545165
C -1.694705 -2.276865 -1.813964
C -3.907625 -1.804223 -0.829486
C -2.130962 -2.839770 0.535659
C -2.354669 0.374239 1.843384
C -1.534617 -0.406742 2.876072
C -3.831355 0.060352 2.120277
C -2.125958 1.857199 2.171490
H -2.304466 3.463559 -1.695332
H -1.574691 3.092436 -0.157183
H -0.789551 2.638163 -1.725295
H -2.741557 1.739487 -3.326067
H -1.405320 0.638706 -2.979623
H -3.074359 0.070878 -2.893488
H -4.085090 0.376164 3.140910
H -4.520016 0.569812 1.446597
H -4.038912 -1.009369 2.063928
H -1.823226 -0.097214 3.889180
H -1.687104 -1.483786 2.813026
H -0.470755 -0.213320 2.758890
H -4.212851 -2.833087 -1.060177
H -4.509447 -1.474588 0.017605
H -4.182040 -1.196241 -1.692608
H -2.038707 -3.291959 -2.050783
H -1.897875 -1.657412 -2.686670
H -0.616412 -2.319665 -1.683459
H 2.585637 -0.941188 3.673203
H 3.107978 0.526373 2.866608
H 1.386594 0.174617 3.035282
H 4.159176 -2.088546 2.253450
H 3.970675 -2.238286 0.514909

H 4.561699 -0.731715 1.209705
H 2.473873 3.619255 1.305441
H 0.846804 3.083673 1.095738
H 1.887147 2.207248 2.187726
H 4.387634 2.786435 0.333069
H 4.175455 1.396820 1.387742
H 4.516650 1.156878 -0.322488
H 4.039349 -1.171742 -2.963666
H 3.995887 0.440648 -2.277132
H 4.487907 -0.930560 -1.279856
H 2.306100 -2.801322 -2.664681
H 2.889464 -2.882091 -1.008797
H 1.168682 -2.714206 -1.325138
Li 0.188866 1.972954 -0.209891
H -4.352839 2.350027 -1.678491
H -4.592839 0.788616 -0.902302
H -4.092054 2.179589 0.052447
H -2.420829 -3.827679 0.155370
H -1.076937 -2.895221 0.809789
H -2.711163 -2.666406 1.442823
H -2.376250 2.047156 3.223112
H -1.075130 2.141313 2.052042
H -2.740720 2.532499 1.574585
H 1.769826 -0.841103 -3.783241
H 0.407387 -0.571987 -2.689316
H 1.559734 0.722669 -3.003978
H 2.714792 3.641316 -1.078700
H 2.915747 2.170184 -2.027830
H 1.322309 2.735789 -1.638612
H 1.909698 -2.877579 2.600115
H 0.498134 -2.047336 1.945207
H 1.543329 -2.987516 0.880924

82
(*t*Bu$_3$Si)$_2$NLi_tzvp-disp
Si 1.661742 -0.060895 -0.007655
Si -1.685151 -0.031846 0.022723
N -0.006964 0.128945 -0.008755
C 2.427749 1.800653 0.038895
C 2.291954 2.560835 -1.294012
C 3.932046 1.754660 0.331975
C 1.835529 2.702112 1.149550
C 2.277940 -0.895730 -1.657088
C 2.099408 -2.418208 -1.577603
C 3.736585 -0.648963 -2.050774
C 1.389715 -0.396996 -2.801395
C 2.385213 -1.017192 1.524239
C 2.349566 -0.185211 2.811457
C 3.826657 -1.507222 1.350314
C 1.502086 -2.245742 1.765956
C -2.465401 1.309114 -1.212234
C -1.704492 2.644731 -1.205289
C -3.929447 1.637467 -0.903633
C -2.396196 0.837506 -2.668225
C -2.371536 -1.774257 -0.499420
C -1.662554 -2.282096 -1.755476
C -3.878429 -1.806513 -0.772102
C -2.082738 -2.797576 0.603236
C -2.312264 0.409362 1.821992
C -1.462345 -0.340775 2.847469
C -3.778667 0.095998 2.125541
C -2.071800 1.896627 2.100967
H -2.274332 3.408130 -1.745087
H -1.532181 3.064291 -0.206775
H -0.765938 2.568789 -1.770905
H -2.717413 1.647781 -3.335851
H -1.382659 0.554692 -2.957054
H -3.052373 -0.009261 -2.859014
H -4.022167 0.429884 3.142272
H -4.475505 0.587254 1.447787
H -3.977091 -0.975783 2.089308
H -1.713593 -0.002010 3.860410
H -1.614860 -1.418290 2.817512
H -0.406290 -0.149143 2.678861
H -4.184994 -2.839675 -0.977548
H -4.470726 -1.455617 0.072457
H -4.156355 -1.215548 -1.645090
H -1.976106 -3.312518 -1.963559
H -1.885495 -1.690482 -2.641221
H -0.584760 -2.285284 -1.624954
H 2.568759 -0.833564 3.668188
H 3.097951 0.607782 2.817144
H 1.376337 0.268747 2.995576
H 4.140612 -2.031548 2.261276
H 3.931183 -2.211893 0.526996
H 4.534826 -0.695548 1.185959
H 2.456776 3.599139 1.246810
H 0.825240 3.109937 0.987278
H 1.804446 2.206913 2.116122
H 4.356383 2.762219 0.239523
H 4.137458 1.411475 1.343636
H 4.469368 1.107625 -0.356952
H 3.971499 -1.230938 -2.950540
H 3.938603 0.394987 -2.293059
H 4.439059 -0.957090 -1.275583
H 2.200043 -2.850915 -2.580153
H 2.853849 -2.888549 -0.947639
H 1.124394 -2.711987 -1.193738
Li 0.181940 1.941296 -0.243913
H -4.333221 2.291517 -1.687199
H -4.554521 0.746990 -0.870092
H -4.048359 2.161234 0.044468
H -2.362842 -3.798483 0.252595
H -1.025301 -2.827326 0.864839
H -2.655154 -2.603811 1.510357
H -2.303122 2.120360 3.149546
H -1.019987 2.163291 1.955881
H -2.688783 2.556331 1.490843
H 1.658446 -0.910182 -3.732652
H 0.340434 -0.593381 -2.595334
H 1.497746 0.673847 -2.980232
H 2.650161 3.590092 -1.174137
H 2.884390 2.094391 -2.078089
H 1.279328 2.635515 -1.712856
H 1.862076 -2.789618 2.647785
H 0.470318 -1.958136 1.950895
H 1.513629 -2.940267 0.927020

28
(Me$_3$Si)$_2$NCs_tzvp
Si -1.24128 -1.54751 0.00007
Si -1.24195 1.54717 0.00012
N -0.58847 -0.00003 0.00051
C 0.16560 -2.84731 -0.00999
H 0.79936 -2.77868 -0.90283
H -0.25600 -3.85668 -0.01383

H 0.80436 -2.78764 0.87995
C -2.29708 -1.97668 -1.51210
H -3.19413 -1.35253 -1.55304
H -2.62281 -3.02165 -1.49843
H -1.74265 -1.80859 -2.44002
C -2.28102 -1.98351 1.52141
H -1.71627 -1.82058 2.44403
H -2.60778 -3.02814 1.50613
H -3.17692 -1.35872 1.57540
C 0.16426 2.84758 0.00685
H 0.79823 2.78160 0.89975
H -0.25772 3.85679 0.00798
H 0.80300 2.78587 -0.88298
C -2.28406 1.98115 -1.52020
H -1.72085 1.81670 -2.44350
H -2.61045 3.02590 -1.50588
H -3.18026 1.35660 -1.57184
C -2.29580 1.97735 1.51338
H -3.19265 1.35303 1.55606
H -2.62178 3.02224 1.49936
H -1.74002 1.81009 2.44065
Cs 2.16888 0.00033 0.00002

28
$(Me_3Si)_2$NCs_tzvp-disp
Si -1.239129 -1.524423 -0.001619
Si -1.239522 1.524294 0.001663
N -0.542099 0.000023 0.000201
C 0.117155 -2.849631 -0.211847
H 0.676832 -2.723865 -1.146048
H -0.327079 -3.848050 -0.246677
H 0.826275 -2.859844 0.624864
C -2.465803 -1.834176 -1.401725
H -3.339932 -1.185972 -1.294544
H -2.820133 -2.869185 -1.416717
H -2.012512 -1.613614 -2.371907
C -2.121019 -1.980139 1.604222
H -1.445413 -1.877403 2.458322
H -2.497728 -3.007358 1.590601
H -2.969742 -1.314275 1.779900
C 0.116317 2.849591 0.214102
H 0.675666 2.722444 1.148310
H -0.327863 3.847980 0.250174
H 0.825786 2.860904 -0.622301
C -2.119299 1.980198 -1.605289
H -1.442640 1.877243 -2.458528
H -2.495768 3.007515 -1.592212
H -2.967983 1.314550 -1.781998
C -2.468044 1.833455 1.400263
H -3.341907 1.185072 1.292090
H -2.822591 2.868396 1.414919
H -2.015770 1.612967 2.370941
Cs 2.181136 0.000152 0.000008

64
$(\textit{i}Pr_3Si)_2$NCs_tzvp
Si -1.501966 0.651205 -0.069914
Si 1.695671 0.661962 0.013986
N 0.098115 0.142021 -0.002082
C -2.575760 -0.418419 1.160499
H -2.732296 -1.374058 0.626899
C -3.981589 0.104955 1.472335
C -1.859674 -0.731329 2.476907
C -2.192489 0.193200 -1.824786
C -1.545537 1.038132 -2.923947
C -3.703275 0.097687 -2.042978
H -1.796261 -0.829091 -1.959880
C -1.768734 2.519676 0.292417
C -1.497595 2.875901 1.757486
C -3.084311 3.151895 -0.167907
H -0.971559 2.987174 -0.298250
C 2.622330 0.083316 1.604957
C 2.094765 0.765505 2.868329
H 3.669149 0.384329 1.470501
C 2.615623 -1.430898 1.815545
C 2.706247 -0.011382 -1.507017
C 2.199742 -1.308225 -2.134550
C 4.219511 -0.103685 -1.293442
H 2.536377 0.767146 -2.262771
C 1.945121 2.577477 -0.046439
C 1.579493 3.195035 -1.399079
C 3.317123 3.105620 0.384002
H 1.215241 2.946754 0.685583
Cs -0.219381 -2.756048 -0.073535
H 2.647678 0.445271 3.760256
H 2.168168 1.853549 2.814151
H 1.041464 0.520420 3.030719
H 3.266999 -1.729551 2.645658
H 1.609356 -1.772677 2.085665
H 2.950579 -1.984084 0.931652
H 3.311354 4.200922 0.438990
H 3.630945 2.734316 1.361303
H 4.095402 2.834697 -0.334907
H 1.518896 4.287717 -1.332505
H 2.337785 2.967898 -2.154930
H 0.622718 2.836219 -1.782484
H 4.741310 -0.331837 -2.230992
H 4.643612 0.823281 -0.904865
H 4.477947 -0.895993 -0.584053
H 2.699595 -1.517255 -3.088007
H 2.408581 -2.174649 -1.492476
H 1.125696 -1.256425 -2.334932
H -1.427697 3.961660 1.892429
H -2.303526 2.528206 2.410099
H -0.567480 2.436623 2.123944
H -3.084856 4.229770 0.034555
H -3.251778 3.027113 -1.239389
H -3.949050 2.731357 0.350830
H -2.367595 -1.522756 3.042754
H -0.813691 -1.013865 2.329788
H -1.836083 0.151672 3.120311
H -4.549981 -0.619660 2.068203
H -3.933280 1.025852 2.057588
H -4.563680 0.319578 0.575927
H -3.930902 -0.220944 -3.067838
H -4.175680 -0.621598 -1.369982
H -4.200161 1.058975 -1.893107
H -1.798004 0.660169 -3.922079
H -1.886532 2.076627 -2.882009
H -0.457456 1.044553 -2.834339

64
$(\textit{i}Pr_3Si)_2$NCs_tzvp-disp
Si -1.458031 0.656414 -0.078800
Si 1.696053 0.626782 0.015435
N 0.113124 0.089237 -0.001186

C -2.557263 -0.348584 1.156790
H -2.759219 -1.304990 0.641824
C -3.929472 0.247702 1.467506
C -1.827675 -0.658011 2.463327
C -2.147040 0.213979 -1.817812
C -1.424171 1.017400 -2.895597
C -3.656095 0.218758 -2.039305
H -1.815190 -0.831563 -1.943192
C -1.642696 2.520103 0.268414
C -1.346573 2.842696 1.733105
C -2.932747 3.197189 -0.187019
H -0.830285 2.954156 -0.323293
C 2.583629 0.060521 1.613453
C 2.004439 0.763798 2.838135
H 3.640041 0.337480 1.518420
C 2.517005 -1.449464 1.819723
C 2.708144 -0.057146 -1.478095
C 2.154521 -1.334408 -2.097569
C 4.204439 -0.210067 -1.212720
H 2.588333 0.724257 -2.239188
C 1.932958 2.525944 -0.059923
C 1.565315 3.112570 -1.422102
C 3.316415 3.028757 0.350309
H 1.213710 2.911074 0.672261
Cs -0.297841 -2.740446 -0.074178
H 2.493398 0.438772 3.764238
H 2.106622 1.848894 2.776178
H 0.937817 0.543493 2.932511
H 3.129305 -1.776921 2.667560
H 1.488338 -1.744980 2.054115
H 2.853620 -2.009666 0.941294
H 3.341520 4.124111 0.383202
H 3.623545 2.664787 1.332216
H 4.079600 2.718482 -0.368296
H 1.488231 4.204768 -1.379212
H 2.331093 2.877429 -2.167158
H 0.617897 2.728701 -1.802336
H 4.751094 -0.466643 -2.127788
H 4.648027 0.702007 -0.811721
H 4.399520 -1.005610 -0.487618
H 2.648876 -1.575919 -3.045377
H 2.324846 -2.196942 -1.440047
H 1.083586 -1.236900 -2.295820
H -1.207831 3.919298 1.882261
H -2.169809 2.534322 2.382751
H -0.445206 2.338371 2.085949
H -2.904689 4.270826 0.032502
H -3.090893 3.091370 -1.261626
H -3.812136 2.790249 0.316160
H -2.345963 -1.421039 3.056589
H -0.793929 -0.971494 2.296276
H -1.761827 0.239004 3.082802
H -4.528153 -0.432208 2.084783
H -3.827309 1.179342 2.027062
H -4.503967 0.470034 0.568501
H -3.905887 -0.080443 -3.064225
H -4.169458 -0.470282 -1.365198
H -4.086378 1.209636 -1.881684
H -1.685637 0.673016 -3.902806
H -1.685606 2.077901 -2.840413
H -0.341320 0.938518 -2.780979

(tBu$_3$Si)$_2$NCs_tzvp

Si -0.556514 1.786219 0.000513
Si -1.273728 -1.459599 -0.000546
N -0.775456 0.132366 -0.010173
C 0.498913 2.376023 -1.593403
C 1.953433 1.906268 -1.481786
C 0.547039 3.884420 -1.864612
C -0.059989 1.688983 -2.845607
C 0.486280 2.326139 1.623204
C -0.413805 2.306182 2.867696
C 1.161857 3.702979 1.587629
C 1.594512 1.293453 1.869840
C -2.216591 2.871451 -0.001882
C -2.860694 2.928424 -1.396696
C -2.045949 4.331548 0.444995
C -3.248773 2.228354 0.933253
C 0.272788 -2.642092 -0.528422
C 1.029006 -1.969216 -1.673410
C -0.055832 -4.072363 -0.968499
C 1.248500 -2.759491 0.644442
C -1.849562 -2.089287 1.796190
C -0.897619 -1.572730 2.882667
C -1.958565 -3.609714 1.976933
C -3.227325 -1.498617 2.129312
C -2.727172 -1.855116 -1.290574
C -3.830612 -0.798166 -1.182504
C -3.407977 -3.221394 -1.134483
C -2.201311 -1.760702 -2.730259
H 1.984167 -2.491749 -1.868124
H 0.475762 -1.984305 -2.611779
H 1.208724 -0.916087 -1.444967
H 2.196180 -3.219064 0.305251
H 1.451575 -1.786946 1.104891
H 0.873440 -3.403801 1.438416
H -4.137840 -3.358662 -1.944196
H -2.715051 -4.061980 -1.182219
H -3.962140 -3.298335 -0.197595
H -4.585440 -0.970211 -1.961917
H -4.347806 -0.817341 -0.223348
H -3.430502 0.202448 -1.323821
H -2.340350 -3.831484 2.983150
H -2.640145 -4.077504 1.266502
H -0.994756 -4.115865 1.892348
H -1.336485 -1.745115 3.874721
H 0.070456 -2.075259 2.876721
H -0.719060 -0.504774 2.777314
H -3.867502 3.359224 -1.315839
H -2.302050 3.560922 -2.088061
H -2.966400 1.947968 -1.859721
H -3.006686 4.854519 0.343812
H -1.751183 4.422858 1.490839
H -1.319644 4.880487 -0.156252
H 0.568641 1.921885 -3.717324
H -0.082425 0.606295 -2.720843
H -1.072399 2.013114 -3.081870
H 1.185930 4.084716 -2.736881
H -0.435092 4.294843 -2.096507
H 0.954298 4.454970 -1.028677
H 1.666358 3.896155 2.545470
H 1.923173 3.778796 0.808086
H 0.452148 4.515237 1.432089
H 0.198368 2.406113 3.774969
H -1.123149 3.133333 2.872425

H -0.985926 1.383529 2.958110
H 0.873282 -4.625182 -1.176497
H -0.598290 -4.632691 -0.206412
H -0.647585 -4.097143 -1.883303
H -3.489539 -1.738388 3.168816
H -3.240537 -0.412341 2.031382
H -4.019367 -1.906724 1.500255
H -3.042455 -1.822088 -3.433877
H -1.692425 -0.812894 -2.914808
H -1.518963 -2.572892 -2.987250
H 2.014130 1.406599 2.879393
H 1.210057 0.276620 1.758356
H 2.415882 1.456288 1.164208
H 2.487829 2.088755 -2.425707
H 2.502887 2.417807 -0.687614
H 1.963636 0.829397 -1.299024
H -4.187038 2.798616 0.897378
H -3.471220 1.205560 0.636753
H -2.921057 2.209816 1.972967
Cs 3.901488 -0.701806 -0.008958

82
(*t*Bu$_3$Si)$_2$NCs_tzvp-disp
Si -0.52076 1.77345 -0.00271
Si -1.24738 -1.44538 -0.00270
N -0.72064 0.12708 -0.02320
C 0.51419 2.35490 -1.57884
C 1.95374 1.86188 -1.45164
C 0.57440 3.85799 -1.84747
C -0.04928 1.65555 -2.81822
C 0.51201 2.27818 1.60489
C -0.40480 2.22845 2.83217
C 1.18662 3.65085 1.59325
C 1.61140 1.23352 1.82505
C -2.16137 2.84242 0.00586
C -2.79551 2.89817 -1.39014
C -1.98002 4.29659 0.45432
C -3.18893 2.19754 0.93921
C 0.24411 -2.63088 -0.57236
C 0.98892 -1.93974 -1.70959
C -0.12159 -4.04195 -1.02882
C 1.22488 -2.78456 0.58684
C -1.76591 -2.04867 1.79237
C -0.75818 -1.55341 2.83470
C -1.92078 -3.56036 1.97485
C -3.10601 -1.40339 2.15808
C -2.72313 -1.79707 -1.24037
C -3.79353 -0.71626 -1.10022
C -3.42292 -3.14739 -1.07408
C -2.21675 -1.69079 -2.68258
H 1.94174 -2.45626 -1.92457
H 0.42395 -1.93396 -2.63984
H 1.16299 -0.89153 -1.45886
H 2.16926 -3.23495 0.23027
H 1.42590 -1.82727 1.07661
H 0.84936 -3.44995 1.36149
H -4.16710 -3.27457 -1.87147
H -2.74372 -3.99774 -1.12637
H -3.96135 -3.20750 -0.12720
H -4.56310 -0.85301 -1.87120
H -4.29481 -0.73601 -0.13371
H -3.36199 0.27110 -1.23046
H -2.26359 -3.77471 2.99592
H -2.64930 -3.99560 1.29195
H -0.98093 -4.09820 1.84010
H -1.18932 -1.63583 3.84059
H 0.16507 -2.13229 2.84008
H -0.49898 -0.51155 2.66379
H -3.80971 3.31019 -1.31463
H -2.23883 3.54404 -2.06939
H -2.87665 1.91987 -1.86079
H -2.93317 4.83056 0.34602
H -1.69208 4.37880 1.50221
H -1.24040 4.83421 -0.13949
H 0.58851 1.85628 -3.69038
H -0.09193 0.57808 -2.66248
H -1.05418 1.99184 -3.06454
H 1.22011 4.05692 -2.71418
H -0.40434 4.27347 -2.08172
H 0.97846 4.41950 -1.00444
H 1.69366 3.82979 2.55172
H 1.94341 3.73430 0.81061
H 0.47535 4.46277 1.44869
H 0.19300 2.28171 3.75183
H -1.09795 3.06812 2.85225
H -0.99515 1.31429 2.87307
H 0.79055 -4.61180 -1.26041
H -0.66461 -4.59696 -0.26374
H -0.73103 -4.03680 -1.93130
H -3.35029 -1.62494 3.20521
H -3.07348 -0.31936 2.04896
H -3.92703 -1.78323 1.54994
H -3.06744 -1.72359 -3.37546
H -1.69290 -0.74867 -2.85134
H -1.55151 -2.50905 -2.95976
H 1.96133 1.24119 2.86557
H 1.23817 0.23504 1.58067
H 2.47362 1.47703 1.19728
H 2.50128 2.01725 -2.39207
H 2.50367 2.36796 -0.65555
H 1.92209 0.78993 -1.25471
H -4.13588 2.75065 0.88766
H -3.38876 1.16836 0.65566
H -2.86692 2.19910 1.97964
Cs 3.78775 -0.70853 -0.01315

82
(*t*Bu$_3$Si)$_2$NNa_tzvp-disp
Si 1.663470 -0.111215 -0.026464
Si -1.669872 -0.071947 0.009160
N -0.000926 0.099533 -0.001988
C 2.549131 1.472212 0.844175
C 2.700493 2.734848 -0.028606
C 3.993864 1.159907 1.261657
C 1.835487 1.877162 2.142192
C 2.331667 -0.154435 -1.862288
C 1.961492 -1.487526 -2.518638
C 3.840615 0.033873 -2.031812
C 1.621731 0.926453 -2.679753
C 2.283375 -1.700702 0.929268
C 2.040890 -1.531165 2.433339
C 3.747493 -2.090811 0.699690
C 1.462571 -2.923278 0.516099
C -2.470112 0.927739 -1.506554
C -1.826606 2.290569 -1.801736
C -3.968627 1.198940 -1.343818

C -2.275632 0.110882 -2.790021
C -2.315197 -1.908992 -0.107337
C -1.512471 -2.658597 -1.178085
C -3.796907 -2.099337 -0.449017
C -2.086151 -2.608023 1.241576
C -2.382856 0.719379 1.674983
C -1.534407 0.247794 2.860102
C -3.842745 0.361126 1.969305
C -2.312767 2.253751 1.693477
H -2.315001 2.744014 -2.671499
H -1.958503 3.020034 -0.993598
H -0.772260 2.199682 -2.078722
H -2.547350 0.715305 -3.664762
H -1.242836 -0.216024 -2.919024
H -2.908369 -0.773970 -2.806067
H -4.151467 0.822906 2.916020
H -4.529160 0.710782 1.198858
H -3.984970 -0.711911 2.078843
H -1.806533 0.805517 3.765541
H -1.679173 -0.808807 3.075678
H -0.473443 0.402113 2.667605
H -4.038118 -3.169036 -0.409099
H -4.465716 -1.592797 0.246392
H -4.039937 -1.760739 -1.456175
H -1.659090 -3.739375 -1.064880
H -1.829865 -2.393262 -2.184916
H -0.448730 -2.458635 -1.105362
H 2.213406 -2.486627 2.943730
H 2.707358 -0.802460 2.894821
H 1.011623 -1.230872 2.639363
H 3.991039 -2.952772 1.333529
H 3.918593 -2.401537 -0.332095
H 4.461938 -1.305619 0.934517
H 2.360498 2.718828 2.610657
H 0.790429 2.182068 2.039105
H 1.813915 1.060835 2.861050
H 4.457872 2.060750 1.683693
H 4.041059 0.389692 2.027675
H 4.608530 0.840252 0.421520
H 4.106774 -0.073928 -3.091038
H 4.180773 1.022014 -1.722013
H 4.419515 -0.703308 -1.476836
H 2.186566 -1.445583 -3.591900
H 2.528392 -2.321476 -2.104763
H 0.898780 -1.709462 -2.414747
Na -0.030279 2.344693 0.033775
H -4.373744 1.616043 -2.274915
H -4.535592 0.298113 -1.118327
H -4.172555 1.924479 -0.554506
H -2.167885 -3.693657 1.113084
H -1.108324 -2.401258 1.673112
H -2.834081 -2.316188 1.979008
H -2.827530 2.633496 2.583917
H -1.288736 2.630368 1.807029
H -2.785422 2.728617 0.833125
H 1.933360 0.870882 -3.730136
H 0.543720 0.779656 -2.646596
H 1.846295 1.939138 -2.339355
H 3.107604 3.549025 0.583121
H 3.395506 2.571379 -0.849804
H 1.806505 3.149250 -0.510097
H 1.782058 -3.787951 1.111102
H 0.405343 -2.775159 0.696586
H 1.590147 -3.187534 -0.531997

82
(*t*Bu$_3$Si)$_2$NK_tzvp-disp
Si 1.646845 -0.188797 -0.045424
Si -1.666727 -0.127072 0.027393
N -0.004405 0.121256 0.004199
C 2.633019 1.205612 1.033934
C 2.896314 2.545899 0.286178
C 4.026855 0.788372 1.525422
C 1.828239 1.502607 2.306723
C 2.306338 -0.018717 -1.886333
C 2.041917 -1.308910 -2.668824
C 3.800717 0.285977 -2.001576
C 1.521287 1.065897 -2.629419
C 2.238604 -1.913004 0.684827
C 1.967723 -1.934708 2.194956
C 3.714239 -2.249116 0.431265
C 1.461993 -3.103051 0.117039
C -2.601340 0.843842 -1.442127
C -2.111591 2.255909 -1.779819
C -4.105423 0.992245 -1.190610
C -2.412610 0.055393 -2.744554
C -2.222981 -1.996240 -0.165423
C -1.434191 -2.640446 -1.316573
C -3.703985 -2.284689 -0.437915
C -1.887846 -2.726582 1.144202
C -2.392646 0.525259 1.745710
C -1.518381 0.013405 2.896871
C -3.834733 0.109797 2.052182
C -2.348959 2.056913 1.838192
H -2.706453 2.652061 -2.611124
H -2.250176 2.969235 -0.960863
H -1.077230 2.258216 -2.132261
H -2.776902 0.646614 -3.594490
H -1.365103 -0.186912 -2.932281
H -2.973598 -0.875072 -2.742732
H -4.127931 0.506909 3.032822
H -4.554017 0.481926 1.324354
H -3.943041 -0.971985 2.105048
H -1.742794 0.569079 3.816723
H -1.693712 -1.039208 3.108081
H -0.460646 0.134195 2.671187
H -3.858996 -3.371282 -0.443992
H -4.368939 -1.870825 0.319665
H -4.032216 -1.916609 -1.409738
H -1.352247 -3.722634 -1.165407
H -1.927855 -2.488603 -2.274501
H -0.428111 -2.240928 -1.399824
H 2.126110 -2.947800 2.584381
H 2.622938 -1.272962 2.759739
H 0.936657 -1.654241 2.418868
H 3.959668 -3.183948 0.950942
H 3.905965 -2.421147 -0.629090
H 4.415921 -1.496221 0.776182
H 2.280732 2.332086 2.865048
H 0.778505 1.739381 2.128527
H 1.804920 0.639548 2.970363
H 4.468287 1.614024 2.098802
H 4.004050 -0.076426 2.183298
H 4.702762 0.572075 0.698106
H 4.096374 0.315053 -3.058273
H 4.068125 1.249626 -1.569113

H 4.411922 -0.475907 -1.517172
H 2.305189 -1.159658 -3.723801
H 2.639677 -2.143021 -2.304368
H 0.991231 -1.600171 -2.634253
K 0.075040 2.810145 0.004204
H -4.597999 1.358904 -2.100809
H -4.584905 0.055847 -0.916394
H -4.314899 1.716405 -0.400807
H -1.888554 -3.810384 0.978417
H -0.916724 -2.455296 1.553507
H -2.633213 -2.520808 1.912906
H -2.905847 2.392823 2.720989
H -1.328426 2.415290 2.001578
H -2.789145 2.563843 0.976754
H 1.770337 1.051266 -3.697513
H 0.450264 0.892896 -2.529273
H 1.746230 2.080339 -2.286577
H 2.609204 3.409862 0.901242
H 3.960446 2.675683 0.088455
H 2.432326 2.641876 -0.694547
H 1.838813 -4.021934 0.583978
H 0.405038 -3.042392 0.334938
H 1.576062 -3.218045 -0.958497

82
(*t*$Bu_3Si)_2$NRb_tzvp-disp
Si -1.632941 0.428179 -0.049334
Si 1.668849 0.324626 0.026908
N 0.007766 0.053316 0.003755
C -2.672052 -0.944359 1.013000
C -3.019851 -2.229867 0.214556
C -4.036963 -0.516745 1.572980
C -1.824720 -1.312810 2.239493
C -2.292577 0.317875 -1.896354
C -1.971285 1.610688 -2.652392
C -3.798657 0.084502 -2.028026
C -1.551207 -0.787028 -2.654058
C -2.185658 2.152513 0.718161
C -1.901870 2.126262 2.226234
C -3.661443 2.503027 0.483514
C -1.416001 3.362455 0.184507
C 2.614207 -0.593513 -1.468879
C 2.128083 -1.997446 -1.831018
C 4.120420 -0.740801 -1.232082
C 2.408889 0.220837 -2.752767
C 2.222566 2.204056 -0.127456
C 1.431200 2.874578 -1.260660
C 3.701569 2.501098 -0.405364
C 1.899901 2.910720 1.197921
C 2.419360 -0.341870 1.732285
C 1.555305 0.146533 2.901705
C 3.861665 0.079754 2.029443
C 2.387537 -1.873675 1.809111
H 2.693143 -2.363583 -2.696721
H 2.308936 -2.725823 -1.034944
H 1.079088 -1.995144 -2.134458
H 2.770977 -0.350423 -3.617318
H 1.357414 0.456463 -2.926796
H 2.961953 1.155725 -2.737901
H 4.168216 -0.332022 3.000057
H 4.575451 -0.275076 1.287702
H 3.963664 1.160837 2.100064
H 1.799610 -0.418235 3.811153
H 1.721231 1.198059 3.124637
H 0.495465 0.016142 2.691151
H 3.852535 3.588303 -0.389300
H 4.374946 2.074048 0.336988
H 4.022699 2.155959 -1.387727
H 1.360968 3.954989 -1.091294
H 1.914928 2.734299 -2.225116
H 0.421227 2.486879 -1.342073
H -2.048651 3.127978 2.648485
H -2.555443 1.451796 2.776550
H -0.871824 1.829658 2.432248
H -3.908004 3.405790 1.056800
H -3.847572 2.738789 -0.565775
H -4.365255 1.732214 0.779186
H -2.208361 -2.218705 2.728237
H -0.766039 -1.439694 2.012843
H -1.847899 -0.516123 2.982285
H -4.470651 -1.354531 2.135804
H -3.978570 0.326423 2.256357
H -4.741114 -0.266713 0.778777
H -4.087493 0.101775 -3.087000
H -4.112338 -0.878840 -1.629078
H -4.378831 0.857338 -1.523753
H -2.240130 1.493823 -3.710151
H -2.534296 2.461415 -2.271051
H -0.909733 1.856640 -2.612828
Rb -0.107718 -2.945124 -0.001262
H 4.606817 -1.090873 -2.152301
H 4.600271 0.191387 -0.945442
H 4.337221 -1.478197 -0.456313
H 1.898638 3.997505 1.051770
H 0.933563 2.632035 1.612668
H 2.653182 2.692544 1.955240
H 2.969027 -2.216621 2.673392
H 1.370860 -2.229483 1.992846
H 2.806385 -2.368470 0.929657
H -1.784109 -0.736420 -3.724916
H -0.474507 -0.670762 -2.533781
H -1.831940 -1.794606 -2.333720
H -2.893258 -3.127935 0.835636
H -4.069089 -2.226878 -0.078626
H -2.484485 -2.366031 -0.722824
H -1.804052 4.265096 0.673808
H -0.359993 3.308329 0.406171
H -1.526776 3.505338 -0.888080

27
$(Me_3Si)_2N^-$_tzvp
Si -1.633508 -0.000400 -0.000515
Si 1.633508 -0.000400 -0.000515
N 0.000000 -0.002696 -0.003208
C -2.420602 -1.241377 -1.222642
H -2.097011 -1.023099 -2.245520
H -3.516679 -1.219726 -1.201194
H -2.096886 -2.260774 -0.988805
C -2.416120 1.680841 -0.463232
H -2.089965 2.456466 0.237251
H -3.512266 1.654480 -0.454525
H -2.092176 1.987393 -1.463095
C -2.415590 -0.437959 1.687710
H -2.091269 -1.432992 2.009214
H -3.511747 -0.429969 1.661466
H -2.089495 0.274216 2.452636

C 2.420602 -1.241523 -1.222493
H 2.096878 -2.260892 -0.988539
H 3.516679 -1.219877 -1.201040
H 2.097019 -1.023363 -2.245399
C 2.416120 1.680786 -0.463434
H 2.092160 1.987226 -1.463326
H 3.512266 1.654420 -0.454743
H 2.089982 2.456491 0.236968
C 2.415590 -0.437756 1.687762
H 2.089483 0.274503 2.452606
H 3.511747 -0.429756 1.661521
H 2.091280 -1.432757 2.009380

27
$(Me_3Si)_2N^-$_tzvp-disp
Si -1.552916 -0.000744 0.108313
Si 1.552920 0.000724 0.108317
N 0.000001 -0.000042 0.645471
C -2.799222 -0.190191 1.532345
H -2.623119 -1.130841 2.063225
H -3.838384 -0.180793 1.185038
H -2.670773 0.623090 2.253270
C -1.994231 -1.400984 -1.110956
H -1.394146 -1.310293 -2.022091
H -3.051830 -1.395004 -1.399259
H -1.765771 -2.373834 -0.664932
C -2.092503 1.589888 -0.796234
H -1.946467 2.458616 -0.146792
H -3.143868 1.565786 -1.105661
H -1.477967 1.742586 -1.688969
C 2.799239 0.190927 1.532237
H 2.622947 1.131691 2.062851
H 3.838373 0.181692 1.184833
H 2.671080 -0.622175 2.253415
C 2.092764 -1.590158 -0.795625
H 1.947133 -2.458653 -0.145790
H 3.144051 -1.565867 -1.105300
H 1.478068 -1.743402 -1.688157
C 1.993948 1.400571 -1.111513
H 1.393774 1.309422 -2.022544
H 3.051515 1.394686 -1.399934
H 1.765351 2.373554 -0.665848

63
(*i*$Pr_3Si)_2N^-$_tzvp
Si 1.573818 -0.024722 -0.144654
Si -1.687596 0.011187 0.008317
N -0.062617 0.139658 -0.200334
C 2.472279 1.413925 -1.091000
H 2.419091 1.080707 -2.139298
C 3.946428 1.658193 -0.762989
C 1.705345 2.732649 -1.009135
C 2.126028 -1.639834 -1.071265
C 1.660764 -2.901199 -0.344090
C 3.577526 -1.788986 -1.526746
H 1.516046 -1.556611 -1.982530
C 2.196473 -0.129326 1.689138
C 1.891129 1.155445 2.461085
C 3.627804 -0.589291 1.966758
H 1.527029 -0.902728 2.091610
C -2.636833 1.662406 -0.325741
C -2.187195 2.763047 0.636533
H -3.702957 1.467675 -0.141542
C -2.497782 2.145248 -1.769747
C -2.487038 -1.350232 -1.122009
C -1.833884 -1.433769 -2.501713
C -4.009490 -1.301831 -1.262698
H -2.237155 -2.285563 -0.599850
C -2.151569 -0.444935 1.840715
C -1.648417 -1.833147 2.234885
C -3.603445 -0.270646 2.287455
H -1.547909 0.282069 2.402418
H -2.711555 3.709391 0.442029
H -2.368054 2.497107 1.681792
H -1.115163 2.950567 0.532294
H -2.973857 3.125502 -1.914997
H -1.445503 2.244965 -2.049176
H -2.955184 1.452160 -2.480342
H -3.707904 -0.413498 3.372525
H -3.998096 0.720628 2.050276
H -4.262266 -1.004329 1.812304
H -1.727009 -2.003388 3.317784
H -2.231698 -2.621567 1.746164
H -0.603758 -1.973640 1.949878
H -4.387067 -2.153922 -1.845606
H -4.521137 -1.316873 -0.297686
H -4.332341 -0.394284 -1.783055
H -2.159323 -2.330468 -3.048041
H -2.092725 -0.569049 -3.120806
H -0.746413 -1.453111 -2.418693
H 2.027107 1.020365 3.543383
H 2.555246 1.971634 2.156727
H 0.864411 1.484542 2.286962
H 3.808870 -0.690026 3.046609
H 3.845171 -1.558945 1.512583
H 4.367766 0.120460 1.586942
H 2.118941 3.483512 -1.697430
H 0.651445 2.584797 -1.244251
H 1.753920 3.157830 -0.001328
H 4.389966 2.395352 -1.447226
H 4.062007 2.060299 0.248331
H 4.553709 0.752564 -0.821852
H 3.716191 -2.710807 -2.109804
H 3.895652 -0.957128 -2.159908
H 4.273192 -1.841992 -0.683707
H 1.788756 -3.798901 -0.965076
H 2.229524 -3.067806 0.577269
H 0.605515 -2.831919 -0.071218

63
(*i*$Pr_3Si)_2N^-$_tzvp-disp
Si 1.54560 -0.02461 -0.20779
Si -1.66388 0.02140 -0.00694
N -0.07268 0.18806 -0.35306
C 2.50740 1.38324 -1.10424
H 2.51941 1.05049 -2.15340
C 3.95324 1.61031 -0.67210
C 1.73121 2.69587 -1.05819
C 2.09313 -1.64836 -1.08162
C 1.51289 -2.86534 -0.36860
C 3.57112 -1.84565 -1.40083
H 1.56269 -1.55322 -2.04001
C 2.06214 -0.11593 1.64362
C 1.74808 1.20289 2.34608
C 3.46057 -0.60793 2.00076
H 1.34878 -0.85594 2.02940

C -2.61284 1.66017 -0.31016
C -2.06061 2.74413 0.61265
H -3.67595 1.51037 -0.07974
C -2.49125 2.10058 -1.76622
C -2.50581 -1.35406 -1.05926
C -1.87295 -1.46597 -2.44323
C -4.02529 -1.25680 -1.16333
H -2.26735 -2.28327 -0.52266
C -1.99524 -0.41493 1.84132
C -1.49613 -1.81359 2.18865
C -3.41896 -0.21914 2.35305
H -1.34843 0.30069 2.36663
H -2.53310 3.71849 0.42696
H -2.20949 2.49947 1.66797
H -0.98466 2.85676 0.45522
H -2.90853 3.10469 -1.92482
H -1.44089 2.12047 -2.06897
H -3.00943 1.41715 -2.44346
H -3.48235 -0.37100 3.43956
H -3.80164 0.78116 2.13719
H -4.10706 -0.93576 1.89452
H -1.49579 -1.99211 3.27241
H -2.13349 -2.58121 1.73691
H -0.48124 -1.97663 1.82203
H -4.45011 -2.10422 -1.71890
H -4.50712 -1.23252 -0.18339
H -4.32530 -0.34580 -1.69012
H -2.22590 -2.35726 -2.98033
H -2.11475 -0.59583 -3.06070
H -0.78572 -1.50574 -2.36566
H 1.80578 1.10734 3.43889
H 2.45542 1.98572 2.05411
H 0.74660 1.55171 2.08531
H 3.59847 -0.66073 3.08994
H 3.65352 -1.60602 1.60127
H 4.23821 0.05388 1.61187
H 2.18083 3.45757 -1.71027
H 0.69448 2.53817 -1.35559
H 1.71330 3.10527 -0.04340
H 4.45215 2.35190 -1.31108
H 3.99724 1.99359 0.35135
H 4.55044 0.69679 -0.70056
H 3.73977 -2.78053 -1.95350
H 3.96373 -1.03060 -2.01336
H 4.18280 -1.89627 -0.49579
H 1.66196 -3.78874 -0.94460
H 1.98228 -3.01642 0.60941
H 0.44080 -2.74033 -0.20289

81
(tBu$_3$Si)$_2$N$^-$_tzvp
Si 1.659824 -0.000071 -0.000116
Si -1.659375 -0.000021 -0.000145
N -0.000010 -0.000045 -0.000177
C 2.406114 -1.171102 -1.432516
C 2.259488 -2.653954 -1.060898
C 3.884481 -0.954562 -1.782166
C 1.596962 -0.960352 -2.718636
C 2.406339 -0.655187 1.730282
C 2.261332 0.408422 2.828566
C 3.884418 -1.067312 1.716428
C 1.596638 -1.873687 2.191816
C 2.406468 1.826056 -0.297830
C 2.261303 2.246105 -1.767891
C 3.884635 2.020615 0.065537
C 1.596670 2.834468 0.527227
C -2.409393 -1.821164 -0.337450
C -1.641970 -2.500143 -1.476041
C -3.898267 -1.884101 -0.701523
C -2.202033 -2.715365 0.892298
C -2.409486 0.618427 1.745579
C -1.641619 -0.027652 2.902848
C -3.898057 0.333525 1.982264
C -2.202820 2.130632 1.905178
C -2.409709 1.202861 -1.408230
C -1.641457 2.527906 -1.427612
C -3.898221 1.550394 -1.278941
C -2.203878 0.585249 -2.797920
H -1.985343 -3.539615 -1.588782
H -1.780935 -2.006951 -2.438203
H -0.574632 -2.520293 -1.267955
H -2.462392 -3.754842 0.642277
H -1.162451 -2.708655 1.225830
H -2.831596 -2.423887 1.734784
H -4.209892 2.160223 -2.140619
H -4.544449 0.671664 -1.256495
H -4.107777 2.140072 -0.384359
H -1.985006 3.145380 -2.271314
H -1.779297 3.114897 -0.519445
H -0.574351 2.356954 -1.549858
H -4.209312 0.775709 2.940966
H -4.544390 0.753108 1.209925
H -4.107956 -0.735885 2.046674
H -1.985190 0.394263 3.859450
H -1.779960 -1.107610 2.956825
H -0.574431 0.163250 2.816186
H 2.480617 3.319263 -1.870321
H 2.955850 1.715675 -2.421716
H 1.254917 2.080421 -2.152933
H 4.193069 3.047208 -0.184364
H 4.075080 1.886061 1.131413
H 4.547663 1.345238 -0.478215
H 1.921614 -1.678013 -3.487189
H 0.533000 -1.110158 -2.543166
H 1.725340 0.040086 -3.133164
H 4.192822 -1.684545 -2.546056
H 4.075447 0.035645 -2.198691
H 4.547161 -1.087934 -0.925128
H 4.193551 -1.363737 2.730356
H 4.074046 -1.923534 1.067224
H 4.547390 -0.259101 1.402348
H 2.480336 -0.038969 3.809456
H 2.956060 1.239632 2.695724
H 1.255062 0.825080 2.877079
H -4.209227 -2.935542 -0.797931
H -4.544113 -1.425471 0.048746
H -4.109349 -1.405039 -1.659538
H -2.464147 2.433763 2.930190
H -1.163214 2.416553 1.733485
H -2.832019 2.714240 1.230979
H -2.465328 1.321699 -3.572581
H -1.164401 0.293386 -2.960219
H -2.833434 -0.290180 -2.966338
H 1.921614 -2.180103 3.197605
H 0.532867 -1.646026 2.234348
H 1.724105 -2.733435 1.533135

H 2.478330 -3.279869 -1.938734
H 2.953515 -2.955216 -0.274271
H 1.252730 -2.903179 -0.724898
H 1.921780 3.858893 0.290644
H 0.532924 2.757656 0.308508
H 1.723851 2.692882 1.601018

81
(*t*Bu$_3$Si)$_2$N$^-$_tzvp-disp
Si 1.64947 0.00005 -0.00018
Si -1.64731 0.00005 0.00007
N 0.00079 0.00013 -0.00010
C 2.37449 0.32263 1.80173
C 2.21373 1.79837 2.18141
C 3.84855 -0.03226 2.00636
C 1.55195 -0.48650 2.80809
C 2.37415 1.39956 -1.18033
C 2.21280 0.99104 -2.64830
C 3.84816 1.75426 -0.97541
C 1.55171 2.67554 -0.98164
C 2.37497 -1.72173 -0.62169
C 2.21375 -2.78852 0.46633
C 3.84916 -1.72094 -1.03059
C 1.55333 -2.18866 -1.82630
C -2.37812 1.39174 1.19098
C -1.59470 1.40912 2.50302
C -3.86140 1.26359 1.54161
C -2.16198 2.77128 0.56350
C -2.37800 0.33534 -1.80082
C -1.59592 1.46414 -2.47143
C -3.86174 0.70106 -1.86558
C -2.15967 -0.89747 -2.68179
C -2.37733 -1.72761 0.60999
C -1.59365 -2.87204 -0.03167
C -3.86055 -1.96811 0.32433
C -2.16013 -1.87413 2.11833
H -1.91972 2.25901 3.12091
H -1.73114 0.50435 3.09358
H -0.53096 1.51783 2.31055
H -2.40837 3.55431 1.29537
H -1.12188 2.91298 0.26478
H -2.79409 2.93707 -0.30953
H -4.17300 -2.92423 0.76987
H -4.50483 -1.19139 0.73730
H -4.06434 -2.03505 -0.74560
H -1.91664 -3.83224 0.39658
H -1.73220 -2.93200 -1.11022
H -0.52968 -2.75814 0.15653
H -4.17462 0.79393 -2.91619
H -4.50498 -0.04624 -1.39997
H -4.06660 1.66039 -1.38760
H -1.92111 1.57442 -3.51632
H -1.73359 2.42763 -1.98275
H -0.53191 1.24437 -2.46952
H 2.39895 -3.78534 0.04123
H 2.92204 -2.65087 1.28437
H 1.21239 -2.79381 0.89577
H 4.15768 -2.73932 -1.30981
H 4.03798 -1.08352 -1.89498
H 4.50751 -1.39196 -0.22527
H 1.83193 -0.20728 3.83433
H 0.48853 -0.29789 2.67957
H 1.71099 -1.55939 2.70519
H 4.15665 0.23591 3.02783
H 4.03728 -1.09969 1.88760
H 4.50737 0.49999 1.31869
H 4.15570 2.50653 -1.71686
H 4.03691 2.18292 0.00932
H 4.50729 0.89314 -1.09462
H 2.39647 1.85810 -3.29879
H 2.92212 0.21490 -2.93862
H 1.21188 0.62039 -2.86729
H -4.17429 2.12670 2.14776
H -4.50545 1.23372 0.66222
H -4.06503 0.36968 2.13339
H -2.40692 -0.65576 -3.72576
H -1.11884 -1.22471 -2.65552
H -2.78984 -1.73783 -2.38864
H -2.40555 -2.89972 2.43037
H -1.11996 -1.68564 2.38976
H -2.79228 -1.20163 2.69915
H 1.83036 3.42470 -1.73701
H 0.48818 2.46973 -1.07896
H 1.71227 3.12286 -0.00129
H 2.39826 1.92857 3.25736
H 2.92268 2.43763 1.65363
H 1.21269 2.17336 1.97057
H 1.83364 -3.21694 -2.09752
H 0.48970 -2.17190 -1.59968
H 1.71315 -1.56300 -2.70381

3
NH_2^-_tzvp-disp
N 0.000000 0.000000 0.147095
H 0.000000 0.794003 -0.514832
H 0.000000 -0.794003 -0.514832

4
$LiNH_2$_tzvp-disp
N -0.328061 0.000016 -0.000057
H -0.950947 0.800321 0.000146
Li 1.399354 0.000020 0.000035
H -0.950685 -0.800492 0.000146

4
NH_3_tzvp-disp
N 0.000028 0.000000 -0.112648
H -0.471520 -0.814435 0.262820
H -0.469812 0.815421 0.262820
H 0.941133 -0.000985 0.262899

28
(Me$_3$Si)$_2$NH_tzvp-disp
Si -1.574916 0.008220 -0.086496
Si 1.574908 -0.008248 -0.086488
N -0.000008 -0.000167 -0.808262
C -2.074552 -1.699942 0.500477
H -2.071283 -2.411486 -0.329127
H -3.077340 -1.693760 0.936647
H -1.381742 -2.071570 1.258983
C -2.789536 0.593494 -1.385929
H -2.547098 1.603456 -1.725322
H -3.808501 0.606592 -0.990969
H -2.784268 -0.067199 -2.257390
C -1.588849 1.170572 1.382373
H -0.868536 0.866818 2.145837

H -2.575751 1.184671 1.852468
H -1.343397 2.189988 1.076470
C 1.588885 -1.169835 1.382988
H 0.869076 -0.865288 2.146618
H 2.575978 -1.184162 1.852677
H 1.342764 -2.189281 1.077744
C 2.789450 -0.594266 -1.385666
H 2.547212 -1.604561 -1.724219
H 3.808497 -0.606757 -0.990911
H 2.783806 0.065708 -2.257675
C 2.074621 1.700202 0.499541
H 2.072357 2.411097 -0.330618
H 3.077008 1.693994 0.936633
H 1.381267 2.072672 1.257139
H 0.000012 -0.000735 -1.818079

64
(*i*Pr$_3$Si)$_2$NH_tzvp-disp
Si 1.60293 -0.03082 -0.25640
Si -1.68022 0.02972 -0.01205
N -0.06753 0.28814 -0.60007
C 2.60763 1.32792 -1.13070
H 2.68620 0.97685 -2.17029
C 4.03144 1.48083 -0.59185
C 1.91393 2.68977 -1.15692
C 2.05453 -1.68576 -1.06943
C 1.32158 -2.85562 -0.41857
C 3.54169 -1.98655 -1.23946
H 1.63236 -1.56318 -2.07831
C 1.82202 -0.05096 1.61890
C 1.59725 1.33285 2.22934
C 3.13093 -0.66363 2.11385
H 1.01392 -0.70051 1.97142
C -2.60139 1.66322 -0.28416
C -1.93020 2.78581 0.50778
H -3.61908 1.52476 0.10060
C -2.70865 2.05404 -1.75726
C -2.48731 -1.38664 -0.98734
C -2.02760 -1.45789 -2.44287
C -4.01235 -1.38658 -0.88911
H -2.12465 -2.29905 -0.49634
C -1.70381 -0.38376 1.83990
C -1.38723 -1.84761 2.14452
C -3.02296 0.00205 2.51401
H -0.91824 0.23584 2.28492
H -2.47401 3.72932 0.39428
H -1.87163 2.56612 1.57604
H -0.90948 2.94690 0.15340
H -3.20867 3.02165 -1.87014
H -1.72120 2.15762 -2.21900
H -3.27118 1.32353 -2.34058
H -2.97893 -0.21058 3.58699
H -3.26054 1.05997 2.39797
H -3.86256 -0.56877 2.11012
H -1.28962 -2.00812 3.22299
H -2.18971 -2.50138 1.79300
H -0.46405 -2.18995 1.67601
H -4.44065 -2.25236 -1.40473
H -4.35934 -1.41862 0.14564
H -4.44152 -0.49206 -1.34802
H -2.45366 -2.33340 -2.94372
H -2.33957 -0.57916 -3.01303
H -0.94089 -1.53057 -2.51545
H 1.52754 1.27376 3.32044
H 2.42550 2.00592 1.99611
H 0.68325 1.80097 1.85941
H 3.16348 -0.66662 3.20834
H 3.24953 -1.69600 1.78141
H 4.00083 -0.10305 1.76644
H 2.50890 3.41655 -1.71998
H 0.92521 2.64717 -1.61636
H 1.78162 3.08773 -0.14838
H 4.61121 2.16784 -1.21673
H 4.02455 1.89617 0.41847
H 4.57350 0.53520 -0.55249
H 3.68652 -2.93872 -1.76094
H 4.04896 -1.21645 -1.82324
H 4.05588 -2.06497 -0.27902
H 1.48300 -3.78413 -0.97595
H 1.66930 -3.02926 0.60371
H 0.24487 -2.68079 -0.37905
H -0.13924 0.66329 -1.54096

82
(*t*Bu$_3$Si)$_2$NH_tzvp-disp
Si 1.740136 0.004575 0.005521
Si -1.730563 0.001860 -0.001852
N 0.004188 0.288787 -0.030725
C 2.361949 -0.622494 -1.722400
C 2.177516 -2.136939 -1.886167
C 3.847435 -0.335172 -1.971674
C 1.561034 0.066018 -2.833936
C 2.282730 -1.220363 1.393472
C 2.144751 -0.584635 2.782185
C 3.743130 -1.666269 1.253033
C 1.408479 -2.476863 1.362549
C 2.444040 1.786005 0.355221
C 2.325165 2.693646 -0.876396
C 3.920430 1.771998 0.766472
C 1.661668 2.477795 1.481160
C -2.293431 -1.588171 -0.955587
C -1.532896 -1.764284 -2.269753
C -3.788354 -1.542462 -1.298789
C -2.054316 -2.861478 -0.137785
C -2.368515 -0.055287 1.831235
C -1.577452 -1.053086 2.677340
C -3.848518 -0.428819 1.963210
C -2.155593 1.312725 2.489413
C -2.430079 1.597194 -0.886187
C -1.689302 2.872998 -0.458433
C -3.918426 1.835153 -0.607474
C -2.245436 1.506836 -2.405734
H -1.865523 -2.687279 -2.759287
H -1.695618 -0.947819 -2.969817
H -0.463832 -1.849188 -2.104930
H -2.323389 -3.732787 -0.746392
H -1.010270 -2.980428 0.151409
H -2.665943 -2.898750 0.763084
H -4.269705 2.675228 -1.218217
H -4.539629 0.974491 -0.851567
H -4.104372 2.098154 0.433922
H -2.189769 3.740343 -0.903503
H -1.665246 3.028097 0.618283
H -0.662824 2.900866 -0.830153
H -4.148323 -0.341021 3.014424
H -4.509287 0.213021 1.382381

H -4.034991 -1.461464 1.665842
H -1.928449 -1.004054 3.715047
H -1.691846 -2.083611 2.346498
H -0.518271 -0.815194 2.682317
H 2.589797 3.719750 -0.595990
H 2.996444 2.395378 -1.680491
H 1.314395 2.725265 -1.287727
H 4.277210 2.803564 0.870146
H 4.075942 1.283294 1.727928
H 4.558217 1.279632 0.033100
H 1.863484 -0.337930 -3.807191
H 0.492148 -0.097815 -2.722384
H 1.729929 1.141327 -2.861860
H 4.138162 -0.762115 -2.938992
H 4.064611 0.731398 -2.016858
H 4.495000 -0.778429 -1.214710
H 4.002190 -2.311346 2.100942
H 3.917907 -2.244858 0.347155
H 4.441055 -0.829220 1.257204
H 2.274258 -1.356185 3.549812
H 2.905514 0.175637 2.958083
H 1.173389 -0.120532 2.945122
H -4.072746 -2.491812 -1.767690
H -4.424288 -1.409209 -0.424630
H -4.024279 -0.752230 -2.010949
H -2.390075 1.241403 3.557983
H -1.118293 1.644036 2.405372
H -2.799541 2.085669 2.071175
H -2.522864 2.464873 -2.861260
H -1.208844 1.302486 -2.679973
H -2.875683 0.743316 -2.859877
H 1.672556 -3.128774 2.203663
H 0.353577 -2.237262 1.461052
H 1.542487 -3.053969 0.449395
H 2.352854 -2.411700 -2.932751
H 2.886441 -2.703346 -1.282799
H 1.178654 -2.482512 -1.625169
H 2.106402 3.461181 1.673337
H 0.617205 2.649069 1.220211
H 1.680663 1.920942 2.415455
H 0.004025 1.296549 -0.171288

References

1 N. Wiberg, G. Fischer and P. Karampatses, *Angew. Chem. Int. Ed.*, 1984, **23**, 59–60.

2 N. Wiberg, P. Karampatses, E. Kühnel, M. Veith and V. Huch, *Z. Anorg. Allg. Chem.*, 1988, **562**, 91–101.

3 S. Burnett, C. Bourne, A. M. Z. Slawin, T. van Mourik and A. Stasch, *Angew. Chem. Int. Ed.*, 2022, **61**, e202204472.

4 N. F. Chilton, C. A. P. Goodwin, D. P. Mills and R. E. P. Winpenny, *Chem. Commun.*, 2015, **51**, 101–103.

5 N. Wiberg, E. Kühnel, K. Schurz, H. Borrmann and A. Simon, *Z. Naturforsch. B*, 1988, **43**, 1075–1086.

6 J. Martin, C. Knüpfer, J. Eyselein, C. Färber, S. Grams, J. Langer, K. Thum, M. Wiesinger and S. Harder, *Angew. Chem. Int. Ed.*, 2020, **59**, 9102–9112.

7 M. J. Frisch, G. J. Trucks, H. B. Schlegel, G. E. Scuseria, M. A. Robb, J. R. Cheeseman, G. Scalmani, V. Barone, G. A. Petersson, H. Nakatsuji, X. Li, M. Caricato, A. V. Marenich, J. Bloino, B. G. Janesko, R. Gomperts, B. Mennucci, H. P. Hratchian, J. V. Ortiz, A. F. Izmaylov, J. L. Sonnenberg, D. Williams-Young, F. Ding, F. Lipparini, F. Egidi, J. Goings, B. Peng, A. Petrone, T. Henderson, D. Ranasinghe, V. G. Zakrzewski, J. Gao, N. Rega, G. Zheng, W. Liang, M. Hada, M. Ehara, K. Toyota, R. Fukuda, J. Hasegawa, M. Ishida, T. Nakajima, Y. Honda, O. Kitao, H. Nakai, T. Vreven, K. Throssell, J. A. Montgomery, J. E. Peralta, F. Ogliaro, M. J. Bearpark, J. J. Heyd, E. N. Brothers, K. N. Kudin, V. N. Staroverov, T. A. Keith, R. Kobayashi, J. Normand, K. Raghavachari, A. P. Rendell, C. Burant, S. S. Iyengar, J. Tomasi, M. Cossi, J. M. Millam, M. Klene, C. Adamo, R. Cammi, J. W. Ochterski, R. L. Martin, K. Morokuma, O. Farkas, J. B. Foresman and D. J. Fox, Gaussian 16 Rev. A.03 2016.

8 A. D. Becke, *J. Chem. Phys.*, 1993, **98**, 5648–5652.

9 J. P. Perdew, J. A. Chevary, S. H. Vosko, K. A. Jackson, M. R. Pederson, D. J. Singh and C. Fiolhais, *Phys. Rev. B*, 1992, **46**, 6671–6687.

10 F. Weigend and R. Ahlrichs, *Phys. Chem. Chem. Phys.*, 2005, **7**, 3297–3305.

11 F. Weigend, *Phys. Chem. Chem. Phys.*, 2006, **8**, 1057–1065.

12 S. Grimme, S. Ehrlich and L. Goerigk, *J. Comput. Chem.*, 2011, **32**, 1456–1465.

13 N. van Eikema-Hommes, Molecule 2016.

14 R. F. W. Bader, *Chem. Rev.*, 1991, **91**, 893–928.

15 T. A. Keith, AIMAll TK Gristmill Software, Overland Park, KS 2017.

16 T. D. Goddard, C. C. Huang, E. C. Meng, E. F. Pettersen, G. S. Couch, J. H. Morris and T. E. Ferrin, *Protein Sci.*, 2018, **27**, 14–25.

17 E. F. Pettersen, T. D. Goddard, C. C. Huang, E. C. Meng, G. S. Couch, T. I. Croll, J. H. Morris and T. E. Ferrin, *Protein Sci.*, 2021, **30**, 70–82.

18 A. J. Schaefer, V. M. Ingman and S. E. Wheeler, *J. Comput. Chem.*, 2021, **42**, 1750–1754.

19 a) Rigaku Oxford Diffraction, **2019**, CrysAlisPro Software system, version 1.171.40.67a, Rigaku Corporation, Oxford, UK (compounds ^{tBu}NH, (^{tBu}N)Cs (toluene)$_{3}$,); b) Rigaku Oxford Diffraction, **2020**, CrysAlisPro Software system, version 1.171.40.84a, Rigaku Corporation, Oxford, UK (compounds (^{iPr}N)Cs, (^{tBu}N)Cs, [ISr^{+}·(THF)$_{6}$][^{tBu}N^{-}], [Li^{+}·(12-crown-4)$_{2}$] [^{tBu}N^{-}], [Li^{+}·(PMDTA)(THF)] [^{tBu}N^{-}]); c) Rigaku Oxford Diffraction, **2021**, CrysAlisPro Software system, version 1.171.41.113a, Rigaku Corporation, Oxford, UK (compounds [K^{+} (18-crown-6)(THF)$_{2}$] [^{tBu}N^{-}], (^{iPr}N)Li (toluene)); d) Rigaku Oxford Diffraction, **2022**, CrysAlisPro Software system, version 1.171.42.72a, Rigaku Corporation, Oxford, UK (compounds [(BDI)Mg^{+}·(THF)$_{1.26}$(Et2O)$_{0.76}$][^{tBu}N^{-}], (^{tBu}N)Li, (^{iPr}N)Li.

20 O. V. Dolomanov, L. J. Bourhis, R. J. Gildea, J. A. K. Howard and H. Puschmann, *J. Appl. Cryst.*, 2009, **42**, 339–341.

21 G. M. Sheldrick, *Acta Cryst. A*, 2015, **71**, 3–8.

22 G. M. Sheldrick, *Acta Cryst. C*, 2015, **71**, 3–8.

23 A. Thorn, B. Dittrich and G. M. Sheldrick, *Acta Cryst. A*, 2012, **68**, 448–451.
